# Guaiazulene-Based 2‑Hydrazinylpyridine Probe (**GAHP550**) for Ultralow, Reversible Cu^2+^ Detection and Molecular Logic Gate Applications

**DOI:** 10.1021/acsomega.6c05174

**Published:** 2026-08-25

**Authors:** Mehmet Kaplan, Gabriele Kociok-Köhn, G. Dan Pantoş, Simon E. Lewis, Tony D. James

**Affiliations:** † Department of Chemistry, 1555 University of Bath, Claverton Down, Bath BA2 7AY, U.K.; ‡ Faculty of Science, Department of Chemistry, Ankara University, Ankara 06100, Turkiye; § Physical Structure Characterization, 1555 University of Bath, Claverton Down, Bath BA2 7AY, U.K.; ∥ School of Chemistry and Chemical Engineering, Henan Normal University, Xinxiang 453007, P. R. China

## Abstract

A guaiazulene-derived 2-hydrazinylpyridine probe (**GAHP550**) was developed for the highly sensitive, selective, and reversible detection of Cu^2+^ ions, with seamless integration into a smartphone-based analytical platform. The probe was synthesized via a simple two-step procedure from commercially available guaiazulene in 88.7% overall yield, without the need for chromatographic purification. The structure was confirmed using NMR, HRMS, FTIR, and single-crystal X-ray diffraction. **GAHP550** exhibits a rapid, visually perceptible green-to-brown color change upon Cu^2+^ binding, with a linear response between 5.0 × 10^–7^ and 1.2 × 10^–3^ M and an ultralow detection limit of 9.45 × 10^–7^ M (55.4 ppb), far below WHO (2.0 ppm) and EPA (1.3 ppm) recommended limits for drinking water. High selectivity was observed against a broad panel of competing cations and anions, and reversible sensing was demonstrated via multiple Cu^2+^/Na_4_EDTA cycles. The binding stoichiometry was determined as 1:2 (Cu^2+^: **GAHP550**) with a strong binding constant of 1.21 × 10^6^ M^–2^. Interestingly, the probe functions as an INHIBIT-type molecular logic gate using Cu^2+^ and Na_4_EDTA as inputs. A smartphone application was developed to quantify Cu^2+^ concentrations in real-time using RGB analysis of the colorimetric transition. **GAHP550** was then used to successfully quantify Cu^2+^ in tap and bottled water, achieving excellent recovery values of 83.5–104.2%, confirming the robustness and applicability in practical aqueous solutions. The integration of ultrahigh sensitivity, fast reversible response, logic gate functionality, and mobile-device compatibility establishes **GAHP550** as a powerful, portable tool for environmental and field-based Cu^2+^ detection.

## Graphical abstract

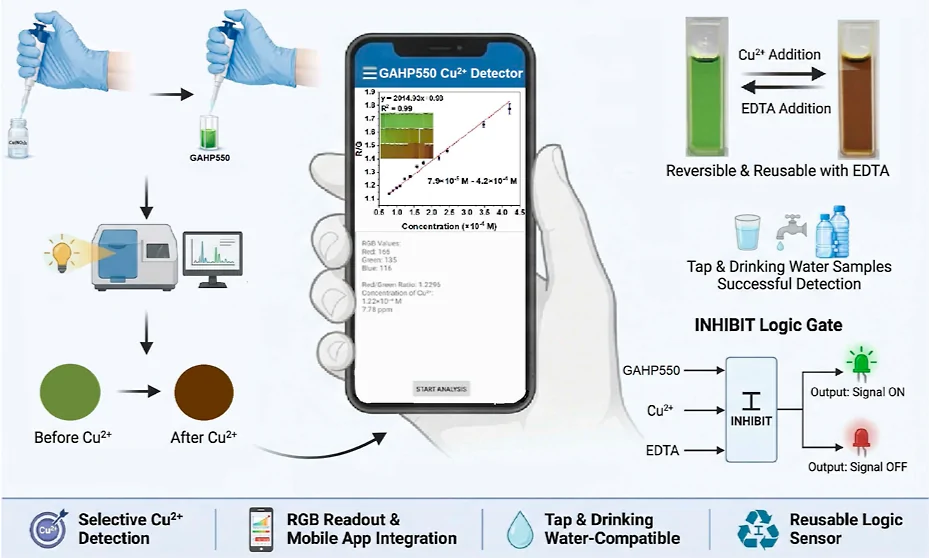

## 1 Introduction

Copper­(II) is the third most abundant essential metal ion in the human body after Fe^3+^ and Zn^2+^,^1–3^ and functions as a crucial cofactor in numerous metalloenzymes, including tyrosinase, superoxide dismutase,^2,4^ and cytochrome c oxidase.^2,4,5^ It plays indispensable roles in hemopoiesis, iron homeostasis, mitochondrial respiration, and redox regulation.^2,4–6^ Disruption of Cu^2+^ homeostasis can have severe pathological consequences. Both deficiency and excess of copper are implicated in neurodegenerative disorders (e.g., Alzheimer’s disease,^7–9^ Parkinson’s disease,^8^ Wilson’s^10^ and Menkes^9,10^ syndromes), cardiovascular and metabolic dysfunctions, hematological abnormalities, oxidative stress–related damage, skeletal malformations, and psychological conditions such as depression, anxiety, and cognitive impairment. Moreover, chronic overexposure to copper has been associated with carcinogenesis in liver, lung, oral, and prostate tissues.^5^

Beyond physiological relevance, Cu^2+^ is extensively utilized in industrial sectors including metallurgy, electronics, agriculture, construction, and energy materials. As such, improper waste management and industrial discharge can result in persistent Cu^2+^ accumulation in soil and aquatic ecosystems, posing substantial environmental and public health risks.^11–14^ Consequently, regulatory agencies have established strict safety limits for copper in drinking water—1.3 ppm (∼20 μM) by the United States Environmental Pollution Agency (EPA) and 2.0 ppm (∼30 μM) by the World Health Organization (WHO).^15,16^

A wide range of analytical techniques have been employed for Cu^2+^ determination, such as atomic absorption spectroscopy,^17,18^ inductively coupled plasma mass spectroscopy,^19^ inductively coupled plasma atomic emission spectrometry,^20,21^ high-performance liquid chromatography,^22^ and electrochemical methods.^23,24^ Although these approaches offer high sensitivity and selectivity, their practical deployment is limited by high instrumentation cost, need for trained operators, laborious sample preparation, and lack of portability.^25–28^ As a result, colorimetric and spectrophotometric chemosensors have garnered increasing attention as low-cost, rapid, and field-deployable alternatives for Cu^2+^ monitoring.^25,27–33^

Azulene, a non alternant bicyclic aromatic system comprising fused five- and seven-membered rings, exhibits exceptional physicochemical properties distinct from its naphthalene isomer.^34–38^ Its intrinsic polarization, high dipole moment, and narrow HOMO–LUMO gap impart characteristic blue coloration and strong visible-region absorption, making it an attractive scaffold for functional material design.^39,40^ Azulene derivatives have been successfully utilized in photonic materials, fluorescent and colorimetric sensors, molecular logic devices, and stimuli-responsive systems, with reported applications for CN^–^, Hg^2+^, Ag^+^, F^–^, NO_2_^–^, PO_4_^3–^, ATP, and reactive oxygen species detection.^34,35,37,38,41–50^ A pioneering early report from Wakabayashi et al. describes a colorimetric response to Cu^2+^ of some pyridylazulenes, but this was not quantified and selectivity over other transition metals (Pb^2+^, Hg^2+^, Cr^3+^) was minimal.^51^ Guaiazulene is a naturally occurring azulene-containing sesquiterpene which exhibits diverse bioactivities—anti-inflammatory, antioxidant, antimicrobial, and epithelial healing—and is an FDA-approved cosmetic ingredient. Guaiazulene derivatives have also been evaluated as food colorants.^52,53^ Compared to azulene, guaiazulene offers advantages as a starting material, including commercial availability on scale and low cost. These features, combined with guaiazulene’s modifiable chromophoric framework, have stimulated increasing interest in guaiazulene-based sensing platforms.^42,54,55^

Inspired by the principles of Boolean computation, molecular logic gates have emerged as chemical systems capable of executing logic functions (YES, NOT, AND, OR, INHIBIT, XOR, etc.) via analyte-induced signal variations.^56–59^ Since the pioneering work of de Silva,^60^ the field has advanced toward integrated optical logic circuits and multi-input molecular computation for chemical information processing and smart sensing applications.^29,59,61–63^ Examples of use of azulene in molecular logic gates reported to date include in half adders and full adders,^64–66^ as well as in AND and OR gates with chemical and optical inputs and electrical outputs.^67^

With this research, a guaiazulene-derived 2-hydrazinylpyridine-functionalized probe (**GAHP550**) was designed and synthesized for selective and sensitive Cu^2+^ detection. The probe offers high synthetic efficiency, requiring no chromatographic purification, and was fully characterized by NMR, HRMS, FTIR, and single-crystal X-ray diffraction. **GAHP550** demonstrates rapid and reversible response toward Cu^2+^ with an ultralow detection limit of 9.45 × 10^–7^ M (55.4 ppb), well below EPA and WHO regulatory limits. Distinct green-to-brown color transition enables naked-eye sensing, and accurate quantification was achieved for bottled and tap water samples with excellent recovery values. Additionally, **GAHP550** operates as an INHIBIT-type molecular logic gate and was successfully integrated into a smartphone-based analytical platform, highlighting its potential for portable environmental monitoring and real-world application.

## 2 Materials and Methods

### 2.1 Materials

Zinc nitrate hexahydrate [Zn­(NO_3_)_2_•6H_2_O], 2-hydrazinylpyridine, potassium acetate, deuterated dimethyl sulfoxide (DMSO-*d*_6_), deuterated chloroform (CDCl_3_) were purchased from Fluorochem Ltd. (Hadfield, England) and were used without any purification process. Lithium nitrate (LiNO_3_), sodium nitrate (NaNO_3_), potassium nitrate (KNO_3_), cesium nitrate (CsNO_3_), calcium nitrate hydrate [Ca­(NO_3_)_2_•*x*H_2_O], strontium nitrate [Sr­(NO_3_)_2_], barium nitrate [Ba­(NO_3_)_2_], chromium­(III) nitrate nonahydrate [Cr­(NO)_3_•9H_2_O], iron­(II) perchlorate hydrate [Fe­(ClO_4_)_2_•*x*H_2_O], cobalt­(II) nitrate hexahydrate [Co­(NO_3_)_2_•6H_2_O], nickel­(II) nitrate hexahydrate [Ni­(NO_3_)_2_•6H_2_O], copper­(II) nitrate hydrate [Cu­(NO_3_)_2_•*x*H_2_O], silver nitrate (AgNO_3_), mercury­(II) nitrate monohydrate [Hg­(NO_3_)_2_•H_2_O], aluminum nitrate nonahydrate [Al­(NO_3_)_3_•9H_2_O], potassium hydroxide (KOH), sodium sulfide (Na_2_S), sodium hydrosulfide hydrate [NaSH•*x*H_2_O], sodium sulfite (Na_2_SO_3_), sodium sulfate (Na_2_SO_4_), sodium bisulfite (NaHSO_3_), sodium hydrogen sulfate monohydrate (NaHSO_4_•H_2_O), sodium hydrosulfite (Na_2_S_2_O_4_), sodium nitrate (NaNO_3_), potassium thiocyanate (KSCN), sodium bicarbonate (NaHCO_3_), potassium carbonate (K_2_CO_3_), potassium dihydrogen phosphate (KH_2_PO_4_), disodium hydrogen phosphate (Na_2_HPO_4_), potassium permanganate (KMnO_4_), guaiazulene (99%), magnesium sulfate (MgSO_4_), sodium tetraborate decahydrate [Na_2_B_4_O_7_•10H_2_O], ethylenediaminetetraacetic acid tetrasodium salt dihydrate (Na_4_EDTA•2H_2_O) were purchased from Fisher Scientific (New Hampshire, USA) and were used without any purification process. Rubidium nitrate (RbNO_3_), ammonium nitrate (NH_4_NO_3_), magnesium nitrate hexahydrate [Mg­(NO_3_)_2_•6H_2_O], manganese­(II) nitrate hydrate [Mn­(NO_3_)_2_•*x*H_2_O], iron­(III) nitrate nonahydrate [Fe­(NO_3_)_3_•9H_2_O], cadmium nitrate tetrahydrate [Cd­(NO_3_)_2_•4H_2_O], tin­(II) chloride (SnCl_2_), tin­(IV) chloride (SnCl_4_), lead­(II) nitrate [Pb­(NO_3_)_2_], hydrazine monohydrate [NH_2_NH_2_•H_2_O], sodium fluoride (NaF), potassium chloride (KCl), potassium bromide (KBr), potassium iodide (KI), sodium hypochlorite solution (6–14% active chlorine) (NaClO), sodium thiosulfate (Na_2_S_2_O_3_), sodium metabisulfite (Na_2_S_2_O_5_), sodium nitrite (NaNO_2_), sodium acetate (CH_3_COONa), potassium phosphate tribasic (K_3_PO_4_), phosphorus­(V) oxychloride (POCl_3_), *N*,*N*-dimethylformamide (DMF), 2-amino-2-(hydroxymethyl)-1,3-propanediol (Trizma base), 2-[4-(2-hydroxyethyl)­piperazin-1-yl]­ethane-1-sulfonic acid (HEPES), sodium hydroxide (NaOH), pentane, ethyl acetate (EtOAc), glacial acetic acid (CH_3_CO_2_H), ethanol (EtOH), silica gel (high-purity grade, 200–400 mesh), thin layer chromatography (TLC) (silica gel 60 F-254 aluminum-backed) plates were purchased from Merck (Darmstadt, Germany) and all solvents and chemicals were used without any purification process. Dichloromethane (DCM) and hydrochloric acid (HCl) were purchased from VWR (Pennsylvania, USA).

### 2.2 Apparatus

UV–Vis spectroscopic measurements were performed at room temperature using an Agilent Cary 60 UV–Vis spectrophotometer and a BMG LabTech CLARIOstar microplate reader. A 1.0 cm path length quartz cuvette and 96-well flat-bottom microplates (Corning Costar) were employed for the measurements. During synthesis and solution preparation steps, an IKA RCT Basic magnetic stirrer hot plate, Sartorius analytical balance, Clifton ultrasonic bath, and Heidolph Hei-VAP Core rotary evaporator were utilized.

^1^H NMR and ^13^C NMR spectra were recorded on a Bruker Ascend 400 MHz spectrometer. High-resolution mass spectrometry (HRMS) analysis was conducted using an Agilent 6545 Q-TOF LC–MS system. Fourier-transform infrared (FTIR) spectra were acquired using a PerkinElmer Spectrum 100 FTIR spectrophotometer. The melting points were determined using a Stanford Research Systems OptiMelt Automated Melting Point Apparatus 100.

Smartphone-based image acquisition and processing were carried out using a Samsung Galaxy S24 Ultra (SM-S928B/DS) device.

### 2.3 X-ray Crystallography

Single crystals of **GAHP550**, grown from chloroform, were used for X-ray diffraction analysis. The resulting solid-state structure is depicted in Figure 2.

**Figure 1:**
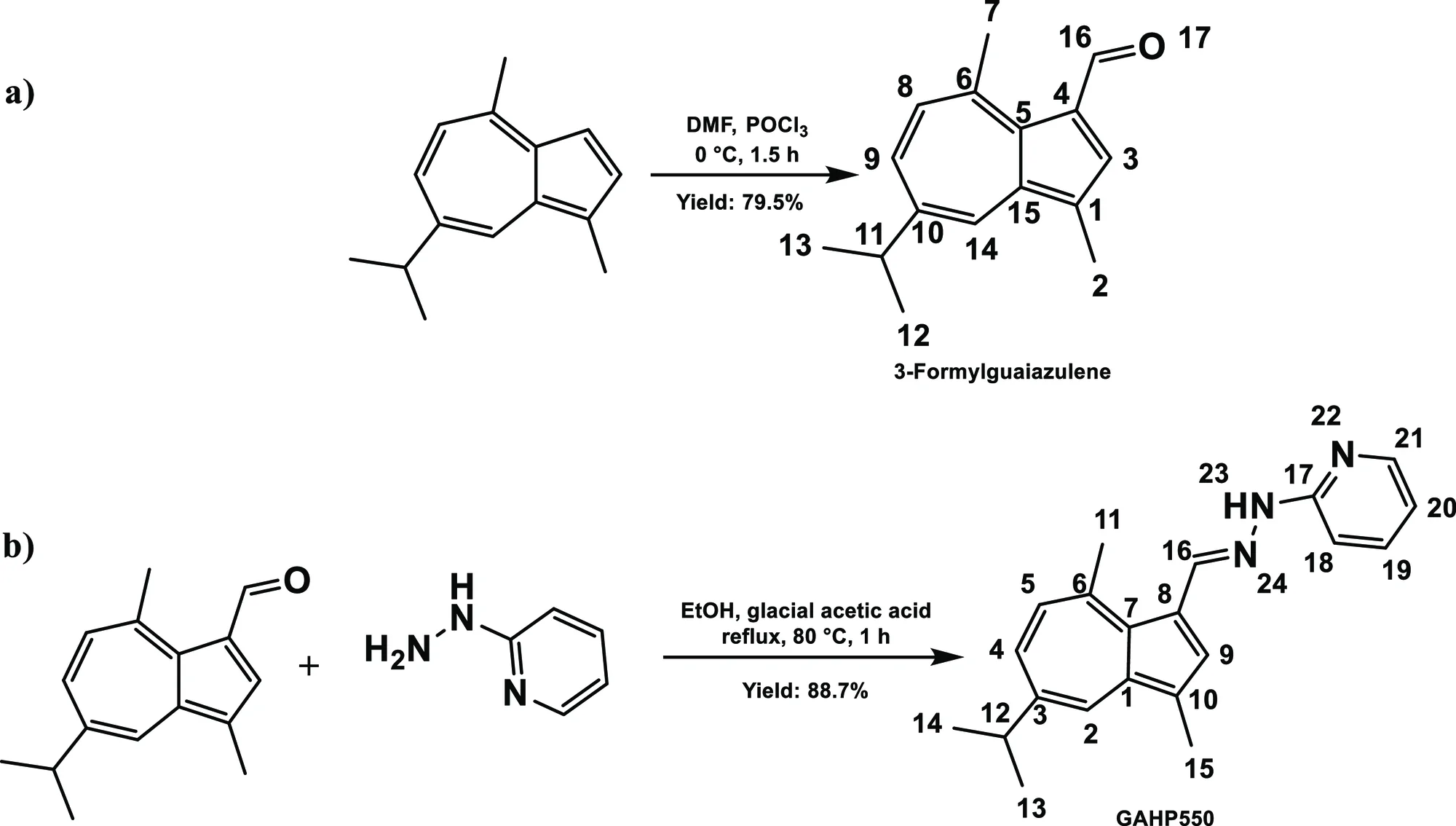
(a) Synthesis of 3-formylguaiazulene and (b) **GAHP550**.

**Figure 2:**
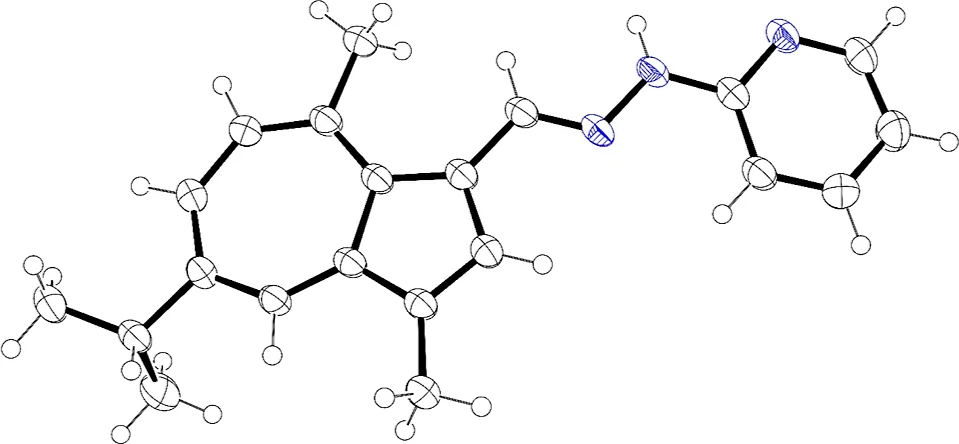
Solid-state structure of **GAHP550**. Thermal ellipsoids are drawn at 50% probability, and hydrogen atoms are depicted as spheres of arbitrary radius (CCDC: 2503754).

Single-crystal X-ray diffraction data were collected on a Rigaku XtaLAB Synergy, Dualflex diffractometer equipped with a Cu microfocus source (λ = 1.54184 Å) and a HyPix-Arc 100 detector. Unit cell determination, data collection, data reduction, and multiscan (symmetry-related) absorption correction were performed using CrysAlisPro software.^68^ The crystal structure was solved using SHELXT and refined through full-matrix least-squares refinement on *F* ^2^ using SHELXL-2019/3.^69^ All non-hydrogen atoms were refined anisotropically. Hydrogen atoms were positioned geometrically and refined using a riding model.

### 2.4 Synthesis of **GAHP550**

As shown in Figure 1, the probe was obtained in two steps. The 3-formylguaiazulene obtained in the first step was synthesized based on a literature procedure,^70^ and detailed information regarding its synthesis and characterization is provided in the Supporting Information (Figure 1a).

In the second step, 226.3 mg (1.0 mmol) of 3-formylguaiazulene was dissolved in 10 mL of EtOH in a round-bottom flask, and 120.1 mg (1.1 mmol) of 2-hydrazinylpyridine along with 60.0 μL (1.0 mmol) of glacial acetic acid were added to the solution. The reaction mixture was refluxed at 80 °C for 1 h. After cooling to room temperature, the solvent was removed under reduced pressure. The obtained product was then recrystallized from EtOH, affording **GAHP550** as a dark green solid (Figure 1b) (281.5 mg, 88.7% yield). *R*_f_: 0.30 (SiO_2_, 3:1 Pentane/EtOAc); mp = 160–162 °C; ^1^H NMR (DMSO-*d*_6_, 400 MHz): δ = 10.60 (s, 1H, H-23), 8.93 (s, 1H, H-16), 8.15 (s, 1H, H-9), 8.07 (d, *J* = 4.1 Hz, 1H, H-21), 8.01 (d, *J* = 2.1 Hz, 1H, H-2) 7.60 (app t, *J* = 7.7 Hz, 1H, H-19), 7.33 (dd, *J* = 10.4, 2.1 Hz, 1H, H-4), 7.24 (d, *J* = 8.4 Hz, 1H, H-18), 6.95 (d, *J* = 10.5 Hz, 1H, H-5), 6.69 (dd, *J* = 7.1, 4.9 Hz, 1H, H-20), 3.03 (hept, *J* = 6.8 Hz, 1H, H-12), 2.89 (s, 3H, H-11), 2.57 (s, 3H, H-15), 1.29 (d, *J* = 6.8 Hz, 6H, H-13, H-14) ppm; ^13^C NMR (DMSO-*d*_6_, 101 MHz): δ = 157.3 (C-17), 147.8 (C-21), 145.1 (C-6), 141.8 (C-3), 140.9 (C-1), 138.3 (C-16), 137.7 (C-19), 135.2 (C-9), 134.9 (C-4), 133.7 (C-2), 132.2 (C-7), 127.6 (C-5), 126.2 (C-10), 123.5 (C-8), 114.0 (C-20), 106.0 (C-18), 36.8 (C-12), 28.4 (C-11), 24.2 (C-13, C-14), 12.7 (C-15) ppm; IR (ATR): *v*_max_/cm^–1^ (neat) = 3181, 2944, 2856, 1597, 1536, 1441, 1312, 1282, 1137, 1087, 990, 898, 810, 761, 682, 649; HRMS (ESI^+^) *m*/*z*: [(C_21_H_23_N_3_) + H]^+^ theoretical: 318.1965; found, 318.1964.

### 2.5 Preparation of Solutions for Spectral Measurements

Stock solutions of all ions (Li^+^, Na^+^, K^+^, Rb^+^, Cs^+^, NH_4_^+^, Mg^2+^, Ca^2+^, Sr^2+^, Ba^2+^, Cr^3+^, Mn^2+^, Fe^2+^, Fe^3+^, Co^2+^, Ni^2+^, Cu^2+^, Zn^2+^, Cd^2+^, Ag^+^, Hg^2+^, Al^3+^, Sn^2+^, Sn^4+^, Pb^2+^, N_2_H_4_, F^–^, Cl^–^, Br^–^, I^–^, OH^–^, ClO^–^, S^2–^, SH^–^, SO_3_^2–^, SO_4_^2–^, HSO_3_^–^, HSO_4_^–^, S_2_O_3_^2–^, S_2_O_4_^2–^, S_2_O_5_^2–^, NO_2_^–^, NO_3_^–^, SCN^–^, CH_3_COO^–^, HCO_3_^–^, CO_3_^2–^, H_2_PO_4_^–^, HPO_4_^2–^, PO_4_^3–^, MnO_4_^–^) were prepared in distilled water at a concentration of 5.0 × 10^–2^ M. The solubility of **GAHP550** in various water-miscible organic solvents was assessed. **GAHP550** was observed to be only sparingly soluble in EtOH, MeOH, MeCN, DMF, and THF. In contrast, **GAHP550** exhibited good solubility in DMSO, which was accordingly selected as the organic cosolvent for the measurements. A linear calibration graph of the **GAHP550** probe was obtained in accordance with the Lambert–Beer law, and the optimal working concentration for **GAHP550** was determined to be 1.0 × 10^–3^ M (Figure S2). 0.1 M HEPES buffer at pH 7.4 was selected as the aqueous component of the measurement solvent, as it provides the optimal response upon the addition of Cu^2+^, ensures suitable selectivity, and does not alter the intrinsic response of the probe (Figure S3). Additionally, experiments with various proportions of 0.1 M HEPES buffer at pH 7.4 revealed that solubility decreases with increasing buffer content, and the optimal buffer proportion was confirmed to be 15% (Figure S4). Thus, the optimal working conditions were determined to be 1.0 × 10^–3^ M **GAHP550** with a binary solvent mixture consisting of 17:3 (v/v) DMSO/pH 7.4 HEPES (0.1 M).

Titration studies were conducted by adding aqueous Cu­(NO_3_)_2_ to the medium at concentrations ranging from 5.0 × 10^–7^ to 1.2 × 10^–3^ M. In selectivity and interference studies, group 1A, 2A, and 3A cations were used at 20 equiv, anions at 10 equiv, and transition metals at 5 equiv. Reversibility, reusability, and logic gate applications were performed using 5.0 × 10^–4^ M Cu­(NO_3_)_2_ and 6.6 × 10^–4^ M Na_4_EDTA, respectively. For stability studies, the **GAHP550** probe was stored in an amber flask at room temperature in a laboratory environment under a DMSO: H_2_O 17:3, v/v HEPES buffer at pH 7.4 mixture. Measurements were taken over a 100 day period, and the results were recorded. In response time studies, 5.0 × 10^–4^ M Cu­(NO_3_)_2_ was added to the medium containing the **GAHP550** probe, and measurements were recorded over a period of 10 min. All experiments were conducted in triplicate.

### 2.6 Smartphone Application

In the smartphone application study, 1.0 × 10^–3^ M **GAHP550** probe (DMSO/H_2_O 17:3, v/v, HEPES, pH 7.4, 0.1 M) was used, and aqueous Cu­(NO_3_)_2_ was added to the medium at concentrations ranging from 7.9 × 10^–5^ M to 4.2 × 10^–4^ M. Photographs were taken using a Samsung Galaxy S24 Ultra (SM-S928B/DS) mounted on a tripod within a mini photo studio to minimize potential vibrations and changes in lighting conditions. After each photograph was taken, three distinct points were selected, and the red/green/blue (RGB) values of these points were automatically calculated using a Python script. An increase in the green/red value was observed with increasing concentration, and a calibration graph was generated. Using the slope, intercept, and linear working range obtained from the calibration data, a mobile application named “**GAHP550** Cu^2+^ Detector” was developed.

### 2.7 Determination of Cu^2+^ in Real Samples

In the real sample studies, tap water collected directly from the laboratory and two different bottled drinking water samples purchased from a supermarket in Bath, United Kingdom, were used. To perform the analyses, 5.0 × 10^–2^ M Cu­(NO_3_)_2_ solutions were prepared using the respective water samples, and all necessary dilutions were also made with the same water sources. All real sample analyses were performed in triplicate using a UV–vis spectrophotometer.

### 2.8 Preparation of Cotton Buds

For the colorimetric selectivity and titration studies using cotton buds, a 1.0 × 10^–3^ M stock solution of **GAHP550** was first prepared in DMSO/pH 7.4 HEPES (0.1 M) (17:3, v/v). The cotton buds were then immersed in this solution for 1 min to allow complete impregnation with the probe. After impregnation, the cotton buds were removed and allowed to dry at room temperature. For the titration study, 5.0 mL aqueous stock solutions of Cu­(NO_3_)_2_.3H_2_O were prepared at 0.10, 0.25, 0.50, 0.75, 1.00, 1.50, 2.00, 3.00, 4.00, 5.00, 6.00, 7.00, 8.00, 9.00, and 10.00 equiv. The probe-impregnated cotton buds were immersed in each Cu^2+^ solution for 30 s, removed, and immediately photographed. For the colorimetric selectivity study, all competing ions were prepared as 5.0 mL solutions at 5.0 equiv. The probe-coated cotton buds were immersed individually in each competing ion solution for 30 s before being removed and photographed. All photographs were acquired using a Samsung Galaxy S24 Ultra (SM-S928B/DS) mounted on a tripod inside a mini photo studio to minimize the effects of vibration and fluctuations in ambient lighting.

## 3 Results and Discussion

### 3.1 Design, Synthesis and Structural Characterization of **GAHP550**

A guaiazulene-derived 2-hydrazinylpyridine-modified probe, designated **GAHP550**, was designed for the selective, sensitive, and colorimetric detection of Cu^2+^ ions. As illustrated in Figure 1, **GAHP550** was synthesized in two steps. First, 3-formylguaiazulene was obtained from commercially available guaiazulene following a reported procedure and structural confirmation was achieved through ^1^H NMR, ^13^C NMR, and HRMS analysis (Figures S5–S7).^70^ In the second step, condensation of 3-formylguaiazulene with 2-hydrazinopyridine in ethanol in the presence of glacial acetic acid afforded the target probe **GAHP550** in a high yield of 88.7%, in 1 h. Notably, the product was obtained in pure form without the need for chromatographic purification, requiring only recrystallization.

Structural confirmation was achieved using ^1^H NMR, ^13^C NMR, COSY, NOESY, HSQC, HMBC, FT-IR, HRMS (Figures S8–S15), and single-crystal X-ray diffraction (Figure S28 and Tables S1–S8) analyses. Diagnostic changes in the ^1^H NMR spectra were observed, with the aldehyde signal in the starting material (δ = 10.64 ppm) being replaced by the hydrazone signal in **GAHP550** (δ = 8.93 ppm), as well as the appearance of the N–H signal (δ = 10.60 ppm). Analogously, in the ^13^C NMR spectra, the aldehyde signal (δ = 186.4 ppm) shifts significantly upfield upon hydrazone formation (δ = 138.3 ppm).

### 3.2 Spectral Response to Cu^2+^ and other Competitive Ions

The spectral response of the **GAHP550** probe in the presence of various ions was examined under the conditions of DMSO/H_2_O (17:3, v/v, HEPES, pH 7.4). As shown in Figure 3, the probe exhibits a maximum absorbance peak around 640 nm, which shifts to approximately 550 nm upon the addition of Cu^2+^. This spectral change was accompanied by a visible color transition of the probe from green to brown with increasing concentrations of Cu^2+^. In contrast, no significant spectral changes were observed in the presence of competing cations and anions. The bar graph presented in Figure 3 clearly demonstrates that the **GAHP550** probe exhibits high selectivity toward Cu^2+^ ions. Additionally, to evaluate the stability of the probe, its spectrophotometric response was monitored over a 100 day period. As shown in Figure S18, the response of **GAHP550** remained constant throughout this period, indicating its suitability for long-term use and reliable Cu^2+^ detection.

**Figure 3:**
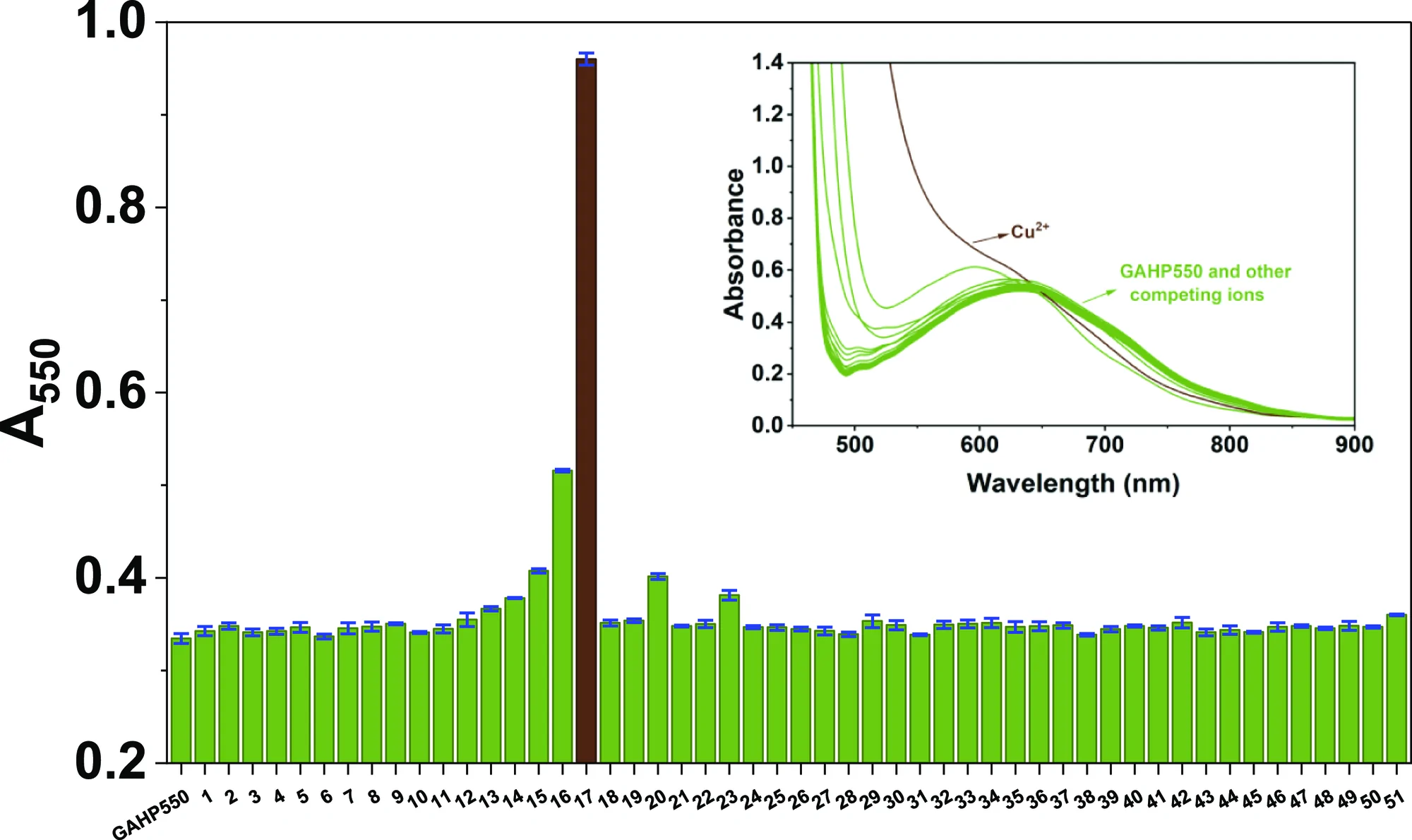
Bar graph presents the response of **GAHP550** (1.0 × 10^–3^ M) in the presence of Cu^2+^ and various potentially competing ions (1: Li^+^, 2: Na^+^, 3: K^+^, 4: Rb^+^, 5: Cs^+^, 6: NH_4_^+^, 7: Mg^2+^, 8: Ca^2+^, 9: Sr^2+^, 10: Ba^2+^, 11: Cr^3+^, 12: Mn^2+^, 13: Fe^2+^, 14: Fe^3+^, 15: Co^2+^, 16: Ni^2+^, 17: Cu^2+^, 18: Zn^2+^, 19: Cd^2+^, 20: Ag^+^, 21: Hg^2+^, 22: Al^3+^, 23: Sn^2+^, 24: Sn^4+^, 25: Pb^2+^, 26: N_2_H_4_, 27: F^–^, 28: Cl^–^, 29: Br^–^, 30: I^–^, 31: OH^–^, 32: ClO^–^, 33: S^2–^, 34: SH^–^, 35: SO_3_^2–^, 36: SO_4_^2–^, 37: HSO_3_^–^, 38: HSO_4_^–^, 39: S_2_O_3_^2–^, 40: S_2_O_4_^2–^, 41: S_2_O_5_^2–^, 42: NO_2_^–^, 43: NO_3_^–^, 44: SCN^–^, 45: CH_3_COO^–^, 46: HCO_3_^–^, 47: CO_3_^2–^, 48: H_2_PO_4_^–^, 49: HPO_4_^2–^, 50: PO_4_^3–^, 51: MnO_4_^–^). The inset displays the corresponding UV–vis absorption profiles recorded after sequential introduction of Cu^2+^ and competing ions. Measurements were performed in a DMSO/H_2_O (17:3, v/v) medium buffered with 0.1 M HEPES at pH 7.4. Ions numbered 1–10, 27–31, 42, 43, and 45–51 were used at 20 equiv; ions numbered 11–16, 18–26, 32–41, and 44 were used at 5 equiv, while ion 17 (Cu^2+^) was used at 1 equiv. Error bars represent the standard deviation of three independent measurements (*n* = 3).

The colorimetric selectivity of **GAHP550** toward Cu^2+^ can be clearly observed in Figure 4. Initially green in color, the **GAHP550** probe retains its green hue in the presence of all other competing analytes, whereas in the presence of Cu^2+^, it immediately changes to brown.

**Figure 4:**
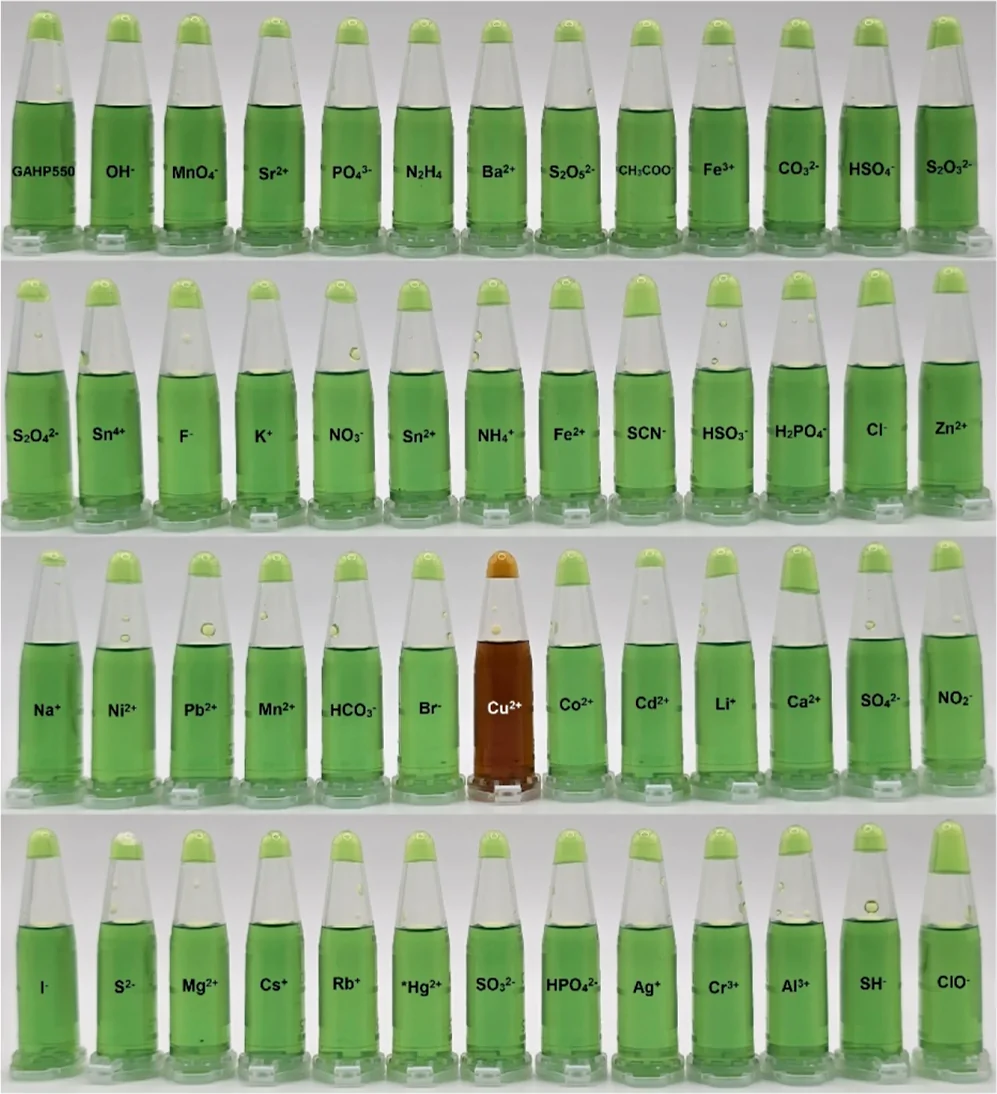
Distinct visual color variations were recorded when **GAHP550** (1.0 × 10^–3^ M) was treated with Cu^2+^ and a series of competing ions in a DMSO/H_2_O (17:3, v/v) system buffered with 0.1 M HEPES at pH 7.4. Li^+^, Na^+^, K^+^, Rb^+^, Cs^+^, NH_4_^+^, Mg^2+^, Ca^2+^, Sr^2+^, Ba^2+^, F^–^, Cl^–^, Br^–^, I^–^, OH^–^, NO_2_^–^, NO_3_^–^, CH_3_COO^–^, HCO_3_^–^, CO_3_^2–^, H_2_PO_4_^–^, HPO_4_^2–^, PO_4_^3–^, and MnO_4_^–^ were used at 20 equiv, whereas Cr^3+^, Mn^2+^, Fe^2+^, Fe^3+^, Co^2+^, Ni^2+^, Zn^2+^, Cd^2+^, Ag^+^, Hg^2+^, Al^3+^, Sn^2+^, Sn^4+^, Pb^2+^, N_2_H_4_, ClO^–^, S^2–^, SH^–^, SO_3_^2–^, SO_4_^2–^, HSO_3_^–^, HSO_4_^–^, S_2_O_3_^2–^, S_2_O_4_^2–^, S_2_O_5_^2–^ and SCN^–^ were used at 5 equiv. Cu^2+^ was used at 1 equiv.

Additionally, the titration study revealed that increasing equivalents of Cu^2+^ in the medium led to a corresponding increase in absorbance at 550 nm (Figure 5). Based on the obtained linear calibration graph, the limit of detection (LOD) was calculated using the formula LOD = 3 × SD/S, resulting in a value of 9.45 × 10^–7^ M (55.4 ppb). Here, SD represents the standard deviation of blank measurements (0.00049), and S is the slope of the linear calibration graph.

**Figure 5:**
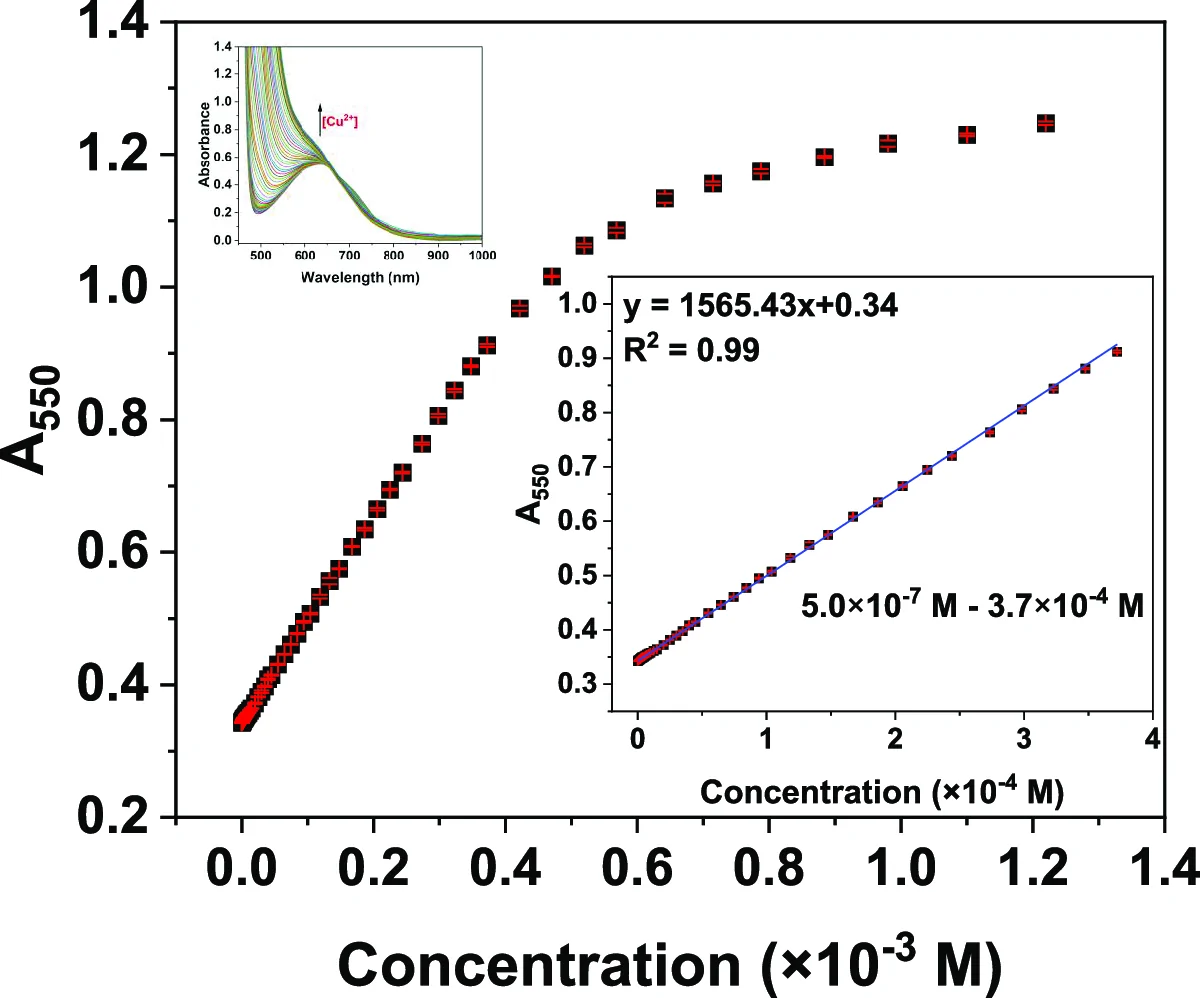
Titration profile of **GAHP550** (1.0 × 10^–3^ M) upon incremental addition of Cu^2+^, demonstrating a progressive absorbance enhancement with increasing ion concentration. Inset: UV–vis spectra recorded during the titration, along with a linear calibration curve obtained over the concentration range of 5.0 × 10^–7^–3.7 × 10^–4^ M. Experimental medium: DMSO/H_2_O (17:3, v/v) containing 0.1 M HEPES buffer at pH 7.4. Error bars represent the standard deviation of three independent measurements (*n* = 3).

To investigate the binding mode between **GAHP550** and Cu^2+^ ions, a Job’s plot analysis was performed. In this study, the total concentration was kept constant at 1.0 × 10^–4^ M, with varying molar ratios of Cu^2+^. As shown in the resulting graph (Figure S19), the binding mode was determined to be 1:2 (Cu^2+^/**GAHP550**). Based on the identified binding stoichiometry and the UV–vis data obtained from the titration experiment,^71^ the binding constant was calculated to be 1.21 × 10^6^ M^–2^ (Figure S20).

### 3.3 Examination of Interference Effects

An interference study was conducted to determine whether the spectrophotometric and colorimetric changes observed for **GAHP550** in the presence of Cu^2+^ ions could also occur in the presence of other potential competing ions. In this study, the addition of various competing ions (Li^+^, Na^+^, K^+^, Rb^+^, Cs^+^, NH_4_^+^, Mg^2+^, Ca^2+^, Sr^2+^, Ba^2+^, F^–^, Cl^–^, Br^–^, I^–^, OH^–^, NO_2_^–^, NO_3_^–^, CH_3_COO^–^, HCO_3_^–^, CO_3_^2–^, H_2_PO_4_^–^, HPO_4_^2–^, PO_4_^3–^, and MnO_4_^–^ (20 equiv); Cr^3+^, Mn^2+^, Fe^2+^, Fe^3+^, Co^2+^, Ni^2+^, Zn^2+^, Cd^2+^, Ag^+^, Hg^2+^, Al^3+^, Sn^2+^, Sn^4+^, Pb^2+^, N_2_H_4_, ClO^–^, S^2–^, SH^–^, SO_3_^2–^, SO_4_^2–^, HSO_3_^–^, HSO_4_^–^, S_2_O_3_^2–^, S_2_O_4_^2–^, S_2_O_5_^2–^, and SCN^–^ (5 equiv)) to the Cu^2+^-containing medium showed no significant interference effects, confirming that Cu^2+^ can be accurately detected by **GAHP550** even in the presence of these ions (Figure 6). Only a partial interference was observed in the presence of Hg^2+^; however, this effect was successfully masked by the addition of Na_2_S_2_O_3_ to the medium.

**Figure 6:**
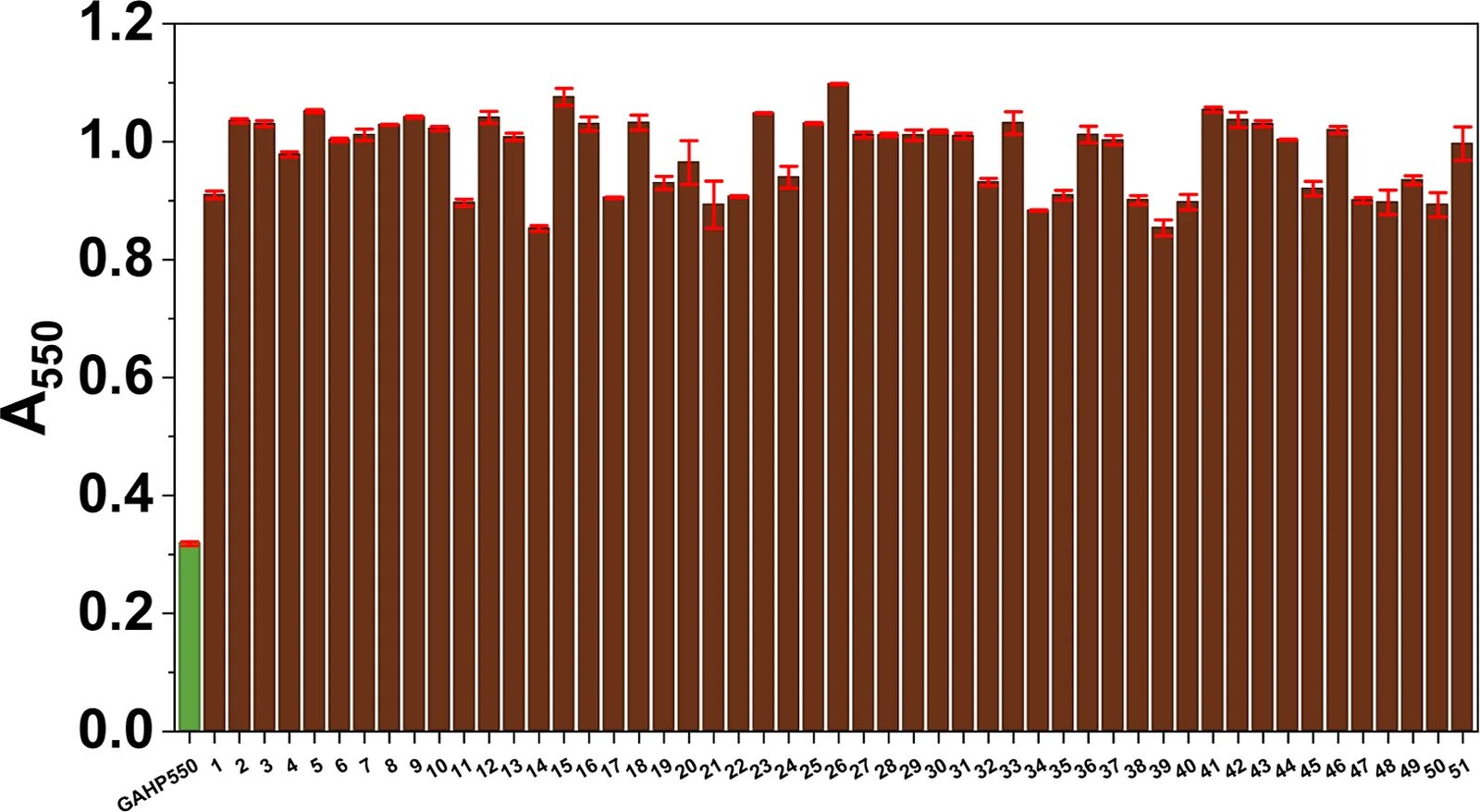
Interference experiments were conducted by introducing Cu^2+^ into **GAHP550** (1.0 × 10^–3^ M) solutions containing various potentially competing ions (1: Li^+^ + Cu^2+^, 2: Na^+^ + Cu^2+^, 3: K^+^ + Cu^2+^, 4: Rb^+^ + Cu^2+^, 5: Cs^+^ + Cu^2+^, 6: NH_4_^+^ + Cu^2+^, 7: Mg^2+^ + Cu^2+^, 8: Ca^2+^ + Cu^2+^, 9: Sr^2+^ + Cu^2+^, 10: Ba^2+^ + Cu^2+^, 11: Cr^3+^ + Cu^2+^, 12: Mn^2+^ + Cu^2+^, 13: Fe^2+^ + Cu^2+^, 14: Fe^3+^ + Cu^2+^, 15: Co^2+^ + Cu^2+^, 16: Ni^2+^ + Cu^2+^, 17: Cu^2+^, 18: Zn^2+^ + Cu^2+^, 19: Cd^2+^ + Cu^2+^, 20: Ag^+^ + Cu^2+^, 21: Hg^2+^ + Cu^2+^, 22: Al^3+^ + Cu^2+^, 23: Sn^2+^ + Cu^2+^, 24: Sn^4+^ + Cu^2+^, 25: Pb^2+^ + Cu^2+^, 26: N_2_H_4_ + Cu^2+^, 27: F^–^ + Cu^2+^, 28: Cl^–^ + Cu^2+^, 29: Br^–^ + Cu^2+^, 30: I^–^ + Cu^2+^, 31: OH^–^ + Cu^2+^, 32: ClO^–^ + Cu^2+^, 33: S^2–^ + Cu^2+^, 34: SH^–^ + Cu^2+^, 35: SO_3_^2–^ + Cu^2+^, 36: SO_4_^2–^ + Cu^2+^, 37: HSO_3_^–^ + Cu^2+^, 38: HSO_4_^–^ + Cu^2+^, 39: S_2_O_3_^2–^ + Cu^2+^, 40: S_2_O_4_^2–^ + Cu^2+^, 41: S_2_O_5_^2–^ + Cu^2+^, 42: NO_2_^–^ + Cu^2+^, 43: NO_3_^–^ + Cu^2+^, 44: SCN^–^ + Cu^2+^, 45: CH_3_COO^–^ + Cu^2+^, 46: HCO_3_^–^ + Cu^2+^, 47: CO_3_^2–^ + Cu^2+^, 48: H_2_PO_4_^–^ + Cu^2+^, 49: HPO_4_^2–^ + Cu^2+^, 50: PO_4_^3–^ + Cu^2+^, 51: MnO_4_^–^ + Cu^2+^). The measurements were performed in a DMSO/H_2_O (17:3, v/v) mixture buffered with 0.1 M HEPES at pH 7.4. Ions numbered 1–10, 27–31, 42, 43, and 45–51 were used at 20 equiv; ions numbered 11–16, 18–26, 32–41, and 44 were used at 5 equiv, while ion 17 (Cu^2+^) was used at 1 equiv. To suppress interference from Hg^2+^, Na_2_S_2_O_3_ was employed at a 4-fold molar excess relative to Hg^2+^ ions (entry 21). Error bars represent the standard deviation of three independent measurements (*n* = 3).

In addition to evaluating the interference effects of individual competing analytes on the response of **GAHP550** toward Cu^2+^, a mixed-ion system containing all potential interfering species was also evaluated. To eliminate the possible interference caused by Hg^2+^, Na_2_S_2_O_3_ was added to the medium. The results demonstrated that even in the presence of mixed ions at different equivalents, Cu^2+^ could still be successfully detected using the **GAHP550** probe (Figure S21).

### 3.4 Determination of Response Time

To determine the response time for Cu^2+^ detection using the **GAHP550** probe, UV–vis measurements were recorded using a microplate reader at 10 s intervals over a 10 min period. Upon the addition of Cu^2+^ to the probe solution, an increase in absorbance at 550 nm was observed, accompanied by a rapid (<10 s) color change from green to brown, which remained nearly constant throughout the 10 min period (Figure S22). Based on these results, it can be concluded that Cu^2+^ detection with the **GAHP550** probe occurs near-instantaneously and represents a more rapid response compared to the previous systems outlined in Table 1.

**Table 1:** Comparison of Analytical Performance Parameters of **GAHP550** with Previously Reported Cu^2+^-Detecting Probes

probe no	solvent medium	colorimetric change	response time	limit of detection (LOD)	smartphone measurements		reversible and reusable	logic gate application	refs
					without dedicated app	with dedicated app			
1	THF/H_2_O (1:1, v/v, pH 7.4 tris buffer)	yes	45 s	2.07 × 10^–7^ M	yes	no	yes	no	^72^
2	0.02 M citrate-sodium citrate buffer	yes	6 min	3.60 × 10^–7^ M	yes	no	no	no	^73^
3	DMSO/H_2_O (1:9, v/v, pH 7.4 HEPES)	yes	1 min	1.48 × 10^–7^ M	yes	no	yes	no	^74^
4	acetone/H_2_O (2:3, v/v, pH 7.4 HEPES 0.01 M)	yes	200 s	3.08 × 10^–7^ M	no	no	yes	no	^75^
5	EtOH/H_2_O (4:1, v/v, pH 6.3, 20 mM HEPES)	no	5 min	3.30 × 10^–7^ M	no	no	yes	no	^76^
6	DMSO: CH_3_CN (7:3, v/v)	yes	-	4.80 × 10^–7^ M	no	no	yes	no	^77^
7	MeOH/H_2_O (9:1, v/v, pH 7.0 PBS 10 mM)	yes	-	5.50 × 10^–7^ M	no	no	yes	no	^78^
8	Tris–HCl/ethanol (1:1, v/v, pH 7.4)	yes	∼50 min	6.23 × 10^–7^ M	no	no	no	no	^79^
9	MeOH: H_2_O (1:1, v/v, pH 7.2 Tris)	yes	-	2.04 × 10^–7^ M	no	no	yes	yes	^80^
10	DMSO/H_2_O (17:3, v/v, pH 7.4 HEPES 0.1 M)	yes	<10 s	9.45 × 10^–7^ M	yes	yes	yes	yes	this work

### 3.5 Reversibility and Reusability

To evaluate the reversibility of **GAHP550** colorimetric and spectrophotometric response toward Cu^2+^, Na_4_EDTA (0.67 equiv) was added following the introduction of Cu^2+^ ions (0.5 equiv). The formation of the Cu^2+^–EDTA complex resulted in a substantial, although partial, recovery of both the absorbance and colorimetric responses. Based on absorbance measurements, the recovery efficiency per cycle ranged from 75.7% to 95.5%, with an average of approximately 83.5%. Subsequent readdition of Cu^2+^ reproduced comparable responses. As shown in Figure S23, this reversible cycle was successfully repeated seven times, demonstrating the good reversibility and reusability of the probe.

### 3.6 Proposed Binding Mode between **GAHP550** and Cu^2+^

The previously obtained Job’s plot analysis (Figure S19) indicated a 1:2 metal/ligand binding stoichiometry, consistent with the formation of a **Cu­(GAHP550)**_**2**_ complex. This assignment was further supported by HRMS analysis (Figure S24). In addition, the ^1^H, COSY, and NOESY NMR spectra (Figures S16 and S17) of **Cu­(GAHP550)**_**2**_ confirm the 1:2 metal/ligand stoichiometry and indicate that the complex is not symmetric, with the two **GAHP550** ligands being inequivalent. In order to further explore the structure of this complex we undertook DFT studies (BHLYP/D3BJ/def2-TZVP/SMD­(H_2_O))^81–86^ using a penta-coordinated Cu­(II) center with 2 × 2 co-ordination sites being occupied by the N-donor ligands of the **GAHP550** (pyridine N and –N=C–) while the fifth co-ordination site is occupied by an aqua ligand. Of the four different optimized geometries (Figure S29) the structure with the lowest energy (Table S9) is consistent with the ^1^H NMR data and the UV–vis spectrum predicted via TDDFT (BHLYP/D3BJ/def2-TZVP/SMD­(H_2_O)) has a high similarity factor when compared with the experimental spectrum (Figure 7).

**Figure 7:**
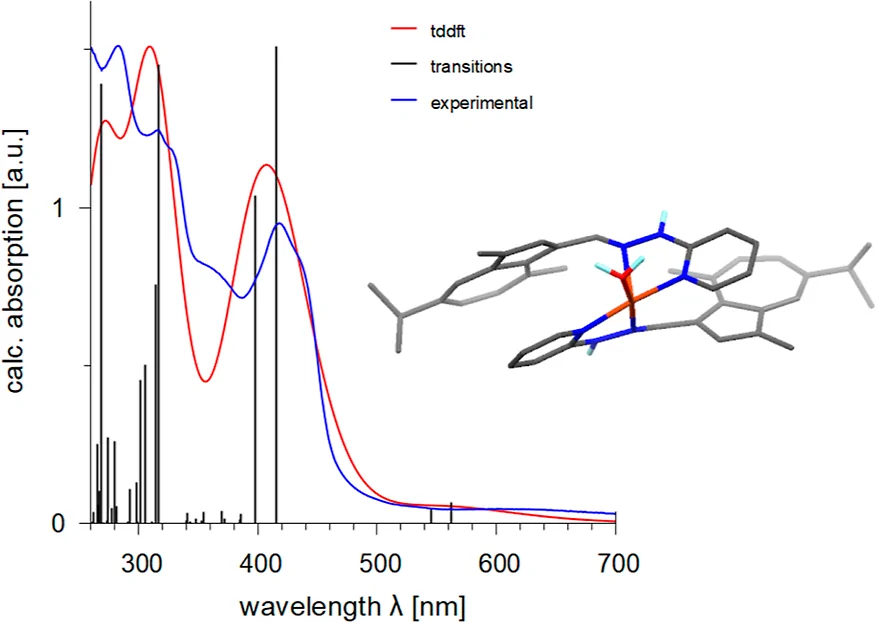
Comparison of the experimental and computed UV–vis spectrum of **Cu­(GAHP550)**_**2**_ with the computed electronic transitions indicated with black vertical lines (similarity factor^87^ of 0.977); DFT predicted structure of **Cu­(GAHP550)**_**2**_.

### 3.7 Logic Gate Applications

In recent years, molecular logic gate applications have also gained increasing attention in chemical sensing studies.^29,61–63,80,88^ The reversible spectrophotometric and colorimetric response of **GAHP550** toward Cu^2+^ in the presence of Na_4_EDTA enabled its use in a molecular logic gate system. In this system, Cu^2+^ and Na_4_EDTA were considered as Input 1 and Input 2, respectively, while the output was defined either as the color change or the absorbance variation at 550 nm (A_550_) (Figure 8).

**Figure 8:**
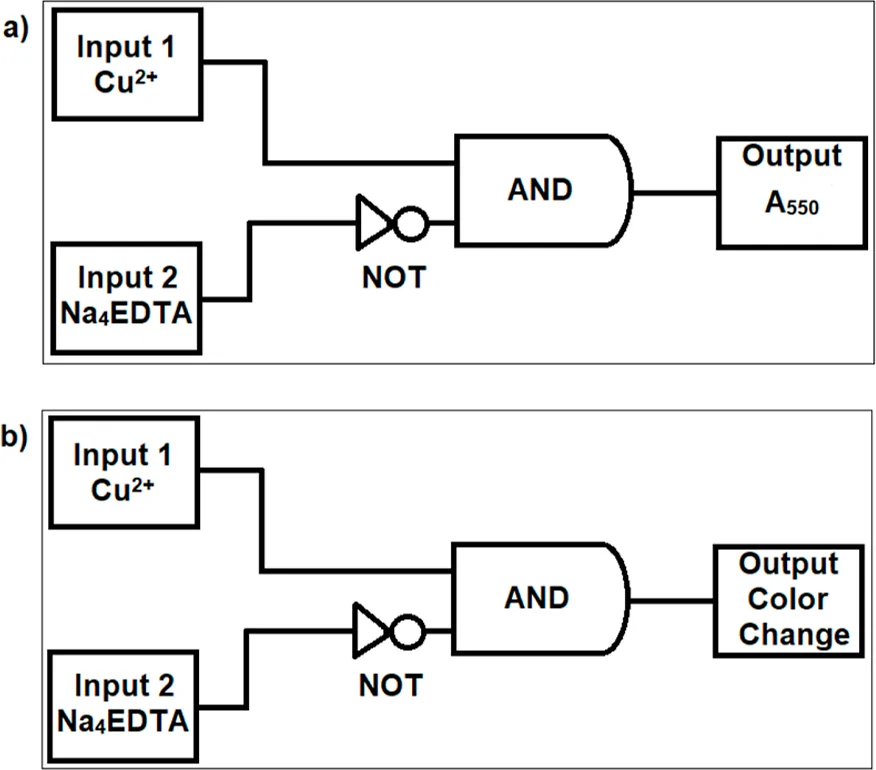
(a) Logic diagram representing the INHIBIT gate behavior based on the absorbance response at 550 nm; (b) corresponding logic circuit illustrating the INHIBIT operation derived from the observed color transition.

When A_550_ was monitored with a threshold value of 0.4, the absence of both Cu^2+^ and Na_4_EDTA resulted in an absorbance below 0.4, corresponding to an output of 0. The presence of Cu^2+^ alone increased the absorbance above 0.4, giving an output of 1. However, when both Cu^2+^ and Na_4_EDTA were present, or when only Na_4_EDTA was present, the absorbance remained below 0.4, giving an output of 0 (Figure S25). Therefore, the system produced an output of 1 only in the Input 1 AND NOT Input 2 condition, while in all other cases the output was 0 (Table 2). This clearly demonstrates that the designed system functions as an INHIBIT logic gate, which represents the combination of AND and NOT operations (Figure 8). Similarly, when the output is defined as the color change, green as 0 and brown as 1, the same logic behavior is observed, further confirming that the **GAHP550** based system successfully mimics an INHIBIT logic gate (Figure S26).

**Table 2:** Truth Table Illustrating the Operational Logic of the INHIBIT Gate

input 1: Cu^2+^	input 2: Na_4_EDTA	output: A_550_	output: color change
0	0	0	0
1	0	1	1
0	1	0	0
1	1	0	0

### 3.8 Real Sample Studies

To evaluate the applicability of **GAHP550** for Cu^2+^ detection in real samples, experiments were conducted using two different bottled drinking water samples and a tap water sample. In this study, the real samples were spiked with Cu­(NO_3_)_2_, and the results were compared with those obtained from Cu^2+^ samples prepared in pure water to calculate the recovery values. As shown in the Table 3, Cu^2+^ additions at concentrations of 0.50, 55.00, and 273.00 μM resulted in excellent recovery values ranging from 83.5% to 104.2%. These findings clearly demonstrate that Cu^2+^ detection using the **GAHP550** probe can be successfully achieved in real water samples.

**Table 3:** Cu^2+^ Recovery Performance and Associated RSD Values Obtained from Real-Sample Studies Using Multiple Bottled Water Sources and Tap Water Matrices

sample	added Cu^2+^ (μM)	found Cu^2+^ (μM)	recovery (%)	RSD (*n* = 3)
tap water	0.50	0.48	95.0	10.2
	55.00	50.08	91.1	0.99
	273.00	227.82	83.5	0.69
drinking water-1	0.50	0.5	100.5	5.47
	55.00	53.17	96.7	1.49
	273.00	232.92	85.3	1.22
drinking water-2	0.50	0.52	104.2	2.63
	55.00	53.61	97.5	1.34
	273.00	234.86	86.0	0.42

### 3.9 Smartphone Based Cu^2+^ Detection

A smartphone application was developed based on the color transition of **GAHP550** from green to brown with increasing Cu^2+^ concentrations. For this purpose, a titration experiment was conducted to capture the color response of **GAHP550** toward Cu^2+^, and photographs were taken throughout the process. Three common pixel points were identified across all images, and their red, green, and blue (RGB) values were calculated using a custom Python code that evaluated all possible variations. The analysis revealed that the red/green (R/G) ratio increased linearly with the rising Cu^2+^ concentration. A linear calibration curve was then constructed, with Cu^2+^ concentration plotted on the *X*-axis and the R/G ratio on the *Y*-axis (Figure 9).

**Figure 9:**
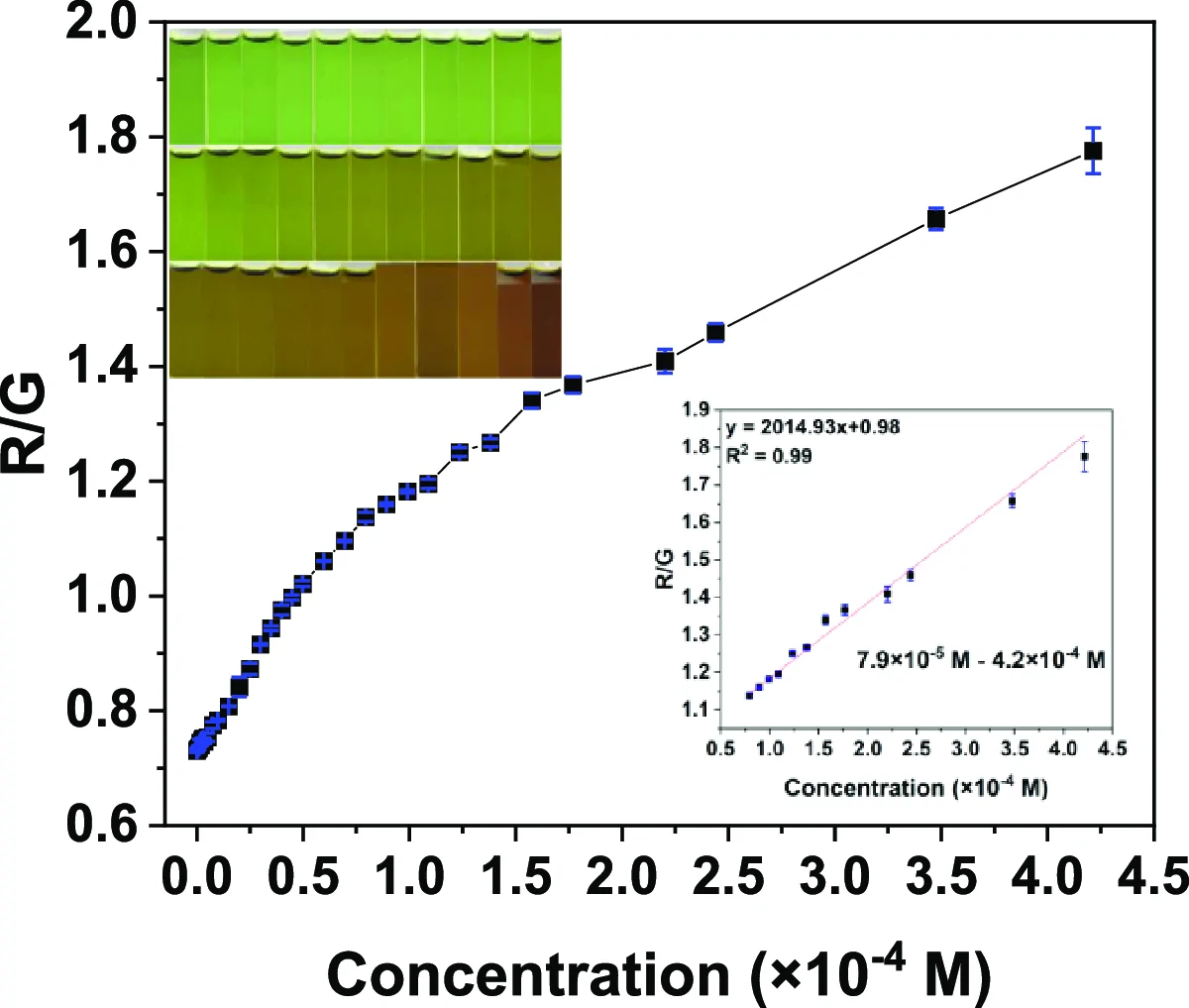
A calibration plot was generated by correlating increasing Cu^2+^ concentrations (7.9 × 10^–5^–4.2 × 10^–4^ M) with the R/G intensity ratio obtained through the smartphone application. Inset: visual representation of the progressive color transition of **GAHP550** (1.0 × 10^–3^ M) upon exposure to rising Cu^2+^ levels (0–4.5 × 10^–4^ M) under the same conditions. Measurement medium: DMSO/H_2_O (17:3, v/v) with 0.1 M HEPES buffer at pH 7.4. Error bars represent the standard deviation of three independent measurements (*n* = 3).

Using the slope, intercept, and linear range obtained from this calibration curve, an Android mobile application named “**GAHP550** Cu^2+^ Detector” was developed. When a photograph is taken directly via the smartphone, the application automatically selects a defined region, calculates the average R/G value, and determines the Cu^2+^ concentration in both M and ppm units if the value falls within the established linear range. If the value lies outside this range, the application notifies the user accordingly (Figure S27). The application developed in this study provides a more user-friendly operation compared with existing smartphone-based applications reported in the literature. As summarized in Table 1, many of the smartphone applications reported to date require multiple user inputs to obtain the final result. In contrast, with the “**GAHP550** Cu^2+^ Detector” application, color selection from the captured image, matching of the obtained response with the calibration curve, and determination of the corresponding Cu^2+^concentration are performed fully automatically. This automated workflow minimizes user intervention and offers clear advantages in terms of user experience, practicality, and ease of operation.

The ability to perform accurate Cu^2+^ detection using only a smartphone and the **GAHP550** probe highlights the strong potential of this system for practical and field-based analytical applications.

### 3.10 Deployment on Cotton Buds

To evaluate the practical applicability of the probe, a cotton bud-based colorimetric assay was also developed. Probe-coated cotton buds were prepared by immersing them in a 1.0 × 10^–3^ M **GAHP550** solution in DMSO/pH 7.4 HEPES (0.1 M) (17:3, v/v) and allowing them to dry at room temperature. The dried cotton buds were then immersed in Cu^2+^ solutions containing different equivalents of the metal ion and subsequently photographed.

As shown in Figure 10, the initially green probe-coated cotton buds exhibited a distinct color transition from yellow to brown with increasing Cu^2+^ concentration. To further assess the selectivity of this platform, the probe-coated cotton buds were exposed to various competing ions under identical conditions. As illustrated in Figure 11, no noticeable color change was observed for any of the competing species, whereas a clear color transition occurred exclusively in the presence of Cu^2+^.

**Figure 10:**
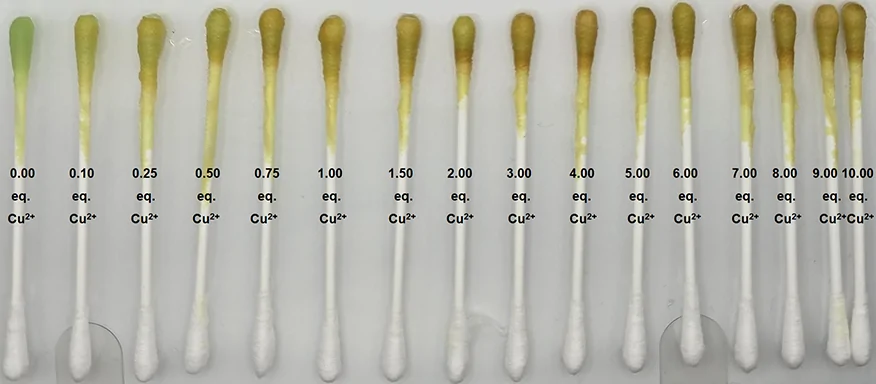
Colorimetric response of **GAHP550**-coated cotton buds after immersion for 30 s in Cu^2+^ solutions containing 0–10 equiv of Cu^2+^. The cotton buds were preimpregnated with 1.0 × 10^–3^ M **GAHP550** in DMSO/pH 7.4 HEPES (0.1 M) (17:3, v/v) and dried at room temperature prior to use.

**Figure 11:**
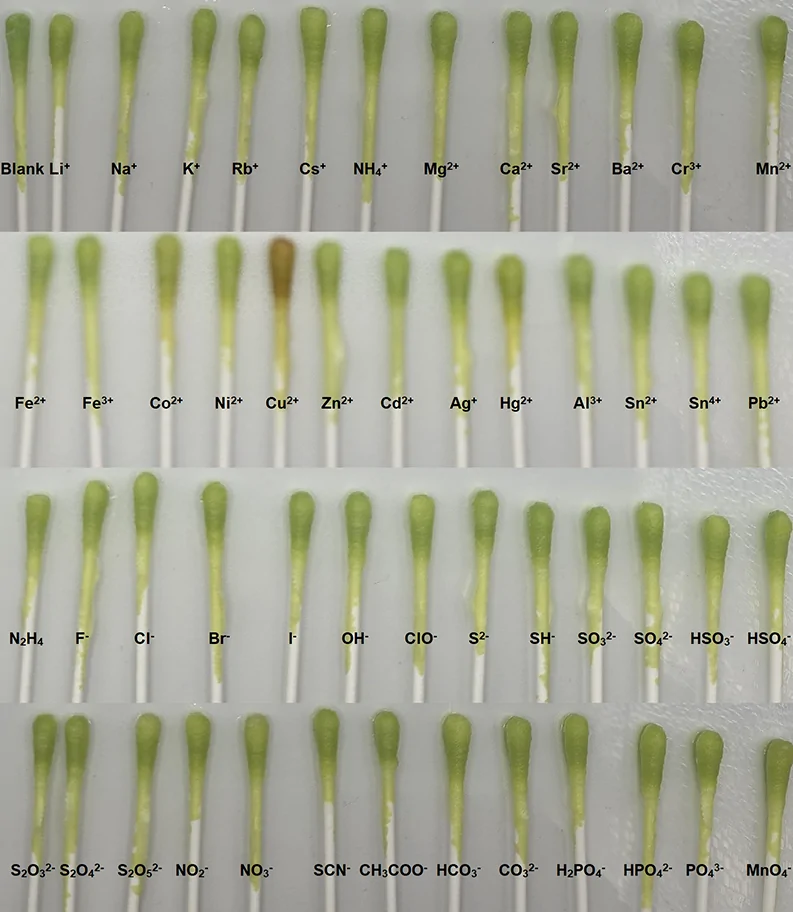
Colorimetric selectivity of **GAHP550**-coated cotton buds toward Cu^2+^ in the presence of competing ions (5.0 equiv). The cotton buds were preimpregnated with 1.0 × 10^–3^ M **GAHP550** in DMSO/pH 7.4 HEPES (0.1 M) (17:3, v/v), dried at room temperature, and immersed in each ion solution for 30 s prior to photography.

These results demonstrate that **GAHP550**-impregnated cotton buds provide a simple, highly selective, and sensitive platform for the visual detection of Cu^2+^, highlighting their potential for practical on-site applications.

## 4 Conclusion

Our guaiazulene-based probe **GAHP550** provides a rapid, reversible, and highly selective platform for Cu^2+^ detection, achieving an ultralow detection limit of 9.45 × 10^–7^ M (55.4 ppb), well below WHO and EPA thresholds. Its 1:2 binding stoichiometry and high binding constant (1.21 × 10^6^ M^–2^) ensure strong affinity and reproducible sensing. The probe exhibits INHIBIT-type molecular logic gate functionality, enabling chemical information processing through analyte-triggered optical outputs. In real sample analysis, **GAHP550** accurately quantified Cu^2+^ in tap and bottled water with excellent recovery values ranging from 83.5–104.2%, demonstrating robustness in practical matrices. A dedicated smartphone application was developed to quantify Cu^2+^ concentrations in real-time using RGB analysis of the colorimetric transition, enabling precise on-site monitoring and mobile-based detection. By combining ultrahigh sensitivity, instantaneous colorimetric response, reversibility, logic-gate operation, and mobile-readout capability, **GAHP550** represents a versatile tool for both laboratory and field-based Cu^2+^ monitoring, highlighting the potential of portable chemical sensors in smart practical analytical applications.

## Supplementary Material







## Data Availability

All data included as part of the manuscript and its Supporting Information.

## Notes

**GAHP550** Cu^2+^ Detector Android application (ZIP)
Experimental procedures, synthesis and characterization of 3-formylguaiazulene and **GAHP550**, UV–Vis optimization studies (concentration, pH, and solvent composition), ^1^H, ^13^C, COSY, NOESY, HSQC, HMBC NMR, IR, and HRMS spectra, NMR titration and HRMS analysis, stability, Job’s plot, Benesi–Hildebrand analysis, interference, response time, and reversibility studies, molecular logic gate experiments, mobile application interface, single-crystal X-ray diffraction data, DFT calculations and molecular modeling (PDF)
Single-crystal X-ray crystallographic data (CIF)

## #

EMAIL: mehmetkaplan@ankara.edu.tr
