# Supplementary material for: Guaiazulene-Based 2‑Hydrazinylpyridine Probe (GAHP550) for Ultralow, Reversible Cu2+ Detection and Molecular Logic Gate Applications
Source: ACS Omega. 2026 Aug 25;11(35):52968–79. doi: 10.1021/acsomega.6c05174 (PMC13563978; doi:10.1021/acsomega.6c05174)
Supplement: Supplementary file 1 [file ao6c05174_si_002.pdf]

Supporting Information

Guaiazulene-Based 2-Hydrazinylpyridine Probe (GAHP550) for Ultralow, Reversible Cu^{2+} Detection and Molecular Logic Gate Applications

Mehmet Kaplan[a b], Gabriele Kociok-Köhn[c], G. Dan Pantoş[a]*, Simon E. Lewis[a]*, and Tony D. James[a d]*

[a]Department of Chemistry, University of Bath, Bath BA2 7AY, UK

[b]Ankara University, Faculty of Science, Department of Chemistry, 06100, Ankara, Turkiye

[c]Physical Structure Characterization, University of Bath, Claverton Down, Bath BA2 7AY, UK

[d]School of Chemistry and Chemical Engineering, Henan Normal University, Xinxiang 453007, PR China

Author (Mehmet Kaplan): e-mail address: mehmetkaplan@ankara.edu.tr

Author (Gabriele Kociok-Köhn): Tel.: +44 (0)1225 386520

e-mail address: chsgk@bath.ac.uk

*Corresponding author (G. Dan Pantoş). Tel.: +44 (0) 1225 384376

e-mail address: G.D.Pantos@bath.ac.uk

*Corresponding author (Simon E. Lewis). Tel.: +44 (0) 1225 386568

e-mail address: S.E.Lewis@bath.ac.uk

*Corresponding author (Tony D. James). Tel.: +44 (0) 1225 383810

e-mail address: T.D.James@bath.ac.uk

LIST OF FIGURES

Figure S1. Synthesis of 3-formylguaiazulene. 5

Figure S2. (a) UV–Vis absorption spectra of **GAHP550** (1.0×10^{-3} M) in DMSO:H_2O (17:3, v/v) containing 0.1 M HEPES buffer at pH 7.4 over the concentration range of 1.0×10^{-4}–2.5×10^{-3} M; (b) corresponding linear calibration plot at 640 nm. Error bars represent the standard deviation of three independent measurements (n = 3). 6

Figure S3. pH-dependent response of **GAHP550** (1.0×10^{-3} M) and its Cu^{2+} complex in different buffer systems. Medium: DMSO:H_2O (17:3, v/v), pH adjusted accordingly. Error bars represent the standard deviation of three independent measurements (n = 3). 7

Figure S4. Effect of increasing water content on the response of **GAHP550** (1.0×10^{-3} M) and its Cu^{2+} complex in DMSO:H_2O with 0.1 M HEPES buffer at pH 7.4. Error bars represent the standard deviation of three independent measurements (n = 3). 8

Figure S5. ^{1}H NMR spectrum ($CDCl_3$, 400 MHz) of 3-formylguiazulene. 9

Figure S6. ^{13}C NMR spectrum ($CDCl_3$, 101 MHz) of 3-formylguiazulene. 10

Figure S7. HRMS (ESI+) result of 3-formylguiazulene m/z: $[(C_{16}H_8O)+H]^+$ theoretical: 227.1430, found: 227.1429. 11

Figure S8. ^{1}H NMR spectrum (DMSO-d_6, 400 MHz) of **GAHP550**. 12

Figure S9. ^{13}C NMR spectrum (DMSO-d_6, 101 MHz) of **GAHP550.** 13

Figure S10. COSY NMR spectrum (DMSO-d_6, 400 MHz) of **GAHP550.** 14

Figure S11. NOESY NMR spectrum (DMSO-d_6, 400 MHz) of **GAHP550.** 15

Figure S12. HSQC NMR spectrum (DMSO-d_6, 101 MHz) of **GAHP550.** 16

Figure S13. HMBC NMR spectrum (DMSO-d_6, 101 MHz) of **GAHP550.** 17

Figure S14. IR spectrum of **GAHP550**. 18

Figure S15. HRMS (ESI+) result of **GAHP550** m/z: $[(C_{21}H_{23}N_3)+H]^+$ theoretical: 318.1965, found: 318.1964. 19

Figure S16. Partial ^{1}H NMR (DMSO-d_6, 101 MHz) of **GAHP550** (top, blue) and **Cu(GAHP550)$_2$** (bottom, brown). 20

Figure S17. Overlay of NOESY (green) / COSY (brown) spectra (DMSO-d_6, 101 MHz) of **Cu(GAHP550)$_2$.** 21

Figure S18. Stability evaluation of **GAHP550** (1.0×10^{-3} M, DMSO:H_2O 17:3, v/v, 0.1 M HEPES, pH 7.4) over a 100-day period. Inset: zoomed-in view of the 0-10 day stability

profile. Error bars represent the standard deviation of three independent measurements (n = 3).. 22
Figure S19. Job's Plot (1.0×10^{-4} M). Error bars represent the standard deviation of three independent measurements (n = 3). 23
Figure S20. Benesi-Hildebrand linearization. 24
Figure S21. Interference study of **GAHP550** (1.0×10^{-3} M) upon addition of Cu^{2+} in the presence of varying equivalents (2.5–25.0 equiv.) of competing ions (Li^{+}, Na^{+}, K^{+}, Rb^{+}, Cs^{+}, NH_4^{+}, Mg^{2+}, Ca^{2+}, Sr^{2+}, Ba^{2+}, Cr^{3+}, Mn^{2+}, Fe^{2+}, Fe^{3+}, Co^{2+}, Ni^{2+}, Zn^{2+}, Cd^{2+}, Ag^{+}, Hg^{2+}, Al^{3+}, Sn^{2+}, Sn^{4+}, Pb^{2+}, N_2H_4, F^{-}, Cl^{-}, Br^{-}, I^{-}, OH^{-}, ClO^{-}, S^{2-}, SH^{-}, SO_3^{2-}, SO_4^{2-}, HSO_3^{-}, HSO_4^{-}, $S_2O_3^{2-}$, $S_2O_4^{2-}$, $S_2O_5^{2-}$, NO_2^{-}, NO_3^{-}, SCN^{-}, CH_3COO^{-}, HCO_3^{-}, CO_3^{2-}, $H_2PO_4^{-}$, HPO_4^{2-}, PO_4^{3-}, MnO_4^{-}). Medium: DMSO:H_2O (17:3, v/v, 0.1 M HEPES, pH 7.4). *Hg^{2+} ions were masked with fourfold $Na_2S_2O_3$. Error bars represent the standard deviation of three independent measurements (n = 3). 25
Figure S22. Time-dependent response of **GAHP550** (1.0×10^{-3} M) toward Cu^{2+} over 0–600 s. Inset: detailed view of the initial 0–60 s. Medium: DMSO:H_2O (17:3, v/v, 0.1 M HEPES, pH 7.4). Error bars represent the standard deviation of three independent measurements (n = 3). 26
Figure S23. Reversibility of **GAHP550** (1.0×10^{-3} M) upon sequential addition of Cu^{2+} and Na_4EDTA. Medium: DMSO:H_2O (17:3, v/v, 0.1 M HEPES, pH 7.4). Error bars represent the standard deviation of three independent measurements (n = 3). 27
Figure S24. HRMS (ESI+) result of **Cu(GAHP550)$_2$** complex. 28
Figure S25. The graph of output signals at A_{550} nm for different inputs. Error bars represent the standard deviation of three independent measurements (n = 3). 29
Figure S26. Output signals depicting the color change under different input conditions. Medium: DMSO:H_2O (17:3, v/v, 0.1 M HEPES, pH 7.4). 30
Figure S27. User interface of the "**GAHP550** Cu^{2+} Detector" mobile application.......... 31
Figure S28. ORTEP diagram of **GAHP550.** Ellipsoids are drawn at 50% probability**.** .. 32
Figure S29. Geometry optimised structures of **Cu(GAHP550)$_2$**................................... 42

LIST OF TABLES

Table S1. Crystal data and structure refinement for **GAHP550**.............................. 33

Table S2. Atomic coordinates ($x10^4$) and equivalent isotropic displacement parameters ($Å^2x10^3$) for **GAHP550**. 34

Table S3. Bond lengths [Å] for **GAHP550**. 35

Table S4. Bond angles [°] for **GAHP550**. 36

Table S5. Anisotropic displacement parameters ($Å^2x10^3$) for **GAHP550**. 38

Table S6. Hydrogen coordinates ($x10^4$) and isotropic displacement parameters ($Å^2x10^3$) for **GAHP550**. 39

Table S7. Torsion angles [°] for **GAHP550**. 40

Table S8. Hydrogen bonds for **GAHP550** [Å and °]. 41

Table S9. Molecular modelling energy and similarity factors. 43

Synthesis of 3-Formylguaiazulene

According the literature [1], phosphorus oxychloride ($POCl_3$) (5.0 mL, 53.0 mmol) was added to a guaiazulene solution (5.0 g, 25.0 mmol) in 20 mL of DMF at 0°C under a nitrogen atmosphere. The mixture was stirred in an ice bath for 1.5 hours and then carefully neutralized with a 2.0 M aqueous KOH solution. The resulting purple solution was extracted with CH_2Cl_2 (3×50.0 mL), and the combined organic layers were dried over $MgSO_4$ and concentrated under reduced pressure. The crude product was purified by column chromatography (Hexane/EtOAc, 6:1), yielding 3-formylguaiazulene as a purple solid (4.5 g, 79.5% yield) (Figure S1). **R_f:** 0.60 (SiO_2, 3:1 Pentane/EtOAc); **^{1}H NMR ($CDCl_3$, 400 MHz): δ =** 10.64 (s, 1H, H-16), 8.29 (d, *J* = 2.1 Hz, 1H, H-14), 8.23 (s, 1H, H-3), 7.59 (dd, *J* = 10.8, 2.2 Hz, 1H, H-9), 7.43 (d, *J* = 10.8 Hz, 1H, H-8), 3.16 (m, 4H, H-7, H-11), 2.59 (s, 3H, H-2), 1.39 (d, *J* = 6.9 Hz, 6H, H-12, H-13) ppm; **^{13}C NMR ($CDCl_3$, 101 MHz): δ =** 186.4 (C-16), 147.1 (C-6), 146.7 (C-10), 143.7 (C-3), 139.5 (C-15), 139.3 (C-5), 136.2 (C-9), 135.5 (C-14), 132.6 (C-8), 127.5 (C-1), 127.2 (C-4), 38.3 (C-11), 30.2 (C-7), 24.6 (C-12, C-13), 13.1 (C-2) ppm; **HRMS (ESI+) m/z:** $[(C_{16}H_8O)+H]^+$ **theoretical:** 227.1430, **found:** 227.1429.

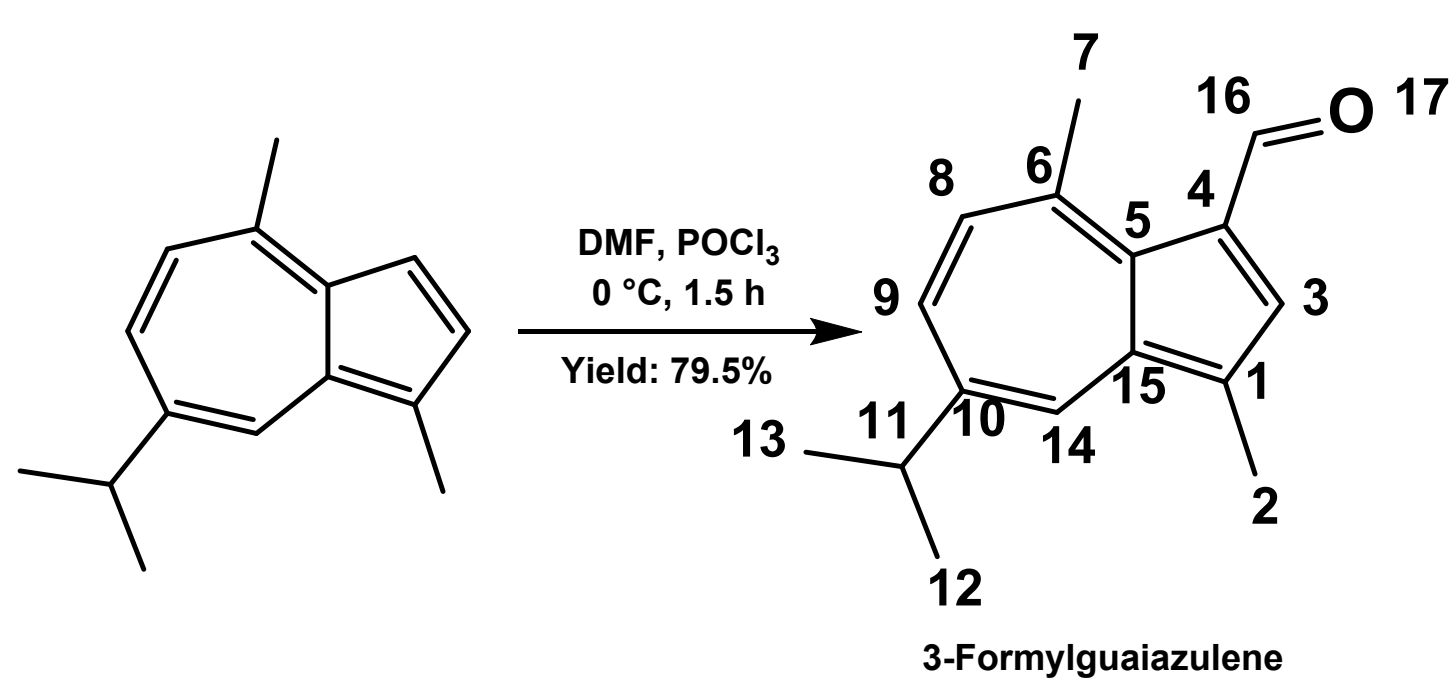

Figure S1. Synthesis of 3-formylguaiazulene.

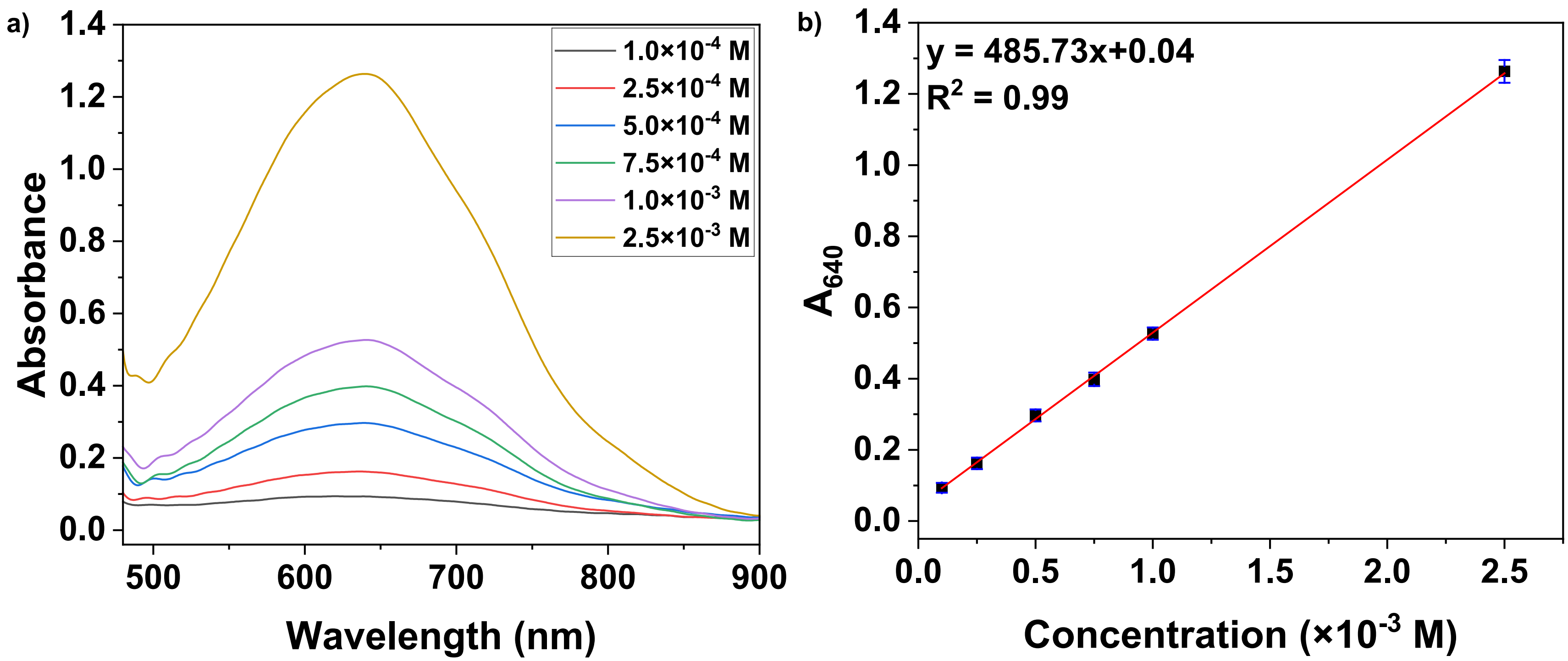

Figure S2. (a) UV–Vis absorption spectra of **GAHP550** (1.0×10^{-3} M) in DMSO:H_2O (17:3, v/v) containing 0.1 M HEPES buffer at pH 7.4 over the concentration range of 1.0×10^{-4}–2.5×10^{-3} M; (b) corresponding linear calibration plot at 640 nm. Error bars represent the standard deviation of three independent measurements (n = 3).

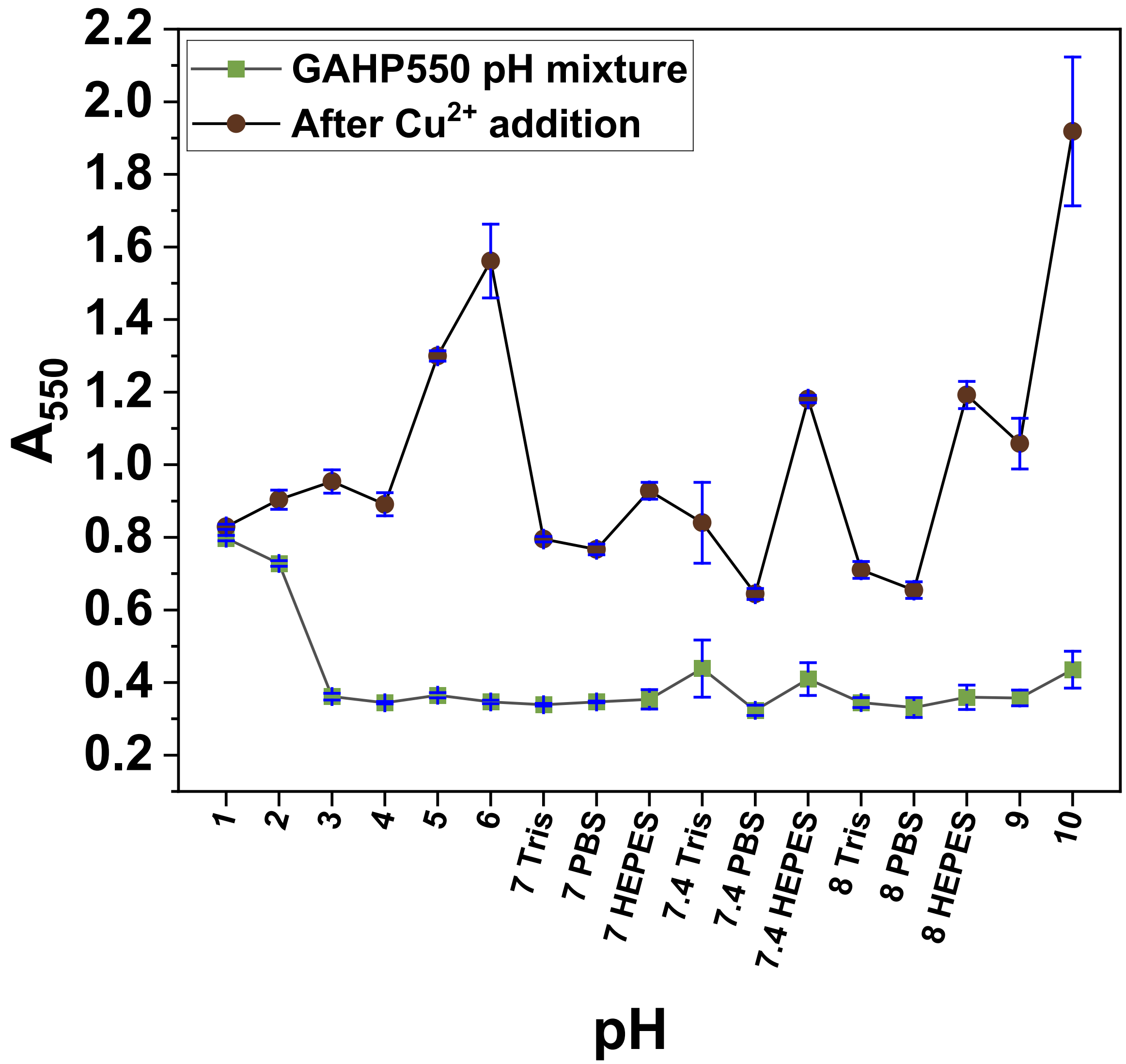

Figure S3. pH-dependent response of **GAHP550** (1.0×10^{-3} M) and its Cu^{2+} complex in different buffer systems. Medium: DMSO:H_2O (17:3, v/v), pH adjusted accordingly. Error bars represent the standard deviation of three independent measurements (n = 3).

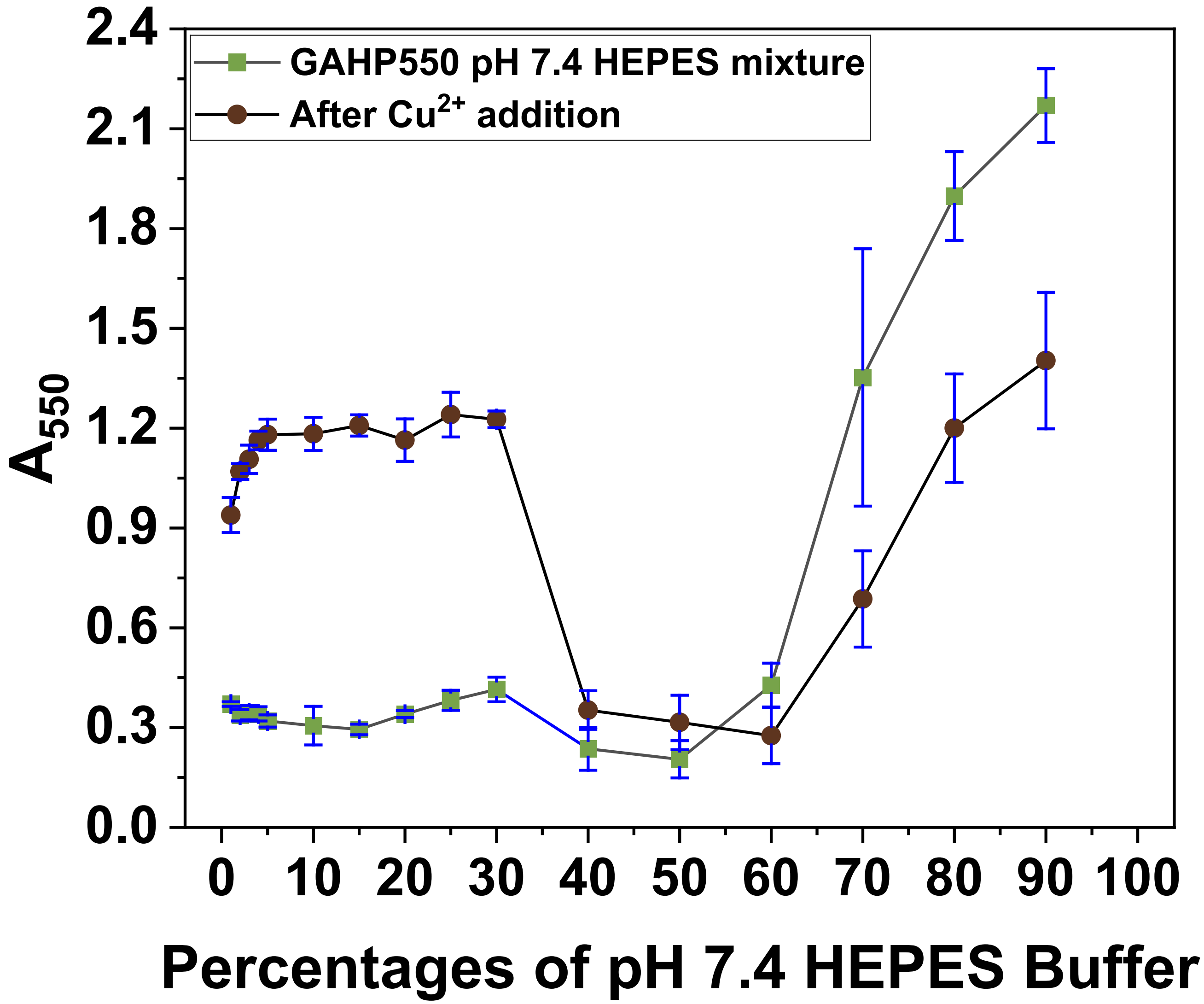

Figure S4. Effect of increasing water content on the response of **GAHP550** ($1.0{\times}10^{-3}$ M) and its Cu^{2+} complex in DMSO:H_2O with 0.1 M HEPES buffer at pH 7.4. Error bars represent the standard deviation of three independent measurements (n = 3).

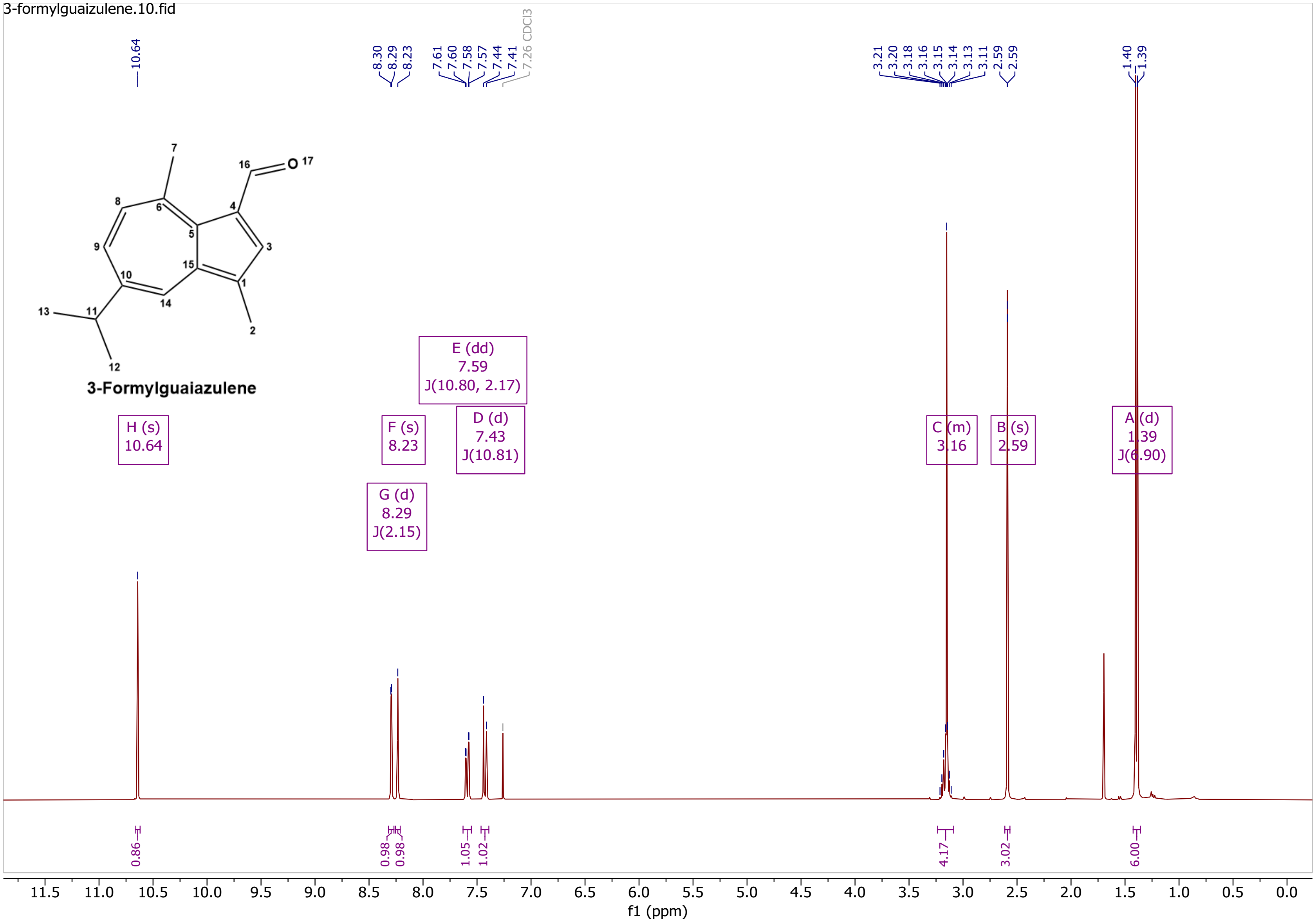

Figure S5. ^{1}H NMR spectrum ($CDCl_3$, 400 MHz) of 3-formylguiazulene.

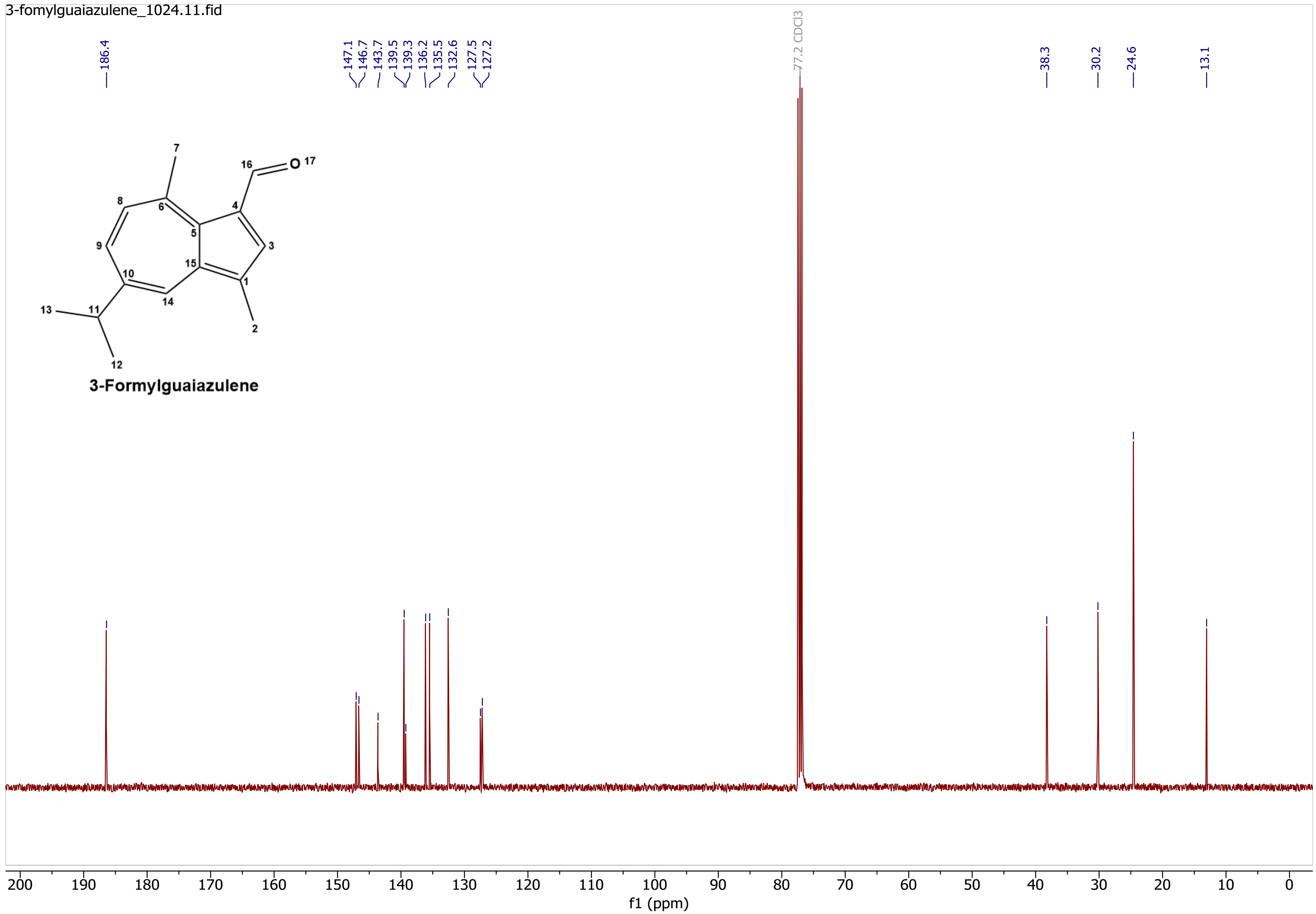

Figure S6. ^{13}C NMR spectrum ($CDCl_3$, 101 MHz) of 3-formylguiazulene.

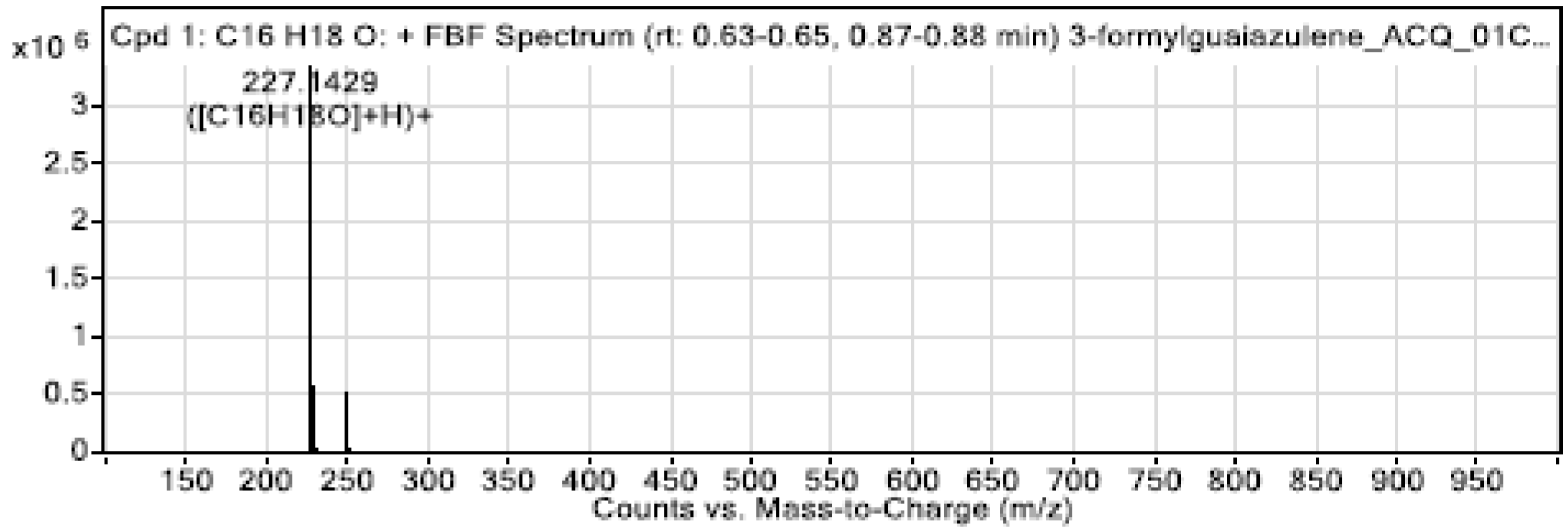

Peak List

m/z	z	Abund	Formula	Ion
227.1429	1	3345488	C16H18O	(M+H)+
228.1463	1	550831.13	C16H18O	(M+H)+
229.149	1	57782.45	C16H18O	(M+H)+
230.1511	1	4912.21	C16H18O	(M+H)+
249.1251	1	499306.91	C16H18O	(M+Na)+
250.1281	1	77403.38	C16H18O	(M+Na)+
251.1312	1	8878.93	C16H18O	(M+Na)+

Figure S7. HRMS (ESI+) result of 3-formylguiazulene m/z: $[(C_{16}H_8O)+H]^+$ theoretical: 227.1430, found: 227.1429.

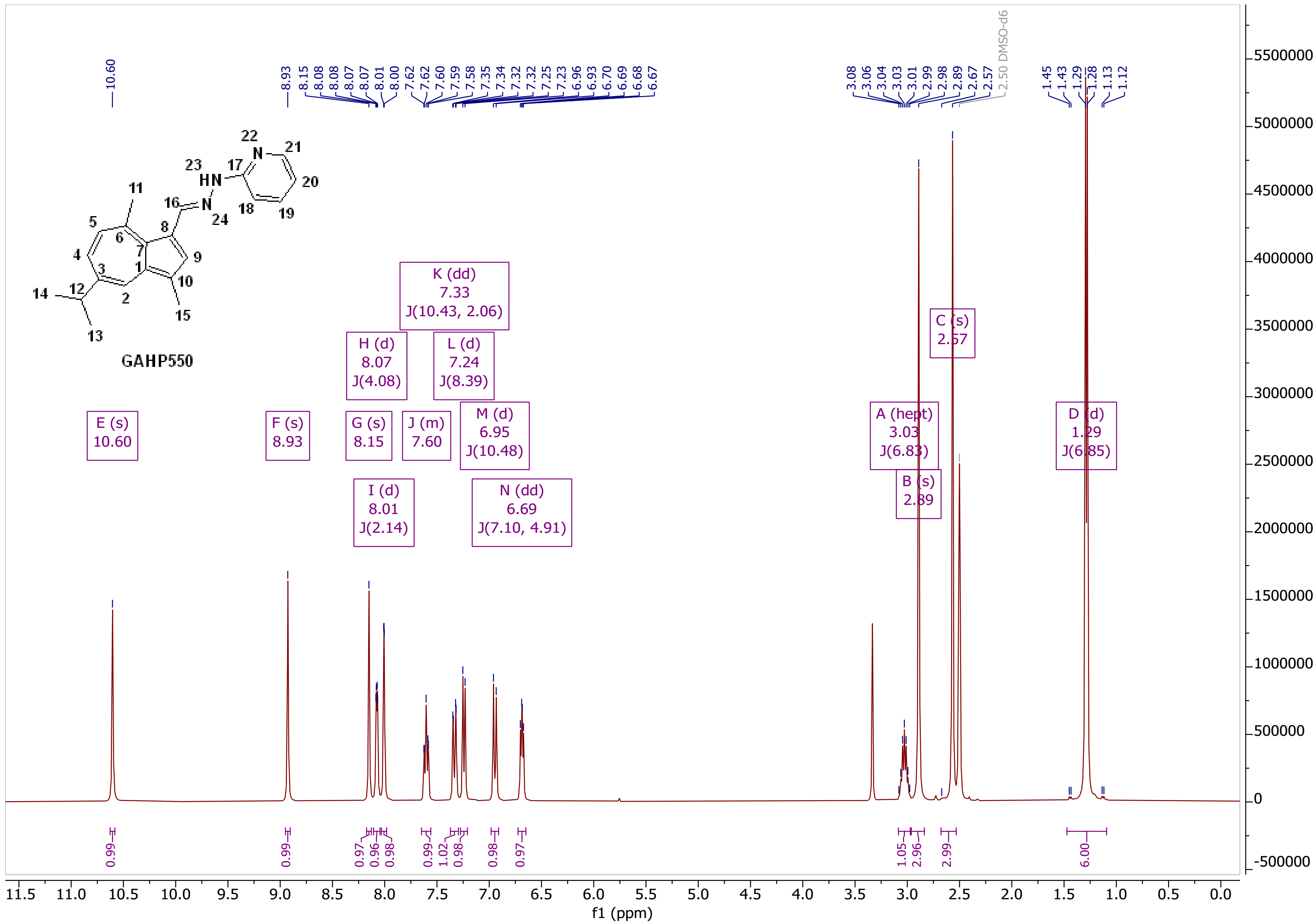

Figure S8. ^{1}H NMR spectrum (DMSO-d_6, 400 MHz) of **GAHP550**.

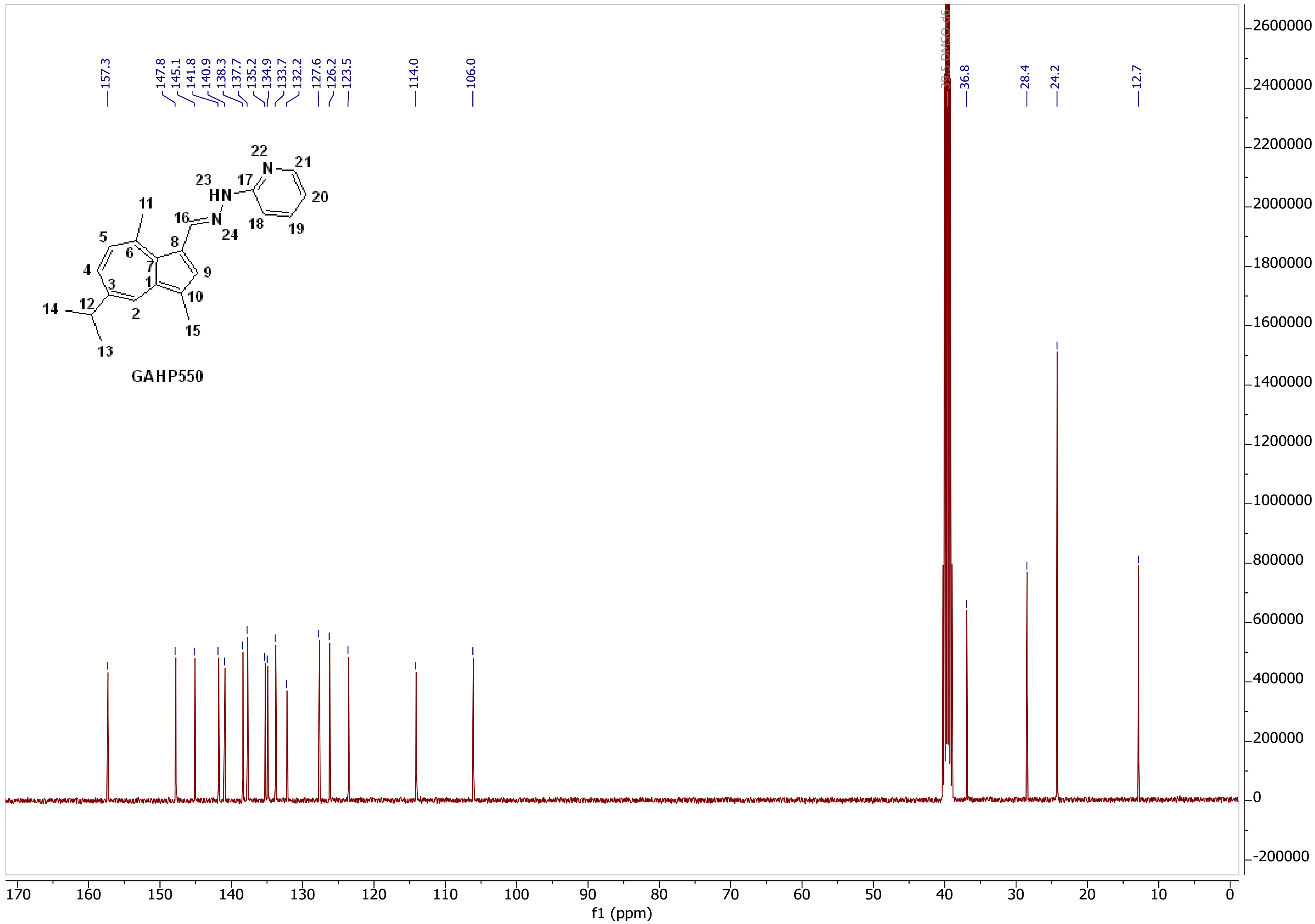

Figure S9. ^{13}C NMR spectrum (DMSO-d_6, 101 MHz) of **GAHP550.**

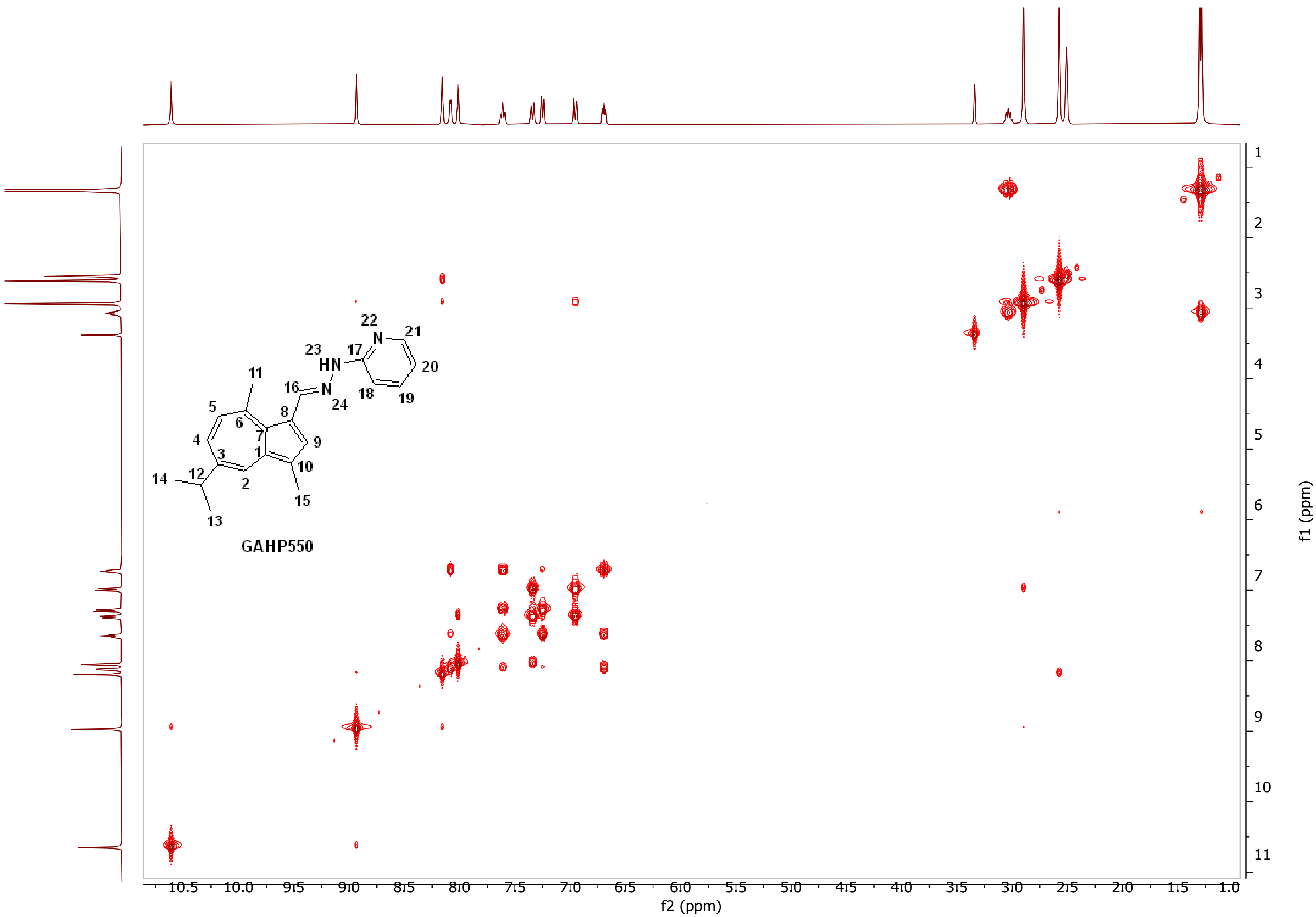

Figure S10. COSY NMR spectrum (DMSO-d_6, 400 MHz) of **GAHP550.**

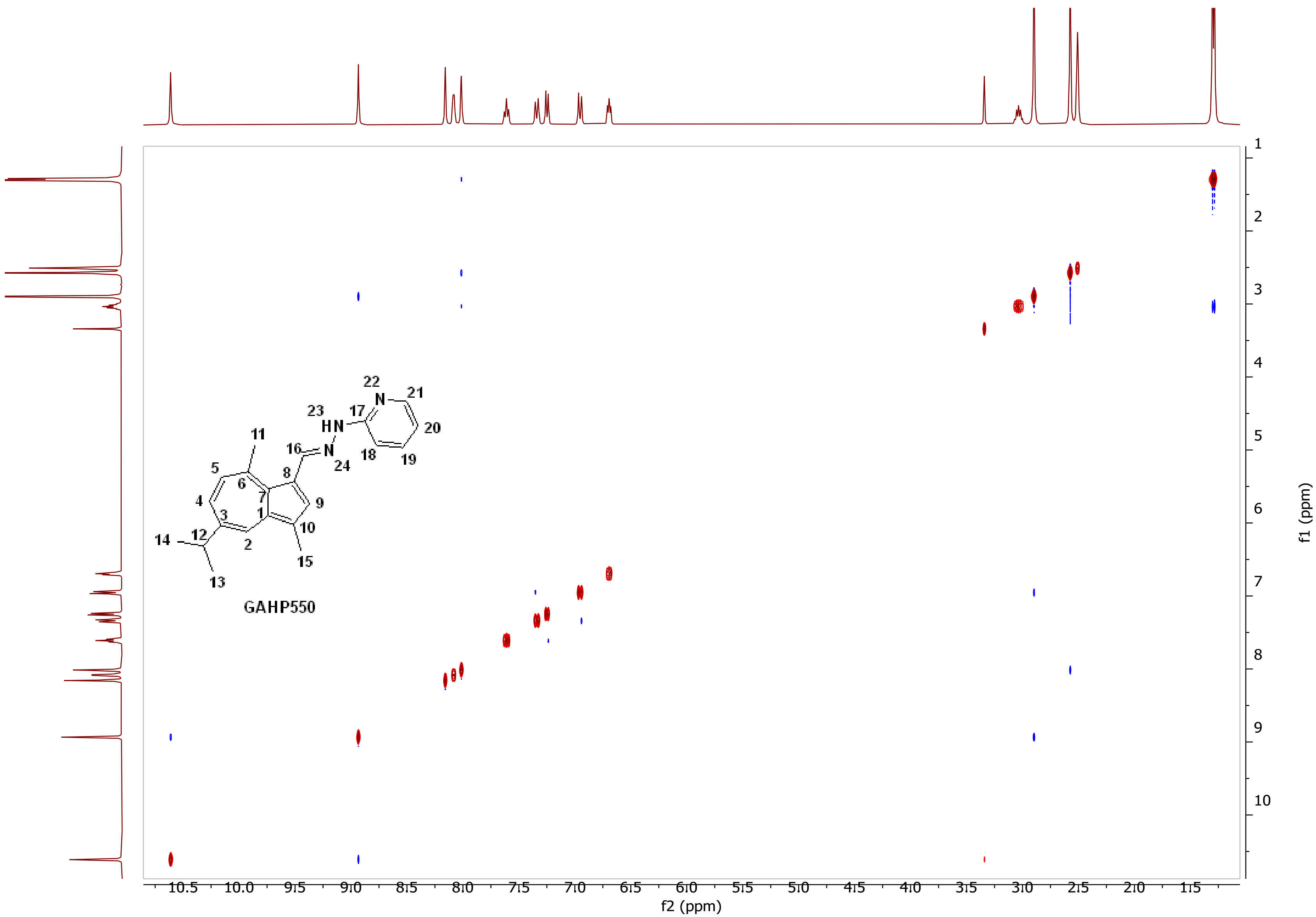

Figure S11. NOESY NMR spectrum (DMSO-d_6, 400 MHz) of **GAHP550.**

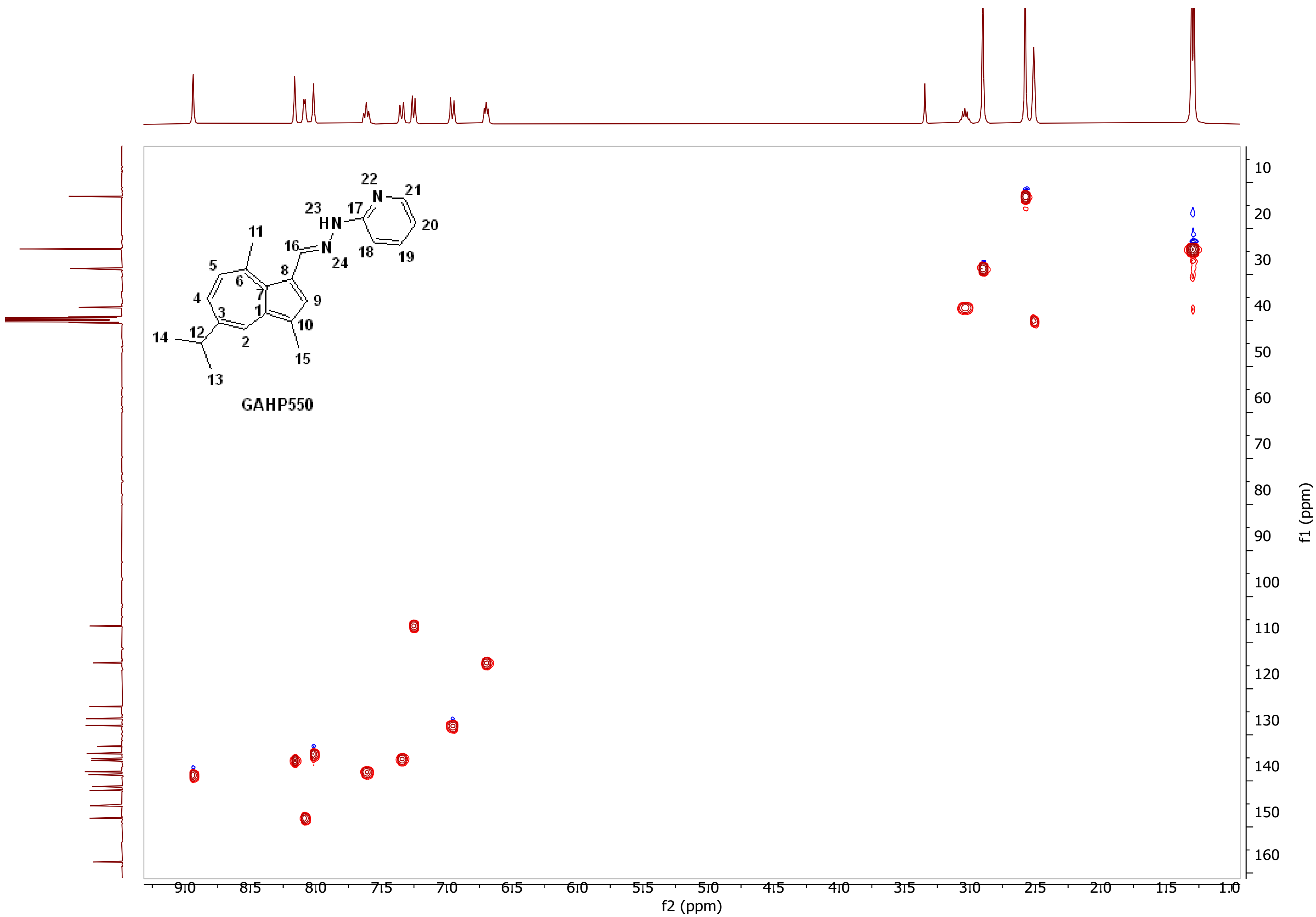

Figure S12. HSQC NMR spectrum (DMSO-d_6, 101 MHz) of **GAHP550.**

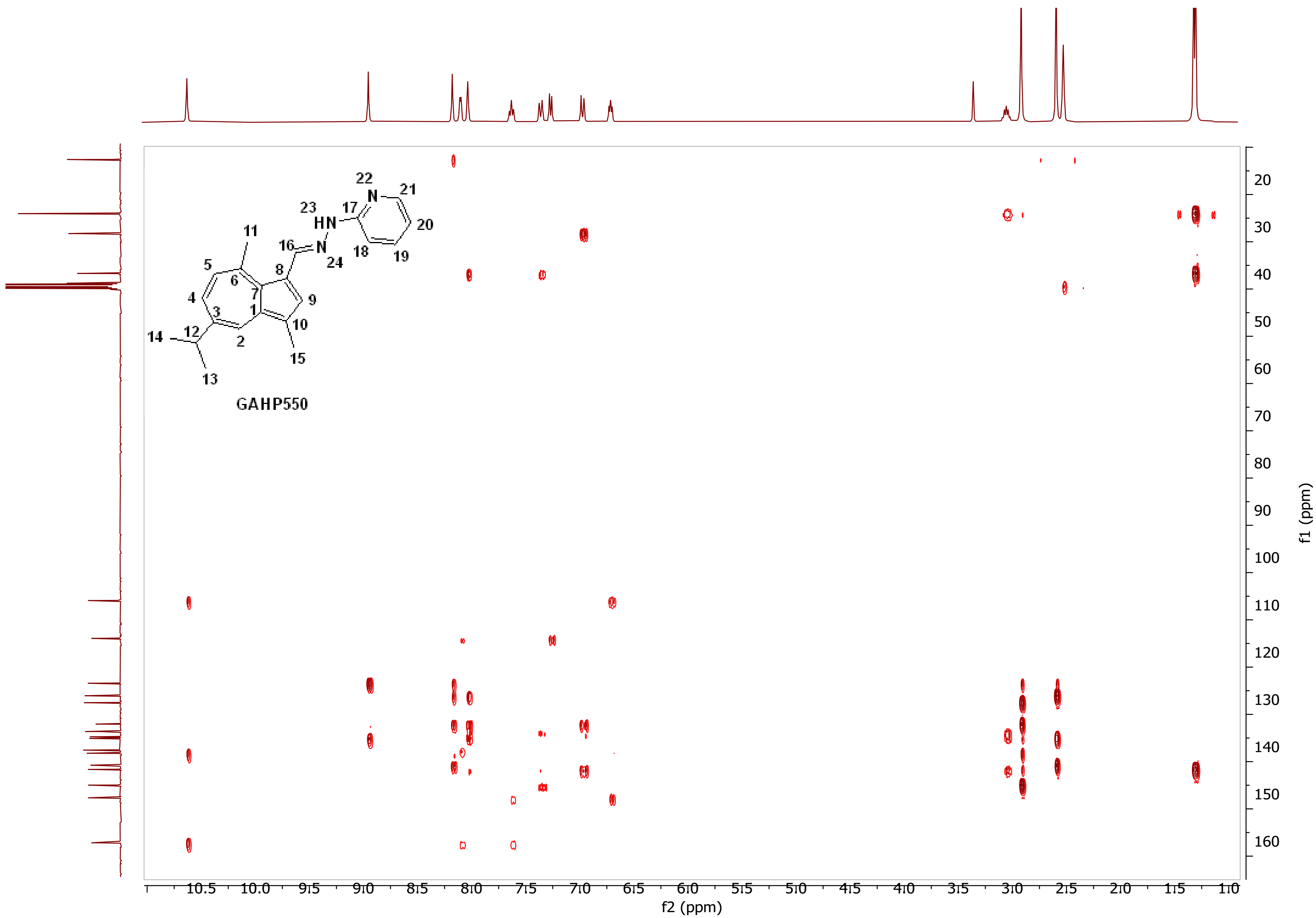

Figure S13. HMBC NMR spectrum (DMSO-d_6, 101 MHz) of **GAHP550.**

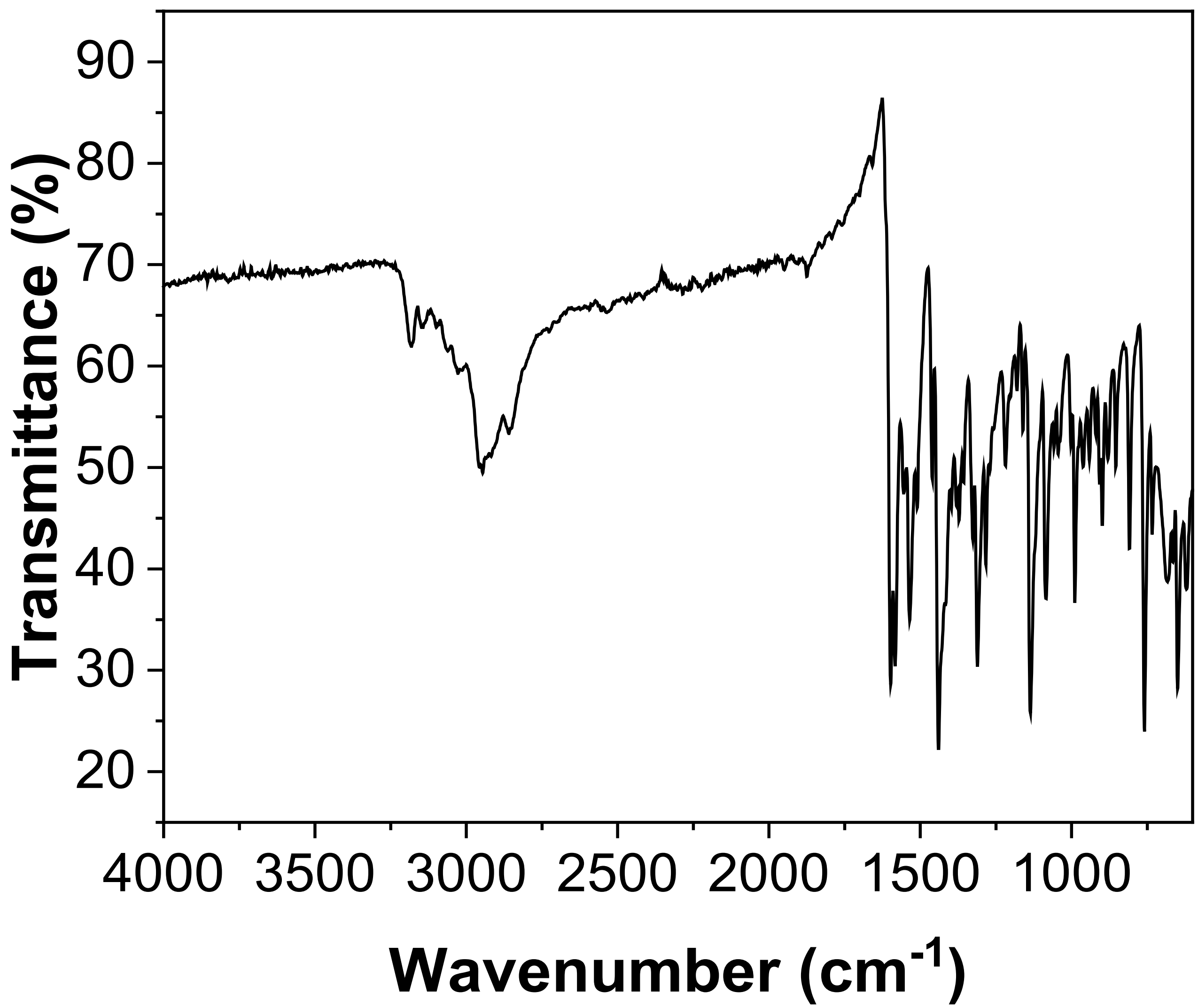

Figure S14. IR spectrum of **GAHP550**.

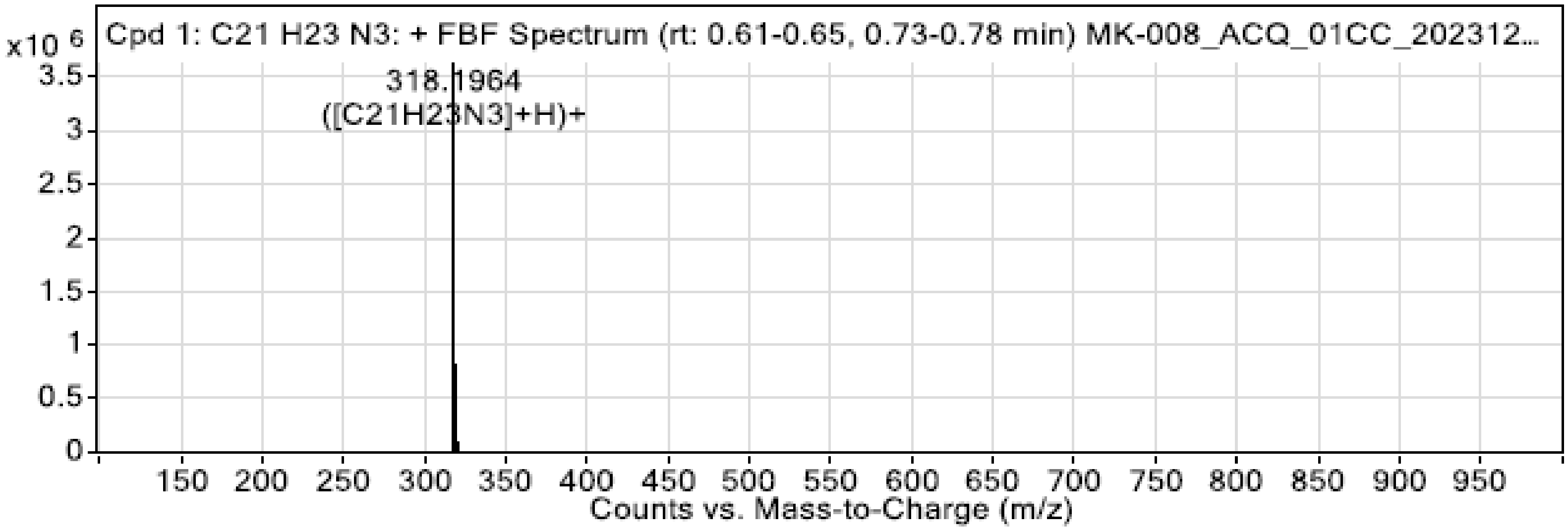

Peak List

m/z	z	Abund	Formula	Ion
318.1964	1	3616930.25	C21H23N3	(M+H)+
319.1998	1	823068.06	C21H23N3	(M+H)+
320.2031	1	87652.23	C21H23N3	(M+H)+
321.2056	1	8947.9	C21H23N3	(M+H)+
340.1781	1	2791.88	C21H23N3	(M+Na)+

Figure S15. HRMS (ESI+) result of **GAHP550** m/z: $[(C_{21}H_{23}N_3)+H]^+$ theoretical: 318.1965, found: 318.1964.

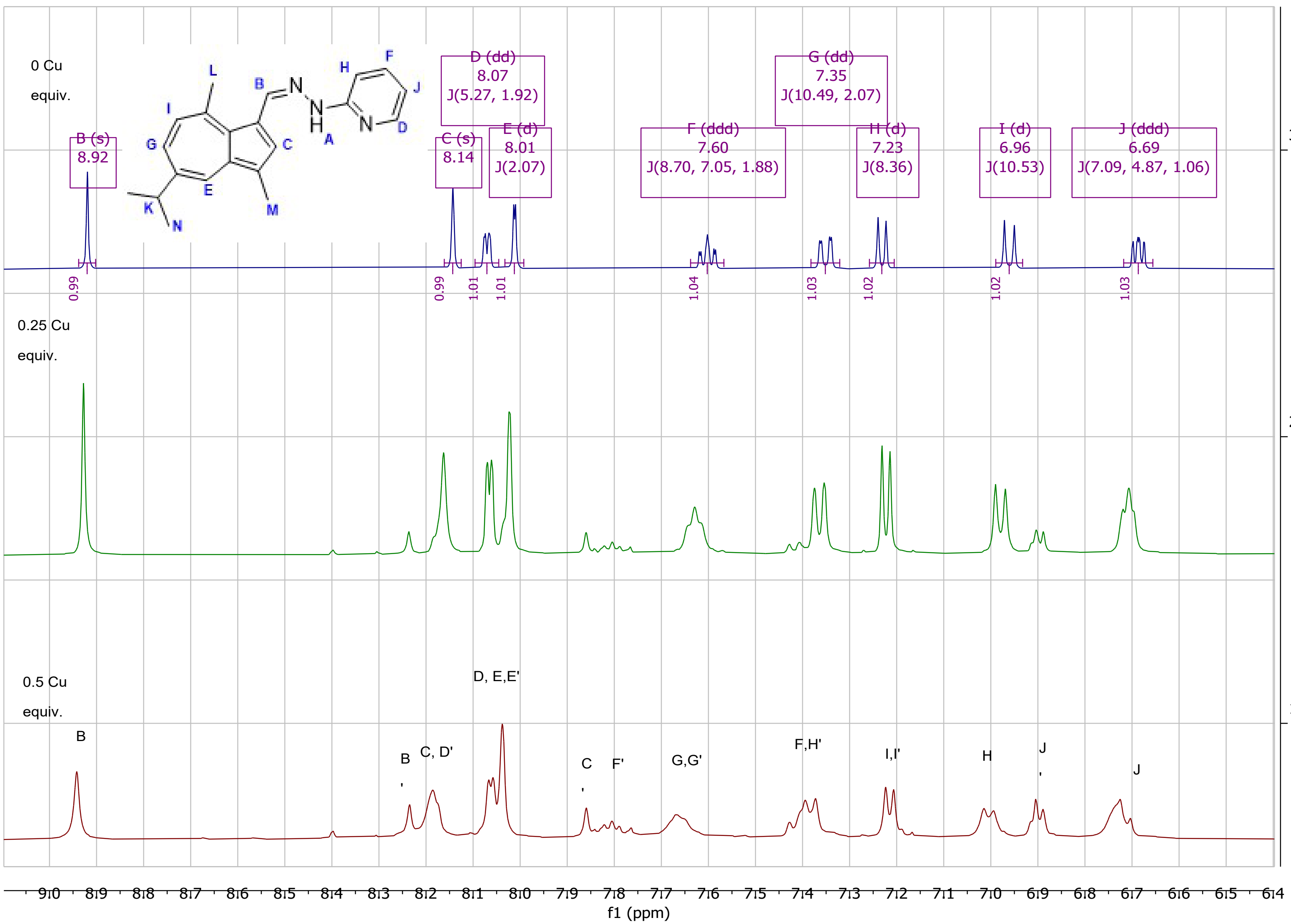

Figure S16. ^1H NMR (DMSO-d_6, 400 MHz) of **GAHP550** with 0 (top, blue), 0.25 (middle, green) and 0.5 (bottom, brown) equivalents of Cu^{2+}.

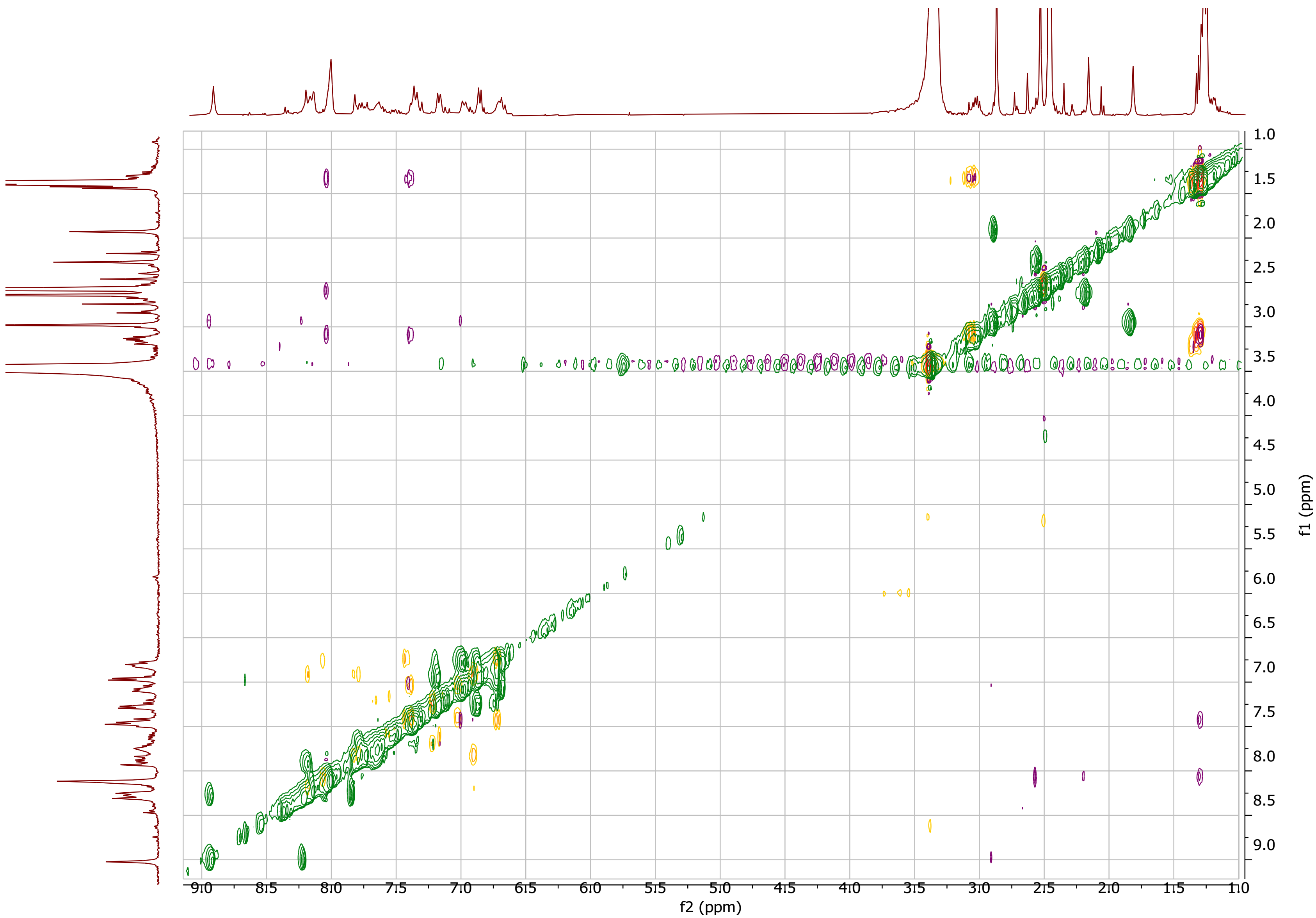

Figure S17. Overlay of COSY (yellow) / NOESY (green-purple) spectra (DMSO-d_6) of **Cu(GAHP550)$_2$.**

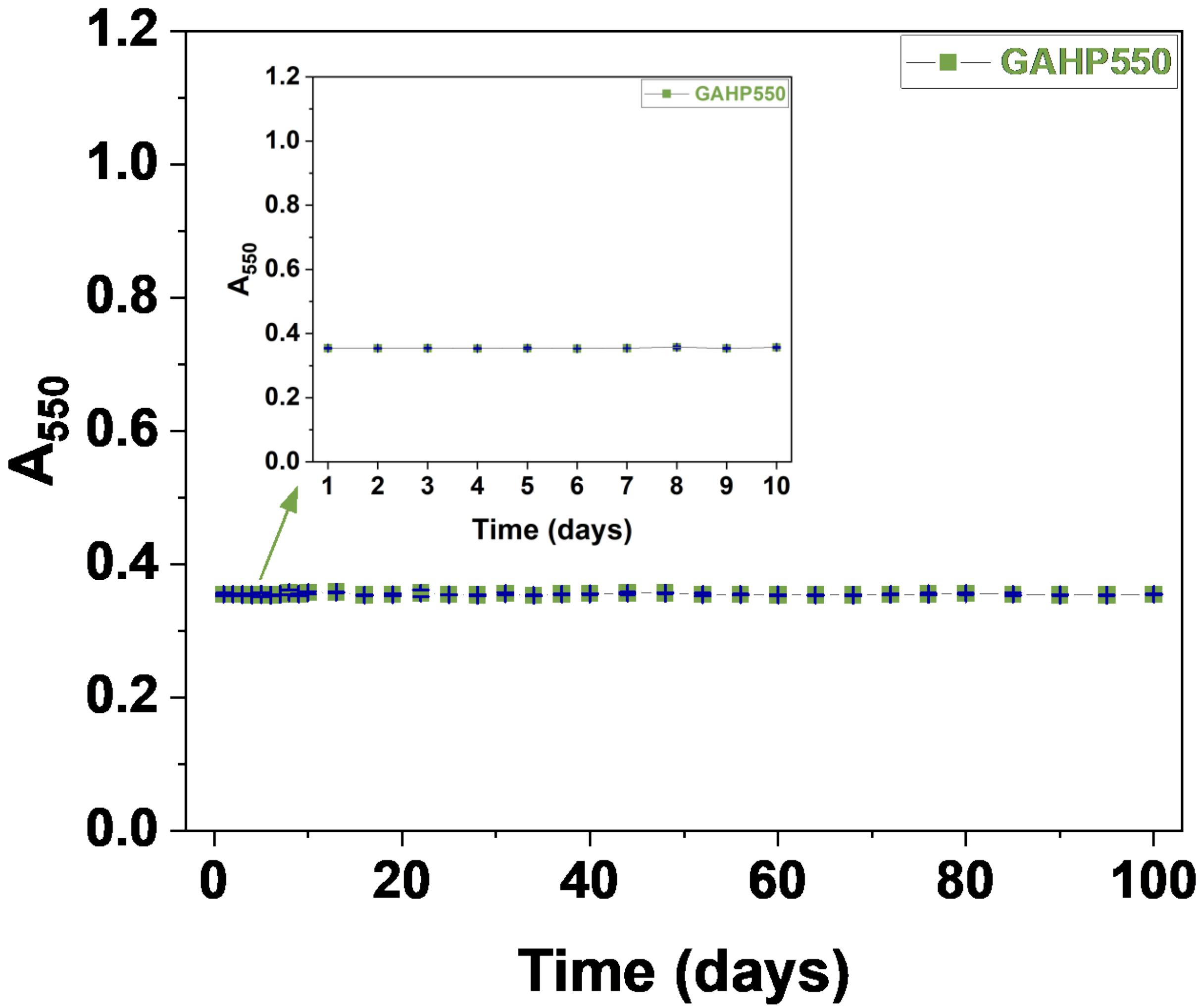

Figure S18. Stability evaluation of **GAHP550** (1.0×10^{-3} M, DMSO:H_2O 17:3, v/v, 0.1 M HEPES, pH 7.4) over a 100-day period. Inset: zoomed-in view of the 0-10 day stability profile. Error bars represent the standard deviation of three independent measurements (n = 3).

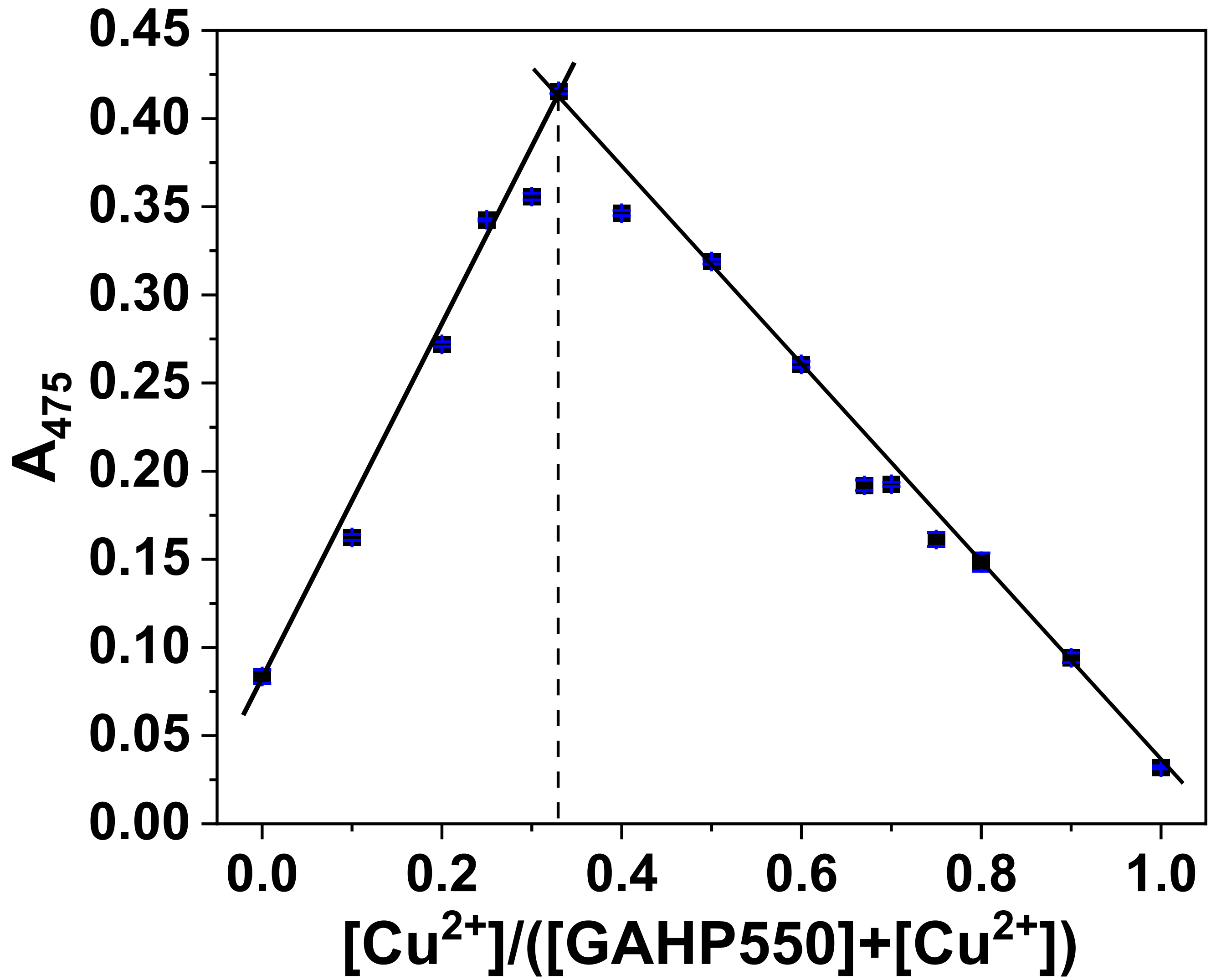

Figure S19. Job's Plot (1.0×10^{-4} M). Error bars represent the standard deviation of three independent measurements (n = 3).

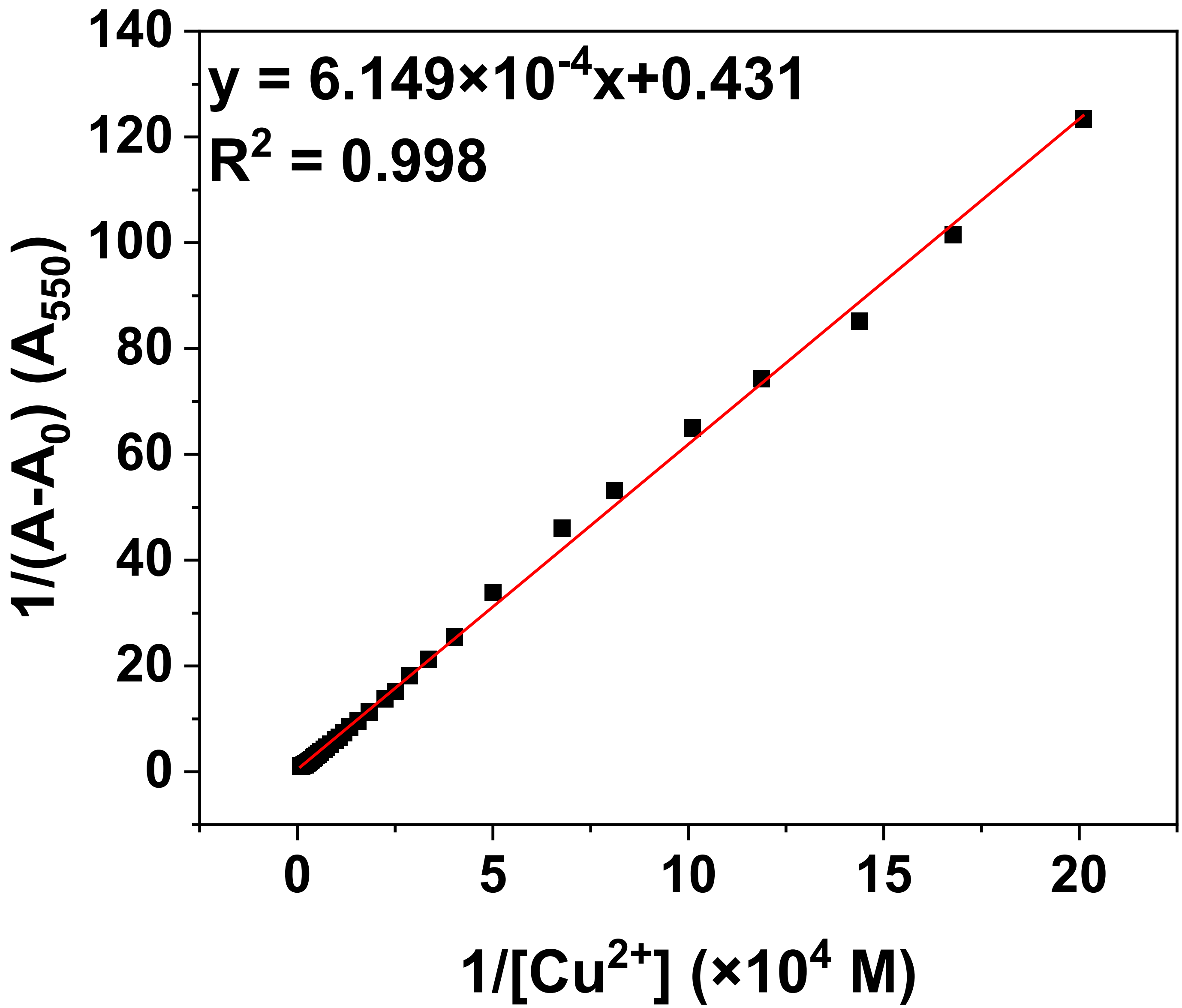

Figure S20. Benesi-Hildebrand linearization.

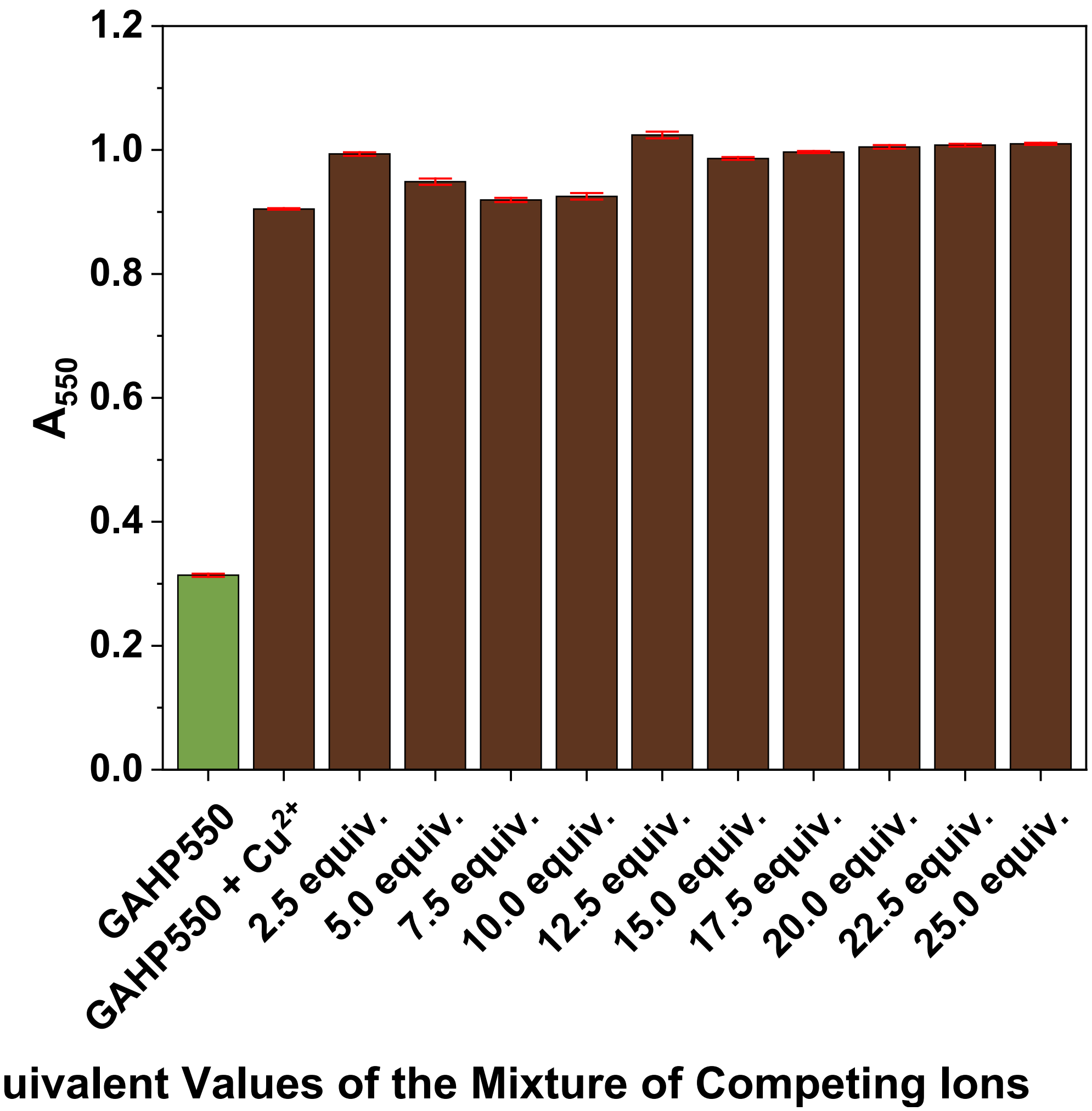

Figure S21. Interference study of **GAHP550** (1.0×10^{-3} M) upon addition of Cu^{2+} in the presence of varying equivalents (2.5–25.0 equiv.) of competing ions (Li^+, Na^+, K^+, Rb^+, Cs^+, NH_4^+, Mg^{2+}, Ca^{2+}, Sr^{2+}, Ba^{2+}, Cr^{3+}, Mn^{2+}, Fe^{2+}, Fe^{3+}, Co^{2+}, Ni^{2+}, Zn^{2+}, Cd^{2+}, Ag^+, Hg^{2+}, Al^{3+}, Sn^{2+}, Sn^{4+}, Pb^{2+}, N_2H_4, F^-, Cl^-, Br^-, I^-, OH^-, ClO^-, S^{2-}, SH^-, SO_3^{2-}, SO_4^{2-}, HSO_3^-, HSO_4^-, $S_2O_3^{2-}$, $S_2O_4^{2-}$, $S_2O_5^{2-}$, NO_2^-, NO_3^-, SCN^-, CH_3COO^-, HCO_3^-, CO_3^{2-}, $H_2PO_4^-$, HPO_4^{2-}, PO_4^{3-}, MnO_4^-). Medium: DMSO:H_2O (17:3, v/v, 0.1 M HEPES, pH 7.4). *Hg^{2+} ions were masked with fourfold $Na_2S_2O_3$. Error bars represent the standard deviation of three independent measurements (n = 3).

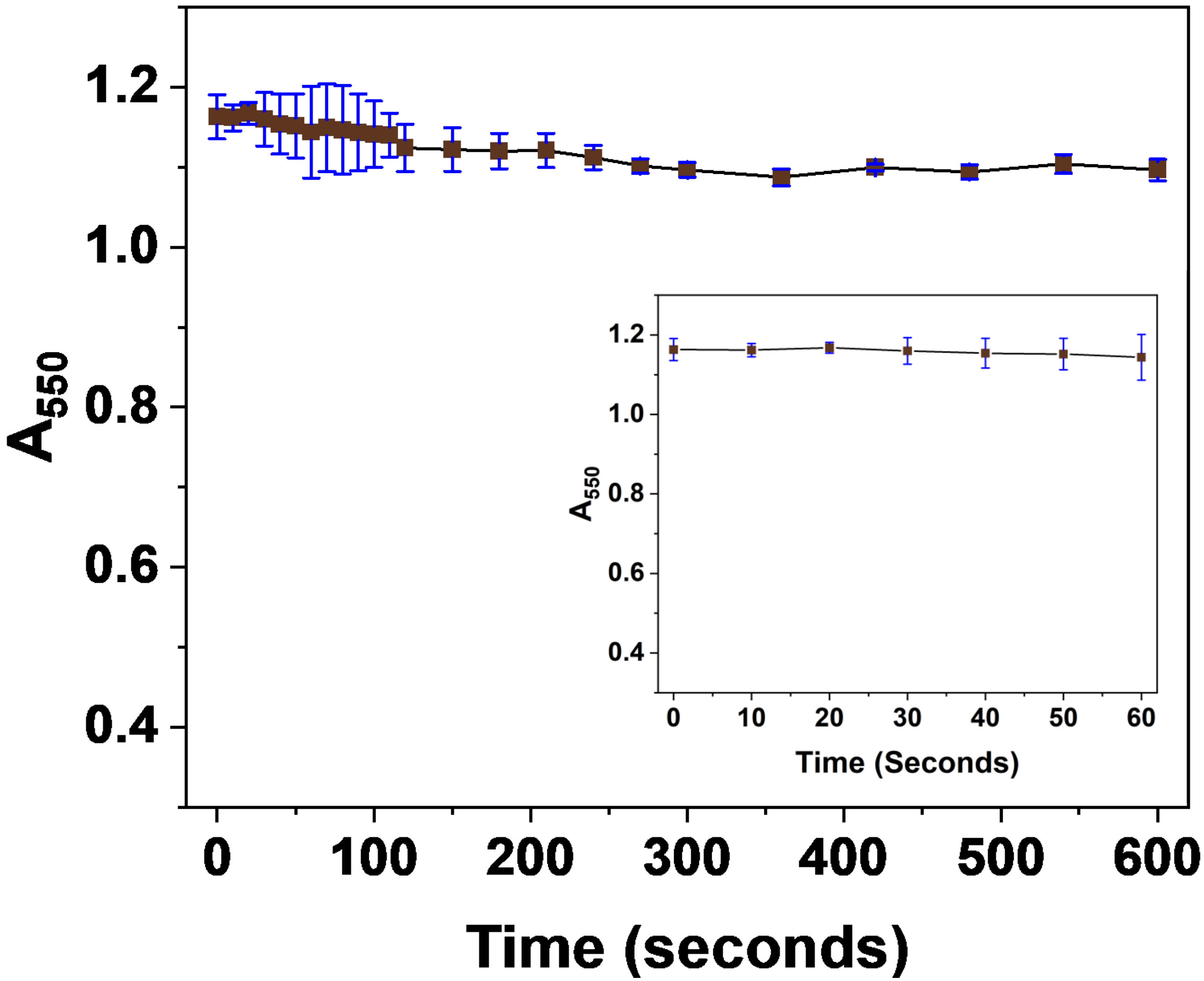

Figure S22. Time-dependent response of **GAHP550** (1.0×10^{-3} M) toward Cu^{2+} over 0–600 s. Inset: detailed view of the initial 0–60 s. Medium: DMSO:H_2O (17:3, v/v, 0.1 M HEPES, pH 7.4). Error bars represent the standard deviation of three independent measurements (n = 3).

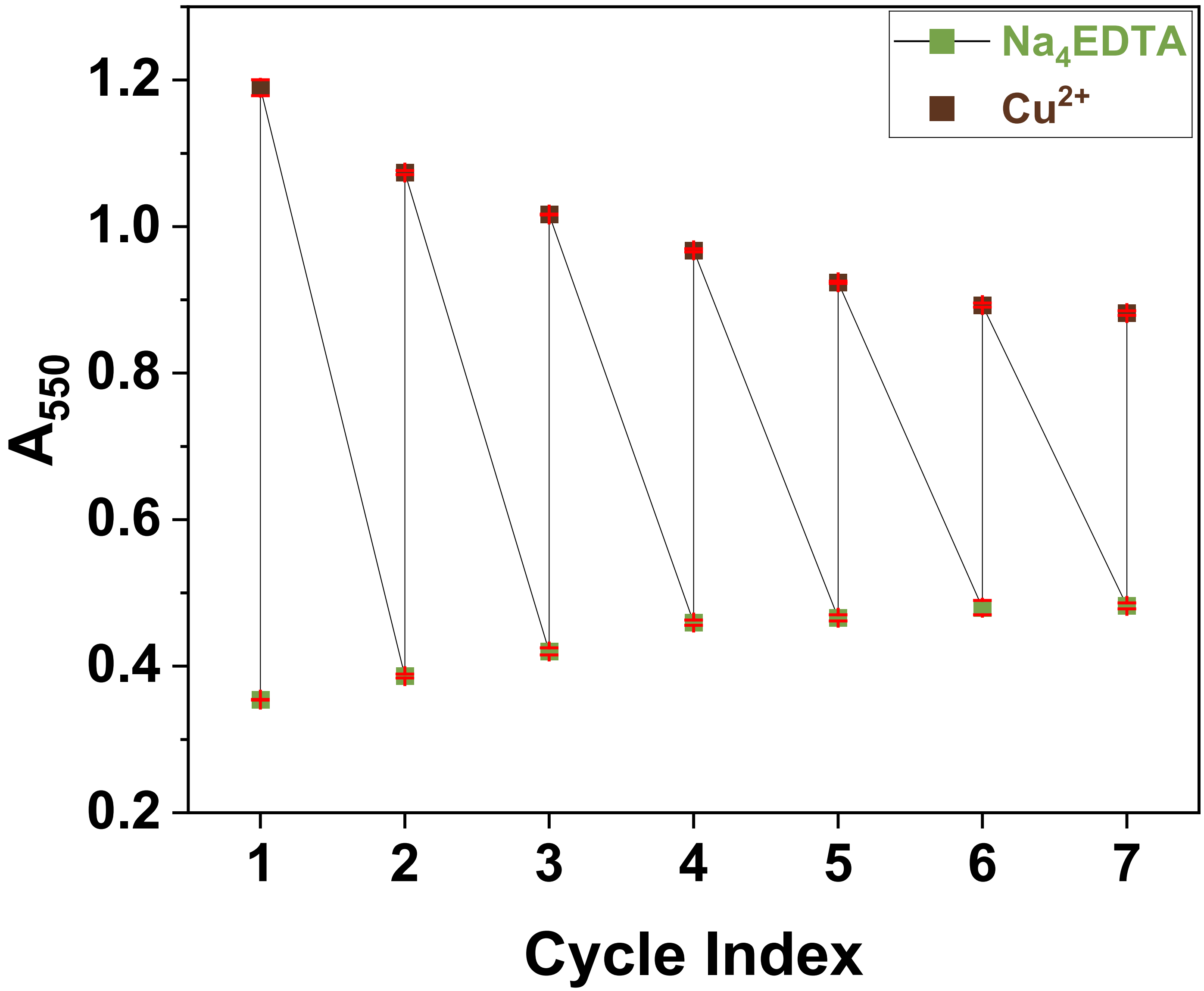

Figure S23. Reversibility of **GAHP550** (1.0×10^{-3} M) upon sequential addition of Cu^{2+} and Na_4EDTA. Medium: DMSO:H_2O (17:3, v/v, 0.1 M HEPES, pH 7.4). Error bars represent the standard deviation of three independent measurements (n = 3).

Sample Chromatograms

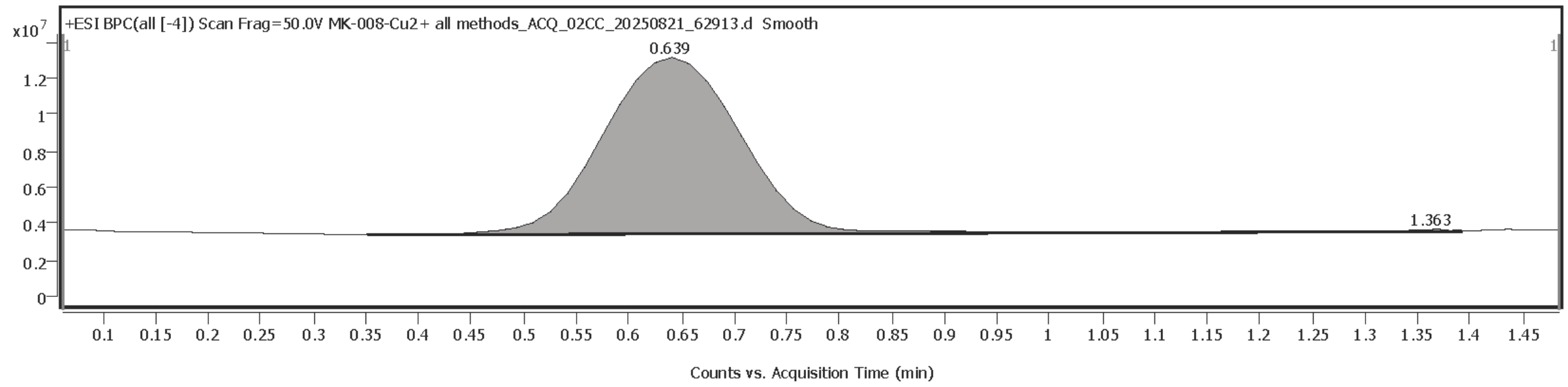

Chromatogram Peaks

Peak	Start	RT	End	Height	Area	Area %	SNR
1	0.351	0.639	1.044	8907224	87911824	100.00	
2	1.044	1.363	1.391	78270	968372	1.10	

Sample Spectra

+ Scan (rt: 0.062-1.484 min) **C42 H45 Cu N6**

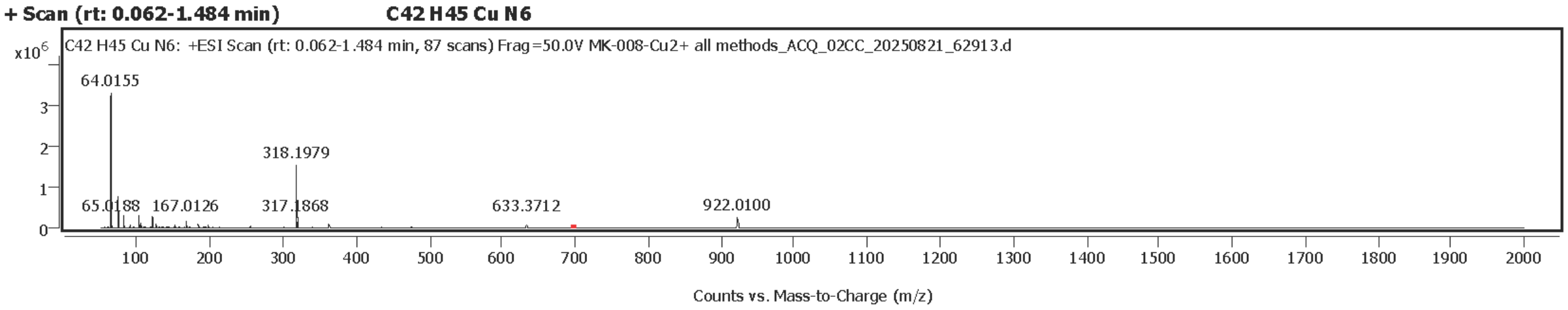

Spectrum Peaks

m/z	Z	Abund	Abund %	m/z (Calc)	Diff (ppm)	Ion Species	Formula	Ion Type
697.3079	1	75871	2.18	697.3074	0.68	(M+H)+	C42 H45 Cu N6	
698.3109	1	36885	1.06	698.3105	0.50	(M+H)+	C42 H45 Cu N6	
699.3089	1	37991	1.09	699.3073	2.33	(M+H)+	C42 H45 Cu N6	
64.0155	1	3476129	100.00					
65.0188	1	76986	2.21					
65.0596		40115	1.15					
74.0600		791385	22.77					
81.5210	2	302130	8.69					
83.0603		37071	1.07					
90.9764		73093	2.10					
102.0342	2	329007	9.46					
102.1275		43845	1.26					
102.5355	2	35501	1.02					
105.0422		116609	3.35					
121.0509		296337	8.52					
122.0962		74421	2.14					
125.9862		85830	2.47					
140.0679		35760	1.03					
151.0353		62191	1.79					
167.0126		192096	5.53					
171.0991		35864	1.03					
182.9852		88543	2.55					
185.1146		36711	1.06					
190.9791		35272	1.01					
197.0782		84830	2.44					
255.1203		56430	1.62					
316.1816	1	371313	10.68					
317.1868	1	121016	3.48					
318.1979	1	1549499	44.58					
319.2006	1	407905	11.73					
320.2041	1	43805	1.26					
362.2412		102962	2.96					
633.3712		75629	2.18					
922.0100	1	262161	7.54					
923.0126	1	52648	1.51					

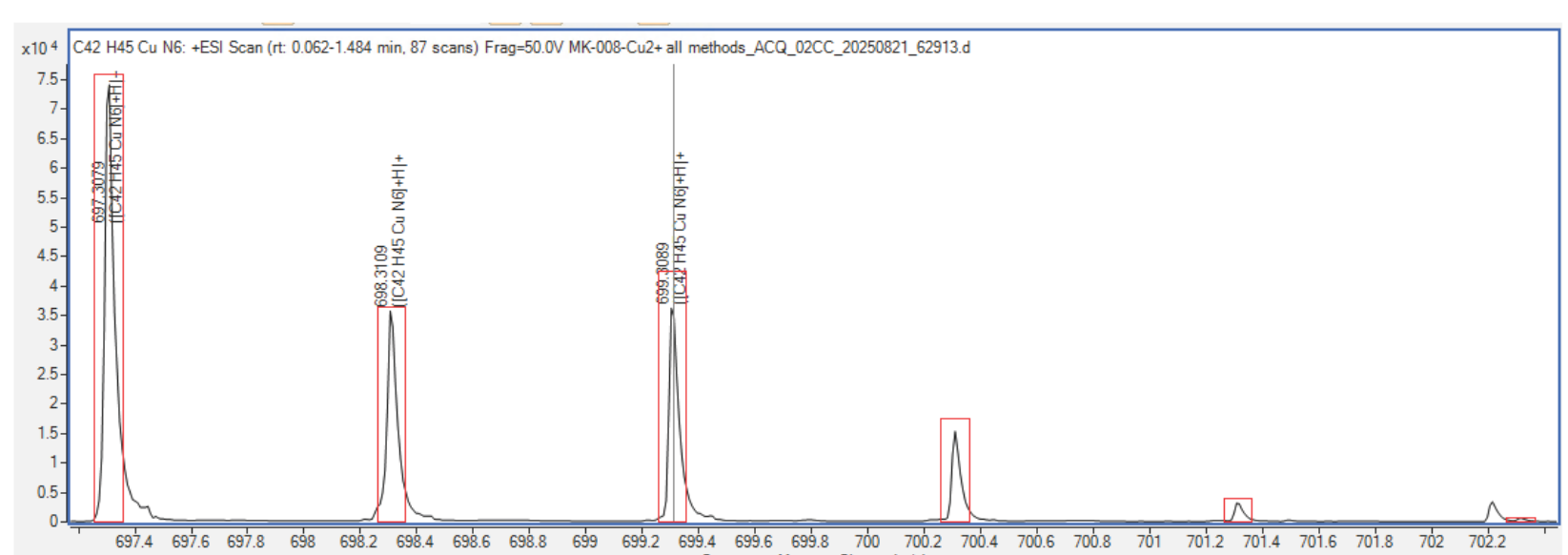

Spectrum Identification Table

Best	ID Source	Name	Formula	Species	m/z	Diff (ppm)	CAS	Score	Score (Lib)	Score (DB)	Score (MFG)	Lib/DB
Yes	MFG		C42 H45 Cu N6	(M+H)+	697.3079	1.13		96.53			96.53	

Figure S24. HRMS (ESI+) result of **Cu(GAHP550)$_2$** complex.

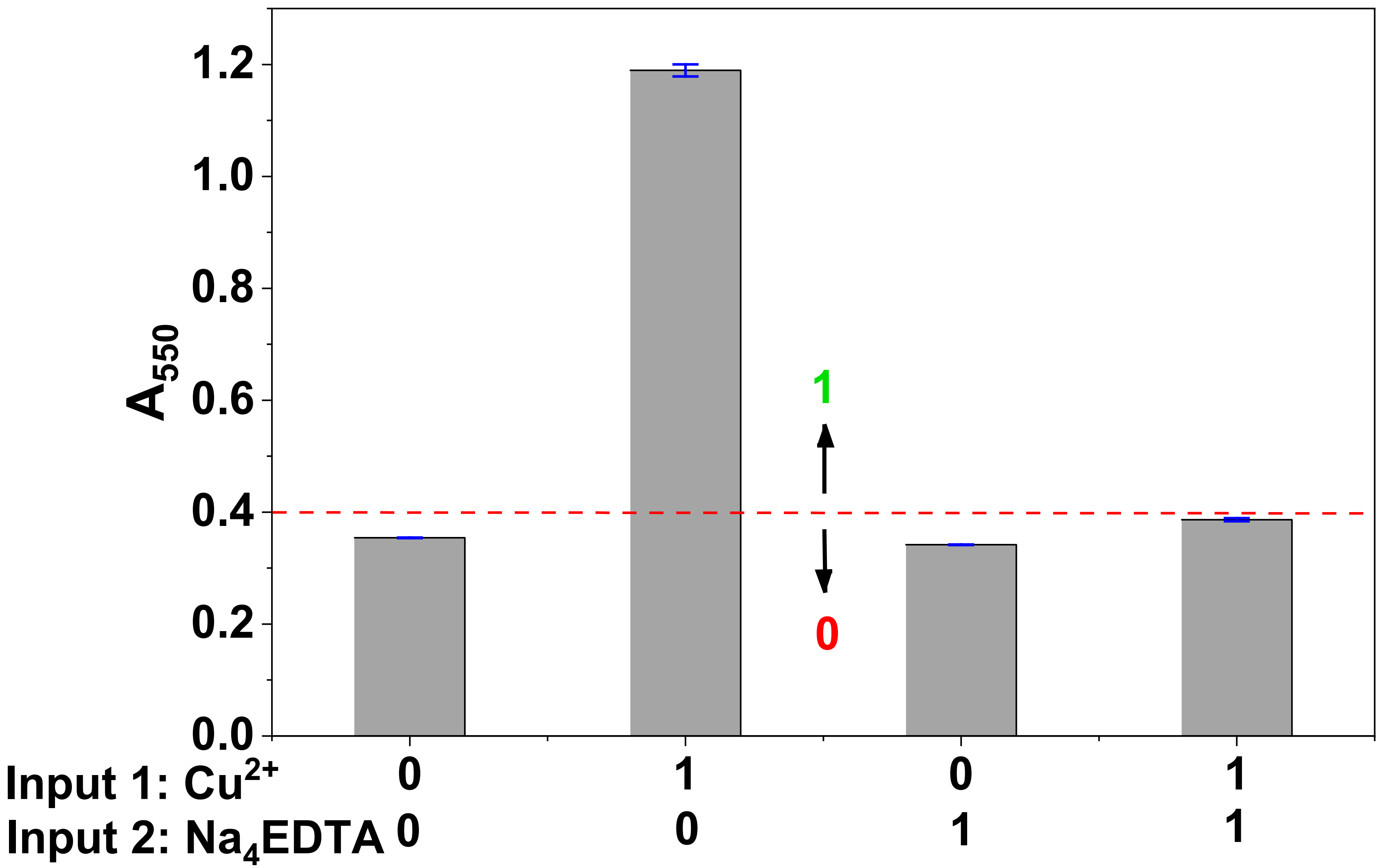

Figure S25. The graph of output signals at A_{550} nm for different inputs. Error bars represent the standard deviation of three independent measurements (n = 3).

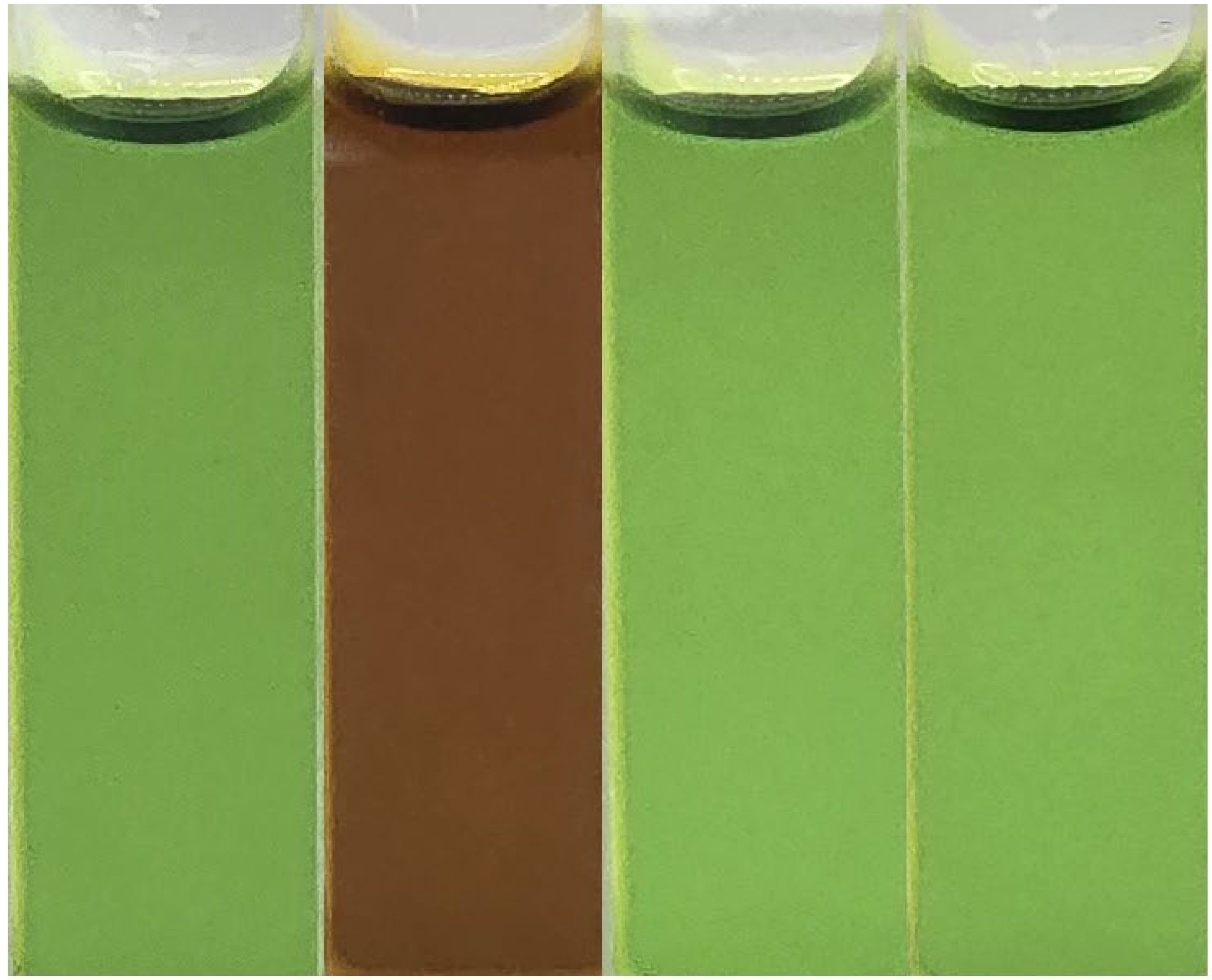

**Input 1: Cu^{2+}**	0	1	0	1
**Input 1: Na_4EDTA**	0	0	1	1
**Output: Color Change**	0	1	0	0

Figure S26. Output signals depicting the color change under different input conditions. Medium: DMSO:H_2O (17:3, v/v, 0.1 M HEPES, pH 7.4).

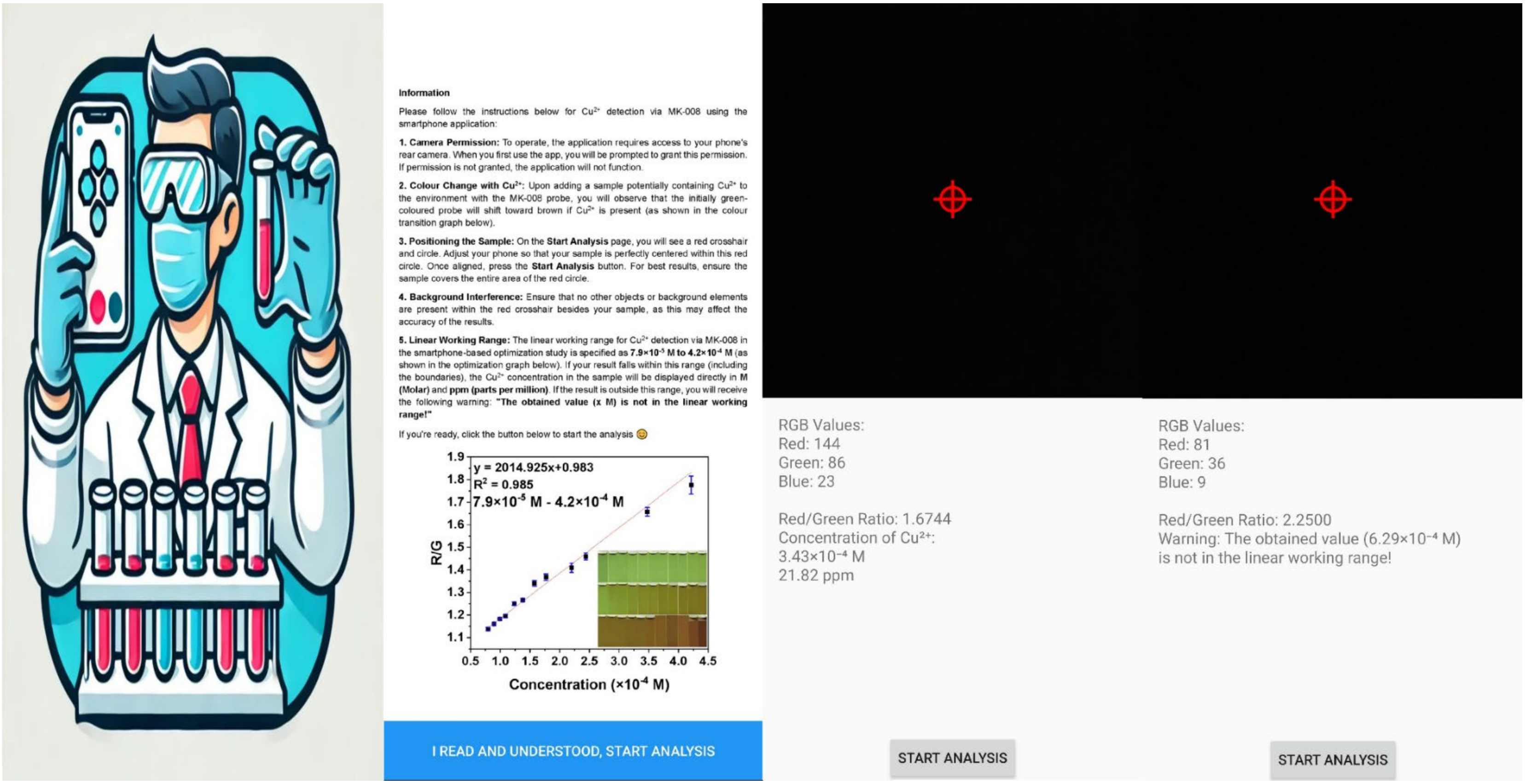

Figure S27. User interface of the "**GAHP550** Cu^{2+} Detector" mobile application.

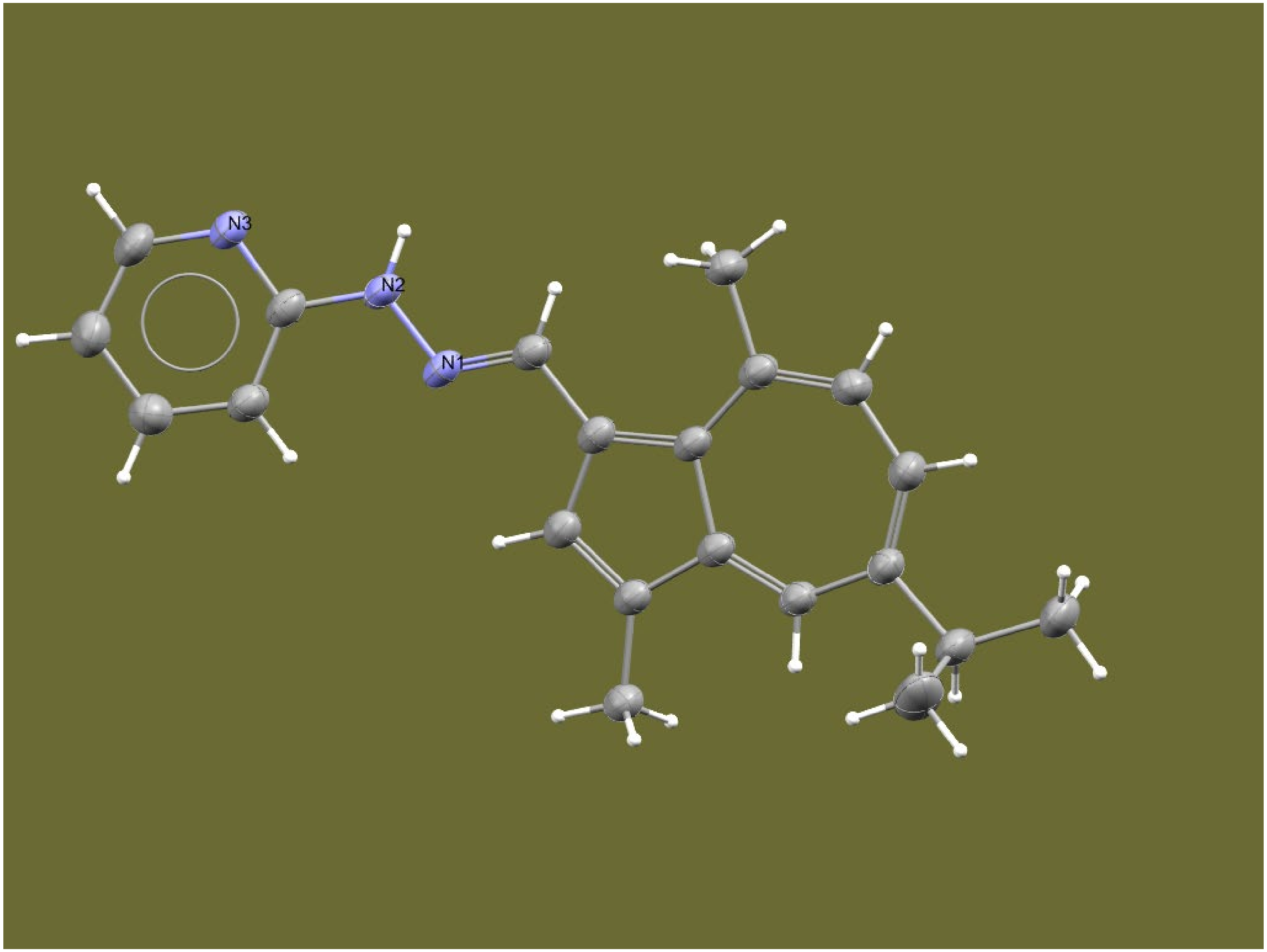

Figure S28. ORTEP diagram of **GAHP550.** Ellipsoids are drawn at 50% probability**.**

Table S1. Crystal data and structure refinement for **GAHP550**.

Identification code	2503754	
Empirical formula	$C_{21}H_{23}N_3$	
Formula weight	317.42	
Temperature	150.00(10) K	
Wavelength	1.54184 Å	
Crystal system	Triclinic	
Space group	P-1	
Unit cell dimensions	a = 5.1884(3) Å	a= 117.388(5)°.
	b = 13.1732(7) Å	b= 91.854(4)°.
	c = 14.2805(8) Å	g = 91.891(4)°.
Volume	864.92(9) Å^3	
Z	2	
Density (calculated)	1.219 Mg/m^3	
Absorption coefficient	0.560 mm^{-1}	
F(000)	340	
Crystal size	0.250 x 0.060 x 0.020 mm^3	
Theta range for data collection	3.491 to 79.284°.	
Index ranges	-6<=h<=5, -15<=k<=16, -18<=l<=17	
Reflections collected	8918	
Independent reflections	3444 [R(int) = 0.0287]	
Completeness to theta = 67.684°	98.6 %	
Absorption correction	Semi-empirical from equivalents	
Max. and min. transmission	1.00000 and 0.72834	
Refinement method	Full-matrix least-squares on F^2	
Data / restraints / parameters	3444 / 0 / 225	
Goodness-of-fit on F^2	1.040	
Final R indices [I>2sigma(I)]	R1 = 0.0467, wR2 = 0.1204	
R indices (all data)	R1 = 0.0537, wR2 = 0.1251	
Extinction coefficient	n/a	
Largest diff. peak and hole	0.190 and -0.204 e.Å^{-3}	

Table S2. Atomic coordinates (x10^4) and equivalent isotropic displacement parameters (Å^{2}x10^3) for **GAHP550**. U(eq) is defined as one third of the trace of the orthogonalized U^{ij} tensor.

	x	y	z	U(eq)
N(1)	5162(2)	1412(1)	4744(1)	31(1)
N(2)	7078(2)	676(1)	4607(1)	32(1)
N(3)	10098(2)	-501(1)	3563(1)	34(1)
C(1)	1036(3)	2768(1)	4936(1)	29(1)
C(2)	2378(3)	2787(1)	5837(1)	29(1)
C(3)	4430(3)	2006(1)	5686(1)	31(1)
C(4)	8135(3)	171(1)	3643(1)	30(1)
C(5)	7218(3)	349(1)	2796(1)	36(1)
C(6)	8387(3)	-176(1)	1856(1)	42(1)
C(7)	10428(3)	-871(2)	1758(1)	44(1)
C(8)	11177(3)	-997(1)	2628(1)	40(1)
C(9)	1365(3)	3645(1)	6749(1)	28(1)
C(10)	2001(3)	3982(1)	7825(1)	31(1)
C(11)	4029(3)	3357(1)	8106(1)	37(1)
C(12)	933(3)	4862(1)	8682(1)	35(1)
C(13)	-897(3)	5667(1)	8768(1)	36(1)
C(14)	-2292(3)	5802(1)	8001(1)	32(1)
C(15)	-4026(3)	6811(1)	8291(1)	39(1)
C(16)	-4837(4)	7374(2)	9422(1)	45(1)
C(17)	-2688(5)	7701(2)	8044(2)	60(1)
C(18)	-2171(3)	5075(1)	6908(1)	31(1)
C(19)	-672(3)	4168(1)	6359(1)	28(1)
C(20)	-782(3)	3572(1)	5232(1)	28(1)
C(21)	-2585(3)	3777(1)	4503(1)	33(1)

Table S3. Bond lengths [Å] for **GAHP550**.

N(1)-C(3)	1.2851(19)	C(11)-H(11B)	0.9800
N(1)-N(2)	1.3665(16)	C(11)-H(11C)	0.9800
N(2)-C(4)	1.3670(19)	C(12)-C(13)	1.413(2)
N(2)-H(2)	0.90(2)	C(12)-H(12)	0.9500
N(3)-C(8)	1.339(2)	C(13)-C(14)	1.376(2)
N(3)-C(4)	1.3452(18)	C(13)-H(13)	0.9500
C(1)-C(20)	1.3681(19)	C(14)-C(18)	1.411(2)
C(1)-C(2)	1.433(2)	C(14)-C(15)	1.5308(19)
C(1)-H(1)	0.9500	C(15)-C(16)	1.516(2)
C(2)-C(9)	1.4069(19)	C(15)-C(17)	1.525(3)
C(2)-C(3)	1.4564(19)	C(15)-H(15)	1.0000
C(3)-H(3)	0.9500	C(16)-H(16A)	0.9800
C(4)-C(5)	1.406(2)	C(16)-H(16B)	0.9800
C(5)-C(6)	1.369(2)	C(16)-H(16C)	0.9800
C(5)-H(5)	0.9500	C(17)-H(17A)	0.9800
C(6)-C(7)	1.391(2)	C(17)-H(17B)	0.9800
C(6)-H(6)	0.9500	C(17)-H(17C)	0.9800
C(7)-C(8)	1.373(2)	C(18)-C(19)	1.374(2)
C(7)-H(7)	0.9500	C(18)-H(18)	0.9500
C(8)-H(8)	0.9500	C(19)-C(20)	1.427(2)
C(9)-C(10)	1.413(2)	C(20)-C(21)	1.498(2)
C(9)-C(19)	1.5055(18)	C(21)-H(21A)	0.9800
C(10)-C(12)	1.388(2)	C(21)-H(21B)	0.9800
C(10)-C(11)	1.5104(19)	C(21)-H(21C)	0.9800
C(11)-H(11A)	0.9800		

Table S4. Bond angles [°] for **GAHP550**.

C(3)-N(1)-N(2)	117.53(13)	C(12)-C(10)-C(11)	115.01(13)
N(1)-N(2)-C(4)	117.81(13)	C(9)-C(10)-C(11)	118.97(13)
N(1)-N(2)-H(2)	120.9(12)	C(10)-C(11)-H(11A)	109.5
C(4)-N(2)-H(2)	119.9(12)	C(10)-C(11)-H(11B)	109.5
C(8)-N(3)-C(4)	116.63(13)	H(11A)-C(11)-H(11B)	109.5
C(20)-C(1)-C(2)	111.27(13)	C(10)-C(11)-H(11C)	109.5
C(20)-C(1)-H(1)	124.4	H(11A)-C(11)-H(11C)	109.5
C(2)-C(1)-H(1)	124.4	H(11B)-C(11)-H(11C)	109.5
C(9)-C(2)-C(1)	107.98(12)	C(10)-C(12)-C(13)	132.97(14)
C(9)-C(2)-C(3)	132.34(13)	C(10)-C(12)-H(12)	113.5
C(1)-C(2)-C(3)	119.67(13)	C(13)-C(12)-H(12)	113.5
N(1)-C(3)-C(2)	117.85(13)	C(14)-C(13)-C(12)	130.71(15)
N(1)-C(3)-H(3)	121.1	C(14)-C(13)-H(13)	114.6
C(2)-C(3)-H(3)	121.1	C(12)-C(13)-H(13)	114.6
N(3)-C(4)-N(2)	115.63(13)	C(13)-C(14)-C(18)	123.82(13)
N(3)-C(4)-C(5)	122.57(14)	C(13)-C(14)-C(15)	120.98(13)
N(2)-C(4)-C(5)	121.80(13)	C(18)-C(14)-C(15)	115.15(13)
C(6)-C(5)-C(4)	118.57(14)	C(16)-C(15)-C(17)	109.91(15)
C(6)-C(5)-H(5)	120.7	C(16)-C(15)-C(14)	115.49(13)
C(4)-C(5)-H(5)	120.7	C(17)-C(15)-C(14)	109.06(13)
C(5)-C(6)-C(7)	119.77(15)	C(16)-C(15)-H(15)	107.3
C(5)-C(6)-H(6)	120.1	C(17)-C(15)-H(15)	107.3
C(7)-C(6)-H(6)	120.1	C(14)-C(15)-H(15)	107.3
C(8)-C(7)-C(6)	117.38(15)	C(15)-C(16)-H(16A)	109.5
C(8)-C(7)-H(7)	121.3	C(15)-C(16)-H(16B)	109.5
C(6)-C(7)-H(7)	121.3	H(16A)-C(16)-H(16B)	109.5
N(3)-C(8)-C(7)	125.08(14)	C(15)-C(16)-H(16C)	109.5
N(3)-C(8)-H(8)	117.5	H(16A)-C(16)-H(16C)	109.5
C(7)-C(8)-H(8)	117.5	H(16B)-C(16)-H(16C)	109.5
C(2)-C(9)-C(10)	129.85(13)	C(15)-C(17)-H(17A)	109.5
C(2)-C(9)-C(19)	105.64(12)	C(15)-C(17)-H(17B)	109.5
C(10)-C(9)-C(19)	124.51(12)	H(17A)-C(17)-H(17B)	109.5
C(12)-C(10)-C(9)	126.01(13)	C(15)-C(17)-H(17C)	109.5

H(17A)-C(17)-H(17C)	109.5	C(1)-C(20)-C(21)	125.97(13)
H(17B)-C(17)-H(17C)	109.5	C(19)-C(20)-C(21)	126.35(12)
C(19)-C(18)-C(14)	131.39(14)	C(20)-C(21)-H(21A)	109.5
C(19)-C(18)-H(18)	114.3	C(20)-C(21)-H(21B)	109.5
C(14)-C(18)-H(18)	114.3	H(21A)-C(21)-H(21B)	109.5
C(18)-C(19)-C(20)	122.18(13)	C(20)-C(21)-H(21C)	109.5
C(18)-C(19)-C(9)	130.34(14)	H(21A)-C(21)-H(21C)	109.5
C(20)-C(19)-C(9)	107.43(12)	H(21B)-C(21)-H(21C)	109.5
C(1)-C(20)-C(19)	107.67(12)		

Table S5. Anisotropic displacement parameters ($Å^2x10^3$) for **GAHP550**. The anisotropic displacement factor exponent takes the form: $-2p^2[h^2 a^{*2}U^{11} + ... + 2 h k a^* b^* U^{12}]$.

	U^{11}	U^{22}	U^{33}	U^{23}	U^{13}	U^{12}
N(1)	26(1)	27(1)	42(1)	17(1)	5(1)	8(1)
N(2)	30(1)	31(1)	39(1)	18(1)	6(1)	12(1)
N(3)	29(1)	32(1)	42(1)	17(1)	6(1)	10(1)
C(1)	27(1)	27(1)	34(1)	15(1)	4(1)	3(1)
C(2)	26(1)	27(1)	37(1)	17(1)	5(1)	4(1)
C(3)	29(1)	30(1)	38(1)	18(1)	3(1)	7(1)
C(4)	26(1)	25(1)	39(1)	14(1)	4(1)	4(1)
C(5)	35(1)	33(1)	40(1)	16(1)	2(1)	11(1)
C(6)	45(1)	44(1)	38(1)	20(1)	2(1)	9(1)
C(7)	40(1)	50(1)	40(1)	17(1)	10(1)	13(1)
C(8)	33(1)	42(1)	45(1)	18(1)	9(1)	16(1)
C(9)	24(1)	28(1)	38(1)	18(1)	4(1)	6(1)
C(10)	26(1)	33(1)	40(1)	20(1)	4(1)	7(1)
C(11)	37(1)	40(1)	37(1)	20(1)	2(1)	14(1)
C(12)	35(1)	40(1)	34(1)	20(1)	3(1)	11(1)
C(13)	36(1)	38(1)	34(1)	16(1)	7(1)	12(1)
C(14)	30(1)	33(1)	37(1)	17(1)	7(1)	10(1)
C(15)	40(1)	39(1)	37(1)	16(1)	4(1)	18(1)
C(16)	45(1)	43(1)	43(1)	16(1)	10(1)	21(1)
C(17)	79(1)	47(1)	70(1)	37(1)	26(1)	31(1)
C(18)	27(1)	33(1)	36(1)	19(1)	4(1)	8(1)
C(19)	23(1)	29(1)	37(1)	19(1)	4(1)	4(1)
C(20)	24(1)	28(1)	36(1)	17(1)	4(1)	4(1)
C(21)	29(1)	35(1)	36(1)	18(1)	3(1)	8(1)

Table S6. Hydrogen coordinates ($x10^4$) and isotropic displacement parameters ($Å^2x10^3$) for **GAHP550**.

	x	y	z	U(eq)
H(2)	7870(40)	648(15)	5164(15)	40(5)
H(1)	1366	2262	4224	35
H(3)	5202	1938	6265	37
H(5)	5817	824	2874	43
H(6)	7806	-66	1273	50
H(7)	11271	-1244	1114	53
H(8)	12568	-1473	2561	48
H(11A)	3482	2544	7805	56
H(11B)	4251	3684	8876	56
H(11C)	5671	3437	7820	56
H(12)	1559	4937	9345	42
H(13)	-1217	6203	9471	43
H(15)	-5636	6515	7822	47
H(16A)	-6074	7954	9513	67
H(16B)	-3315	7737	9902	67
H(16C)	-5646	6793	9583	67
H(17A)	-2247	7339	7302	91
H(17B)	-1108	8016	8498	91
H(17C)	-3848	8318	8172	91
H(18)	-3331	5243	6475	37
H(21A)	-2256	3247	3769	49
H(21B)	-2305	4567	4619	49
H(21C)	-4373	3651	4642	49

Table S7. Torsion angles [°] for **GAHP550**.

C(3)-N(1)-N(2)-C(4)	-170.39(13)	C(9)-C(10)-C(12)-C(13)	-2.5(3)
C(20)-C(1)-C(2)-C(9)	-0.02(16)	C(11)-C(10)-C(12)-C(13)	177.16(16)
C(20)-C(1)-C(2)-C(3)	-178.87(12)	C(10)-C(12)-C(13)-C(14)	2.7(3)
N(2)-N(1)-C(3)-C(2)	-179.67(11)	C(12)-C(13)-C(14)-C(18)	2.2(3)
C(9)-C(2)-C(3)-N(1)	-170.80(14)	C(12)-C(13)-C(14)-C(15)	-175.12(16)
C(1)-C(2)-C(3)-N(1)	7.7(2)	C(13)-C(14)-C(15)-C(16)	-20.6(2)
C(8)-N(3)-C(4)-N(2)	-179.45(13)	C(18)-C(14)-C(15)-C(16)	161.84(15)
C(8)-N(3)-C(4)-C(5)	0.5(2)	C(13)-C(14)-C(15)-C(17)	103.68(18)
N(1)-N(2)-C(4)-N(3)	176.86(12)	C(18)-C(14)-C(15)-C(17)	-73.83(19)
N(1)-N(2)-C(4)-C(5)	-3.1(2)	C(13)-C(14)-C(18)-C(19)	-4.1(3)
N(3)-C(4)-C(5)-C(6)	-0.5(2)	C(15)-C(14)-C(18)-C(19)	173.29(15)
N(2)-C(4)-C(5)-C(6)	179.38(14)	C(14)-C(18)-C(19)-C(20)	-177.10(14)
C(4)-C(5)-C(6)-C(7)	0.2(2)	C(14)-C(18)-C(19)-C(9)	-0.1(3)
C(5)-C(6)-C(7)-C(8)	0.1(3)	C(2)-C(9)-C(19)-C(18)	-176.28(14)
C(4)-N(3)-C(8)-C(7)	-0.1(2)	C(10)-C(9)-C(19)-C(18)	4.4(2)
C(6)-C(7)-C(8)-N(3)	-0.1(3)	C(2)-C(9)-C(19)-C(20)	1.09(14)
C(1)-C(2)-C(9)-C(10)	178.63(14)	C(10)-C(9)-C(19)-C(20)	-178.24(13)
C(3)-C(2)-C(9)-C(10)	-2.7(3)	C(2)-C(1)-C(20)-C(19)	0.73(15)
C(1)-C(2)-C(9)-C(19)	-0.65(14)	C(2)-C(1)-C(20)-C(21)	-178.51(13)
C(3)-C(2)-C(9)-C(19)	177.99(14)	C(18)-C(19)-C(20)-C(1)	176.52(13)
C(2)-C(9)-C(10)-C(12)	178.52(15)	C(9)-C(19)-C(20)-C(1)	-1.11(15)
C(19)-C(9)-C(10)-C(12)	-2.3(2)	C(18)-C(19)-C(20)-C(21)	-4.2(2)
C(2)-C(9)-C(10)-C(11)	-1.2(2)	C(9)-C(19)-C(20)-C(21)	178.13(13)
C(19)-C(9)-C(10)-C(11)	177.99(13)		

Table S8. Hydrogen bonds for **GAHP550** [Å and °].

D-H...A	d(D-H)	d(H...A)	d(D...A)	<(DHA)
N(2)-H(2)...N(3)#1	0.90(2)	2.16(2)	3.0588(19)	176.5(17)

Symmetry transformations used to generate equivalent atoms:
#1 -x+2,-y,-z+1

Molecular Modelling

Molecular modelling was carried out using Orca 6.1. All structures were optimised using DFT with semi-empirical (XTB, GFN2) pre-optimization of the starting geometry. The DFT method used was BHLYP with the atom-pairwise dispersion correction D3 with Becke-Johnson damping using def2-TZVP basis set and def2/J auxiliary basis set [2-7]. The geometry optimisations were performed using the SMD solvent (DMSO or H_2O) effect model. The BHLYP functional was shown [8] to perform well in the modelling of Cu(II) complexes. Four different starting geometries have been optimised to structures **I**, **II**, **III**, **IV** (Fig S29) and their UV-Vis spectra predicted via TDDFT. The computed TDDFT spectra were compared with the experimental spectrum using the similarity factor in SpecDis [9] which finds the optimal scaling and shift factors between the two spectra via a cross-section algorithm. Of these structures, structure **I** had the lowest energy and the best similarity factor of the predicted UV-Vis spectrum when compared with the experimental one (Table S9).

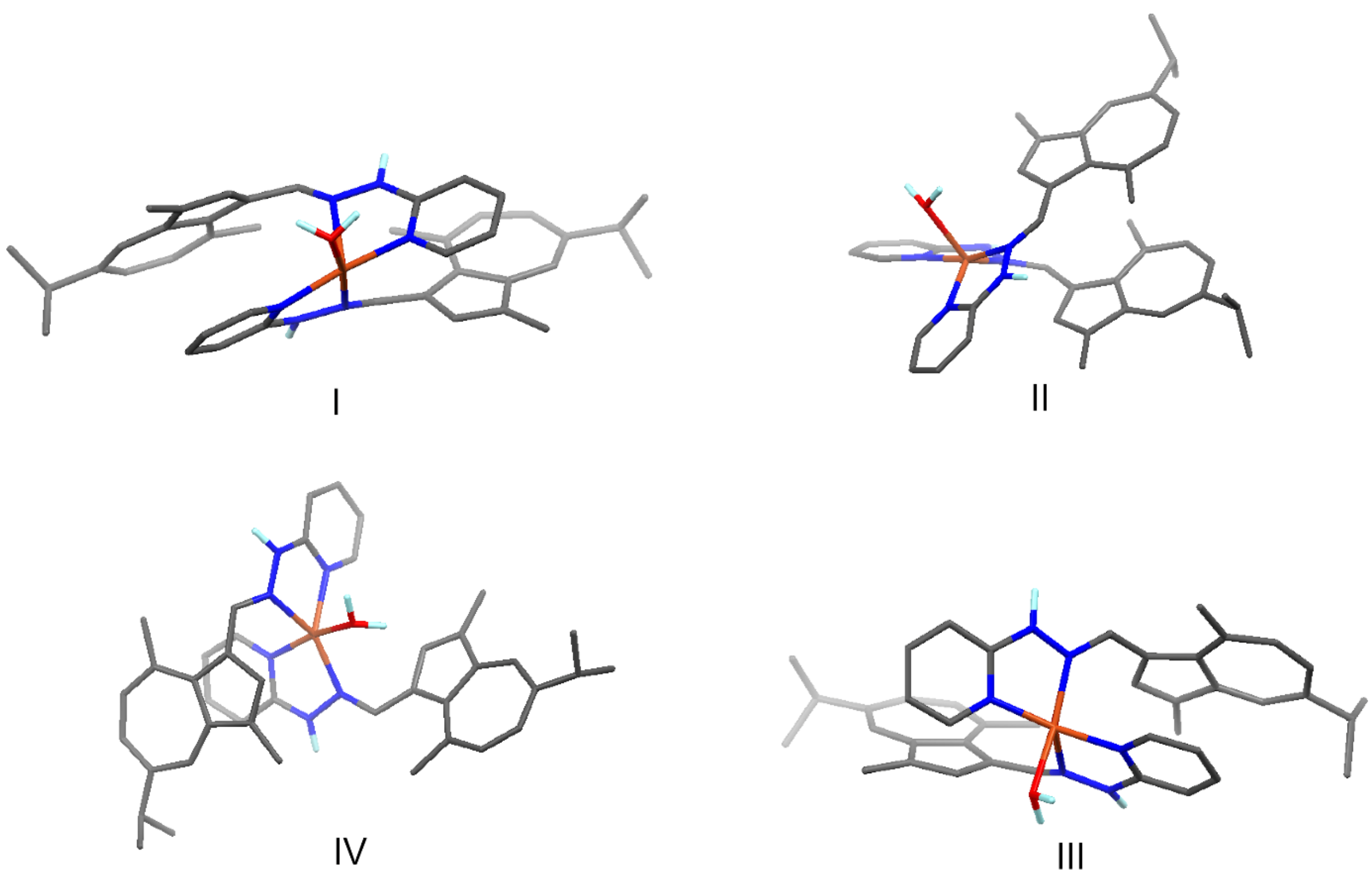

Figure S29. Geometry optimised structures of **Cu(GAHP550)₂**.

Table S9. Molecular modelling energy and similarity factors.

Structure	ΔE (kcal/mol)	Similarity factor
**I**	0	0.98
**II**	1.22	0.97
**III**	3.95	0.97
**IV**	7.67	0.94

References

(1) Ghazvini Zadeh, E. H.; Woodward, A. W.; Richardson, D.; Bondar, M. V.; Belfield, K. D. Stimuli-Responsive Cyclopenta[*ef*]heptalenes: Synthesis and Optical Properties. *Eur. J. Org. Chem.* **2015**, *2015* (10), 2271-2276. DOI: 10.1002/ejoc.201500059.

(2) Neese, F. Software update: the ORCA program system—version 6.0. *Wiley Interdiscip. Rev. Comput. Mol. Sci.* **2025**, *15* (2), e70019. DOI: 10.1002/wcms.70019.

(3) Becke, A. D. Density-functional exchange-energy approximation with correct asymptotic behavior. *Phys. Rev. A* **1988**, *38* (6), 3098. DOI: 10.1103/PhysRevA.38.3098.

(4) Lee, C.; Yang, W.; Parr, R. G. Development of the Colle-Salvetti correlation-energy formula into a functional of the electron density. *Phys. Rev. B* **1988**, *37* (2), 785. DOI: 10.1103/PhysRevB.37.785.

(5) Marenich, A. V.; Cramer, C. J.; Truhlar, D. G. Universal solvation model based on solute electron density and on a continuum model of the solvent defined by the bulk dielectric constant and atomic surface tensions. *J. Phys. Chem. B* **2009**, *113* (18), 6378-6396. DOI: 10.1021/jp810292n.

(6) Weigend, F.; Ahlrichs, R. Balanced basis sets of split valence, triple zeta valence and quadruple zeta valence quality for H to Rn: Design and assessment of accuracy. *Phys. Chem. Chem. Phys.* **2005**, *7* (18), 3297-3305. DOI: 10.1039/B508541A.

(7) Grimme, S.; Ehrlich, S.; Goerigk, L. Effect of the damping function in dispersion corrected density functional theory. *J. Comput. Chem.* **2011**, *32* (7), 1456-1465. DOI: 10.1002/jcc.21759.

(8) Solier, S.; Müller, S.; Cañeque, T.; Versini, A.; Mansart, A.; Sindikubwabo, F.; Baron, L.; Emam, L.; Gestraud, P.; Pantoș, G. D. A druggable copper-signalling pathway that drives inflammation. *Nature* **2023**, *617* (7960), 386-394. DOI: 10.1038/s41586-023-06017-4.

(9) Bruhn, T.; Schaumlöffel, A.; Hemberger, Y.; Bringmann, G. SpecDis: Quantifying the comparison of calculated and experimental electronic circular dichroism spectra. *Chirality* **2013**, *25* (4), 243-249. DOI: 10.1002/chir.22138.
